# Circulating tumor cell number and endocrine therapy index in ER positive metastatic breast cancer patients

**DOI:** 10.1038/s41523-021-00281-1

**Published:** 2021-06-11

**Authors:** Costanza Paoletti, Meredith M. Regan, Samuel M. Niman, Emily M. Dolce, Elizabeth P. Darga, Minetta C. Liu, P. Kelly Marcom, Lowell L. Hart, John W. Smith, Karen L. Tedesco, Eitan Amir, Ian E. Krop, Angela M. DeMichele, Pamela J. Goodwin, Margaret Block, Kimberly Aung, Martha E. Brown, Robert T. McCormack, Daniel F. Hayes

**Affiliations:** 1 Department of Internal Medicine, Division of Hematology/Oncology, University of Michigan Rogel Cancer Center, Ann Arbor, MI USA; 2 Division of Biostatistics, Dana-Farber Cancer Institute, Boston, MA USA; 3 Department of Oncology, Division of Medical Oncology, and Department of Laboratory Medicine and Pathology, Division of Anatomic Pathology, Mayo Clinic, Rochester, MN USA; 4 Department of Medicine, Division of Medical Oncology, Duke Cancer Institute, Durham, NC USA; 5 Florida Cancer Specialists/Wake Forest School of Medicine, Fort Myers, FL USA; 6 US Oncology Research, Compass Oncology, Portland, OR USA; 7 New York Oncology Hematology, US Oncology Network, Albany, NY USA; 8 Division of Medical Oncology and Hematology, Princess Margaret Cancer Centre, Toronto, ON Canada; 9 Department of Medical Oncology, Division of Breast Oncology, Dana-Farber Cancer Institute, Boston, MA USA; 10 Department of Medicine, Division of Hematology/Oncology, University of Pennsylvania, Philadelphia, PA USA; 11 Department of Medicine, Sinai Health System, University of Toronto, Toronto, ON Canada; 12 Nebraska Cancer Specialists, Omaha, NE USA; 13 Janssen Pharmaceuticals, Inc., Raritan, NJ USA; 14 Present Address: Currently at EISAI, Inc., Woodcliff Lake, NJ USA

**Keywords:** Translational research, Tumour biomarkers, Prognostic markers, Breast cancer, Tumour heterogeneity

## Abstract

Circulating tumor cells (CTC) are prognostic in metastatic breast cancer (MBC). The CTC-endocrine therapy index (CTC-ETI), consisting of CTC-ER (estrogen receptor), BCL2, human epidermal growth factor receptor (HER2), and Ki67 expression, might predict resistance to endocrine therapy (ET) in patients with ER-positive MBC. One hundred twenty-one patients with ER-positive/HER2-negative MBC initiating a new ET after ≥1 lines of ET were enrolled in a prospective, multi-institutional clinical trial. CTC-ETI and clinical/imaging follow-up were performed at baseline and serial time points. Progression-free survival (PFS) and rapid progression (RP; determined at the 3-month time point) were primary endpoints. Associations with clinical outcomes used logrank and Fisher’s exact tests. At baseline, 36% (38/107) of patients had ≥5 CTC/7.5 ml whole blood (WB). Patients with ≥5 vs. <5 CTC/7.5 ml WB had significantly worse PFS (median 3.3 vs. 5.9 months, *P* = 0.03). Elevated CTC at 1 month was associated with even worse PFS (1.9 vs. 5.0 months from the 1-month sample, *P* < 0.001). Low, intermediate, and high CTC-ETI were observed in 71 (66%), 8 (8%), and 28 (26%) patients, with median PFS of 6.9, 8.5, and 2.8 months, respectively (*P* = 0.008). Patients with high vs. low CTC and CTC-ETI more frequently experienced RP (CTC: 66% vs. 41%; *P* = 0.03; CTC-ETI: 79% vs. 40%; *P* = 0.002). In conclusion, CTC enumeration and the CTC-ETI assay are prognostic at baseline and follow-up in patients with ER-positive/HER2-negative MBC starting new ET. CTC at first follow-up might identify a group of patients with ER-positive MBC that could forego ET, but CTC-ETI did not contribute further.

## Introduction

Hormone receptor (HR) status has been used to guide endocrine therapy (ET) of patients with metastatic breast cancer (MBC). Patients with estrogen (ER) and progesterone (PgR) receptor-negative MBC have almost no chance of benefit from ET, and therefore are treated with more toxic, but more likely beneficial, chemotherapy^1^. However, only 30–50% of HR-positive MBC patients benefit from ET^2^, and 15–30% of such patients progress in the first 2 or 3 months of therapy, regardless of whether they have been treated with first or later lines of ET^2^. Currently, there is no validated method to identify which HR-positive patients are unlikely to respond to ET and would be better treated with other therapies, such as combination ET and other targeted treatments or with chemotherapy.

One potential method to identify refractory patients would be to perform investigational tissue biopsies to determine serial HR status at each critical clinical time point. However, serial biopsies are impractical, costly, and invasive for patients. In this regard, use of circulating tumor biomarkers, designated as “liquid biopsies” might provide a more convenient and practical surrogate for tissue biopsies^3^. Enumeration of circulating tumor cells (CTC) is prognostic in patients with MBC^4,5^. We hypothesized that in addition to enumerating CTC, CTC phenotyping might provide biologic insight into their behavior. We have previously developed an analytically validated assay, designated the CTC-endocrine therapy index (CTC-ETI), which is based on CTC enumeration, as well as semiquantitative analyses of CTC expression of four different markers associated with ET benefit [ER, B-cell lymphoma 2 (BCL2)] or resistance [human epidermal growth factor receptor (HER2) and Ki67]^6^. We further hypothesized that CTC-ETI could identify a subset of patients with ER-positive, HER2-negative MBC who might have ET-refractory disease and would be better treated with chemotherapy.

We report the results of the prospective, multi-institutional COMETI trial (P2-2012.0; NCT01701050), which was designed to test this hypothesis.

## Results

### Patient cohort

**Fig. 1:**
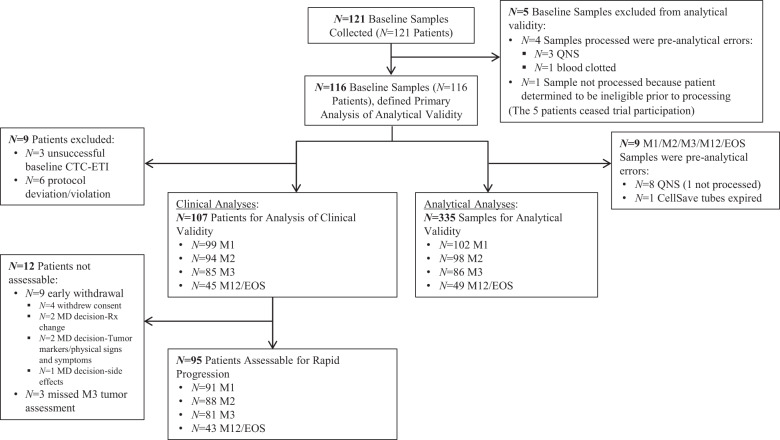
REMARK diagram for patient enrollment, exclusions, and disposition. QNS quantity not sufficient, M1 month 1, M2 month 2, M3 month 3, M12 month 12, EOS end of study.

Between April 2013 and November 2015, pretreatment (baseline) samples were collected from 121 patients enrolled at 19 centers in North America (Fig. 1). According to protocol, five patients’ samples were excluded because of pre-analytical errors (*N* = 4) or patient ineligibility (*N* = 1) determined prior to processing. Those five patients ended study participation. Samples were also collected from patients at four subsequent time points [month 1 (M1), 2 (M2), 3 (M3), and 12 (M12) or progression (end of study, EOS)]. In the 116 baseline samples and 335 samples from subsequent time points, CTC-ETI was successfully ascertained according to the protocol definition in 91 and 95% of samples, exceeding our pretrial stipulation for analytical success (see Supplementary Materials). An additional nine patients were excluded from the clinical analyses: three because of unsuccessful baseline CTC-ETI and six for protocol deviation/violation.

The one-hundred seven patients included in the analyses of clinical validity (Table 1) had a median age of 63 (range 33–84). All patients had ER-positive breast cancer, either in their primary or metastatic tumors. Of note, one patient had an ER and PgR negative, and an additional seven patients had ER and PgR unknown primary cancers. Each of these eight patients had ER-positive metastatic biopsies.

**Table 1:** Patient and tumor characteristics at baseline.

Characteristic		Overall *N* (%)^a^	≥5 CTC/7.5 ml WB *N* (% of subgroup)	*P* value^b^
Number of patients		107	38 (36%)	
Primary HR status				0.19
ER positive	PgR positive	86 (80%)	32 (37%)	
	PgR negative	10 (9%)	6 (60%)	
	PgR unknown	2 (2%)		
ER negative	PgR positive	1 (1%)	NA	
	PgR negative^c^	1 (1%)	NA	
	PgR unknown^c^	7 (7%)	NA	
Metastatic HR status				—
ER positive	PgR positive	46 (43%)	12 (26%)	
	PgR negative	19 (18%)	6 (32%)	
	PgR unknown	5 (5%)	3 (60%)	
ER unknown	PgR unknown	37 (35%)	17 (46%)	
Endocrine resistance status at the time of study enrollment				1.00
Primary ET resistance^d^		12 (11%)	4 (33%)	
Secondary (acquired) ET resistance^e^		95 (89%)	34 (36%)	
Prior adjuvant/extended adjuvant ET (%)		68 (64%)	22 (32%)	0.41
No. of previous ET MBC lines (%)				0.28
0		25 (23%)	13 (52%)	
1		50 (47%)	15 (30%)	
2		21 (20%)	6 (29%)	
≥3		11 (10%)	4 (36%)	
Sites of disease				0.08
Bone only		32 (30%)	9 (28%)	
Lymph node/soft tissue ± bone		17 (16%)	3 (18%)	
Visceral ± other		58 (54%)	26 (45%)	
RECIST target lesions				
Yes		59 (55%)	23 (39%)	
No		48 (45%)	15 (31%)	
RECIST nontarget lesions				
Yes		100 (93%)	37 (37%)	
No		7 (7%)	1 (14%)	
ECOG performance status (%)				0.04
0		52 (49%)	13 (25%)	
1		46 (43%)	22 (48%)	
2		6 (6%)	1 (17%)	
Unknown		3 (3%)	2 (67%)	

^a^All values were rounded, exact percentages total 100%.

^b^The *P* value was a test of independence between the variables in the rows and CTC dichotomy (<5 vs. ≥5 CTC).

^c^One patient had ER/PgR negative, and seven patients had unknown ER and PgR status of the primary tumor, but in each case metastatic tissue was ER positive.

^d^Defined as a relapse while on the first 2 years of adjuvant ET, or PD within first 6 months of first line ET for MBC, while on ET.

^e^Defined as relapse while on adjuvant ET, but after the first 2 years, or relapse within 12 months of completing adjuvant ET, or PD ≥ 6 months after initiating ET for MBC, while on ET.

Of the 107 eligible patients, 25 (23%) had developed metastases while on or within 12 months of completing adjuvant ET, and 50 (47%), 21 (20%), and 11 (10%) had received 1, 2, or ≥3 lines of ET for MBC, respectively. CTC enumeration did not significantly differ among these groups (Table 1). According to the advanced breast cancer 3 (ABC 3) definition^7^, at the time of the study enrollment, 12 (11%) and 95 (89%) of the 107 patients had primary endocrine resistance or secondary, acquired endocrine resistance, respectively. Thirty-two (30%) patients had bone lesions only; and 59 (55%) had a measurable disease according to Response Evaluation Criteria in Solid Tumors (RECIST) criteria. During the trial, 54 (51%), 42 (39%), 12 (11%), and 2 (2%) patients were treated with fulvestrant alone or in combination, aromatase inhibitors alone or in combination, tamoxifen, or another ET, respectively, and 26 (24%) were treated with ET plus either palbociclib (*N* = 12) or everolimus (*N* = 14).

### Progression-free survival (PFS)

**Fig. 2:**
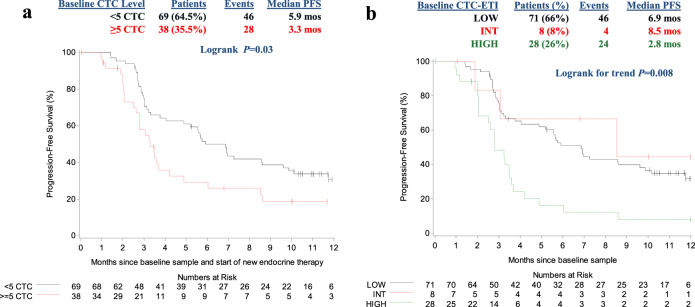
Progression-free survival (PFS) according to baseline CTC enumeration and CTC-ETI. **a** CTC enumeration levels: (solid black line, <5 CTC/7.5 ml whole blood; dashed red line, ≥5 CTC/7.5 ml whole blood). **b** CTC-ETI: (solid black line, CTC-ETI low; short dashed red line, CTC-ETI intermediate; long dashed green line, CTC-ETI high).

At baseline, CTC were elevated (≥5 CTC/7.5 ml whole blood (WB)) in 38/107 (36%) patients (Tables 1 and 2). Patients with ≥5 vs. <5 CTC/7.5 ml WB had significantly worse PFS (median PFS 3.3 vs. 5.9 months; logrank *P* = 0.03; Fig. 2a).

**Table 2:** Distribution of CTC-ETI at baseline and subsequent time points.

		CTC-ETI				
		Low^b^ (score 0–3)		Int (score 4–6)	High (score 7–16)	
	Elevated CTC at time point (% of total)^a^	CTC/7.5 ml WB				Not determined
		<5	≥5	≥5	≥5	
CTC-ETI at baseline						
Patients (% due to high or low CTC)	38 (36%)	69 (97%)	2 (3%)	8	28	0 (0%)
Total *N* (% of total)	107	71 (66%)		8 (8%)	28 (26%)	0 (0%)
CTC-ETI at month 1						
Patients (% due to high or low CTC)	24 (24%)	75 (100%)	0 (0%)	6	18	0 (0%)
Total *N* (% of total)	99	75 (76%)		6 (6%)	18 (18%)	0 (0%)
CTC-ETI at month 2						
Patients (% due to high or low CTC)	20 (21%)	73 (99%)	1 (1%)	1	18	1 (100%)
Total *N* (% of total)	94	74 (79%)		1 (1%)	18 (19%)	1 (1%)
CTC-ETI at month 3						
Patients (% due to high or low CTC)	15 (18%)	69 (99%)	1 (1%)	1	13	1 (100%)
Total *N* (% of total)	85	70 (83%)		1 (1%)	1 (15%)	1 (1%)

^a^Average CTC/7.5 ml whole blood (WB) among the four aliquots used for CTC-phenotyping.

^b^CTC-ETI low can either be due to low number of CTC (<5/7.5 ml WB) to perform the assay or sufficient number (≥5/7.5 ml WB) to perform assay, but with low CTC-ETI score.

At baseline, CTC-ETI low (score ranged 0–3) was observed in 71/107 (66%) patients (Table 2). However, low CTC-ETI occurred almost exclusively due to low CTC [<5/7.5 ml WB; *N* = 69/71 (97%) patients], rather than high CTC levels with favorable biology [≥5 CTC/7.5 ml WB with high ER and BCL2, and/or low HER2 and Ki67 high; *N* = 2/71 (3%) patients]. Eight percentage and 26% of the 107 patients had CTC-ETI intermediate (score ranged 4–6) and high (score ranged 7–16), respectively (Table 2). Of the 38 patients who had ≥5 CTC/7.5 ml WB, 8 (21%) and 28 (74%) had CTC-ETI intermediate (score ranged 4–6) and high (score ranged 7–16), respectively.

High, compared to low or intermediate, CTC-ETI at baseline was associated with worse PFS (median PFS for low CTC-ETI, intermediate CTC-ETI, and high CTC-ETI = 6.9, 8.5, and 2.8 months, respectively; logrank for trend *P* = 0.008; Fig. 2b).

**Fig. 3:**
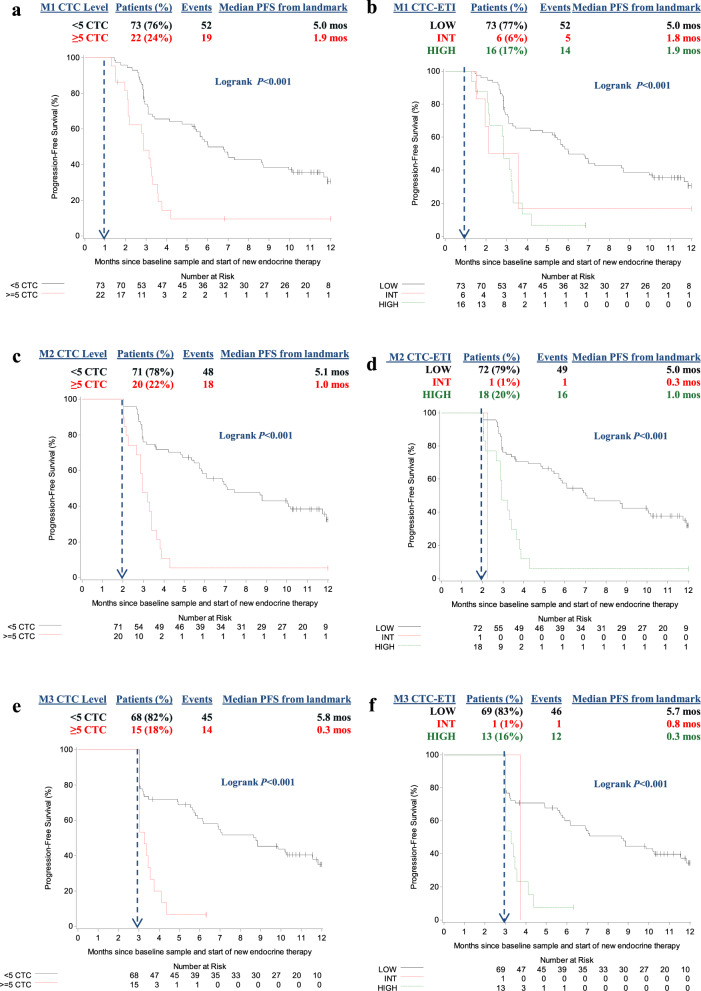
Progression-free survival (PFS) according to CTC levels and CTC-ETI determined at follow-up time points. CTC enumeration levels: solid black line, <5 CTC/7.5 ml whole blood; dashed red line, ≥5 CTC/7.5 ml whole blood. CTC-ETI: solid black line, CTC-ETI low; short dashed red line, CTC-ETI intermediate; long dashed green line, CTC-ETI high. **a**, **b** At month 1 (M1). *N* = 99 pts with M1 sample, 95 pts were analyzed in landmark analysis of PFS (*N* = 4 PFS censored on D1, no reimaging). **a** CTC enumeration. **b** CTC-ETI. **c**, **d** At month 2 (M2). *N* = 94 pts with M2 sample, 91 pts were analyzed in a landmark analysis of PFS (*N* = 2 PFS censored on D1, no reimaging; *N* = 1 no CTC count). **c** CTC enumeration. **d** CTC-ETI. **e**, **f** At month 3 (M3). *N* = 85 pts with M3 sample, 83 pts were analyzed in a landmark analysis of PFS (*N* = 1 PFS censored on D1, no reimaging; *N* = 1 no CTC count). **e** CTC enumeration. **f** CTC-ETI. For **a**–**f**: blue vertical dashed line indicates sample time point landmark; median PFS is calculated from this landmark.

CTC were elevated (≥5 CTC/7.5 ml WB) in 24/99 (24%), 20/94 (21%), 15/85 (18%), and 7/45 (16%) samples collected at M1, M2, M3, and M12/EOS (Table 2), respectively. Patients who had ≥5 vs. <5 CTC/7.5 ml WB at each of these time points had significantly worse PFS (Fig. 3a, c, e). Indeed, median PFS for patients with elevated CTC at M1, M2, and M3 after starting study ET were 1.9, 1.0, and 0.3 months from the time of sample collection, respectively, compared to PFS for patients with low CTC levels (median PFS 5.0, 5.1, and 5.8 months, respectively). At 1-month follow-up, only a single patient had elevated CTC levels that increased from low CTC levels at baseline. Therefore, elevated CTC at M1 almost entirely represented persistently elevated CTC from baseline, whereas low CTC levels included those with either persistently low or decline in CTC levels during therapy. This finding was less true at M2 and M3, as cumulatively three and five patients had elevated CTC levels that increased from low CTC levels at baseline, respectively (Supplementary Fig. 1a–c). All five of these patients with increased CTC levels had progression at their first reimaging.

Distributions of CTC-ETI in samples collected at M1, M2, M3, and M12/EOS are illustrated in Table 2. At M1, high or intermediate vs. low CTC-ETI was associated with worse PFS (median PFS from sample collection for high, intermediate, and low CTC-ETI = 1.9, 1.8, and 5.0 months, respectively; *P* < 0.001; Fig. 3b). Likewise, high CTC-ETI at M2 and M3 were associated with worse PFS compared to low CTC-ETI (Fig. 3d, f). Very few patients had intermediate CTC-ETI at these subsequent time points.

### Rapid progression (RP)

**Fig. 4:**
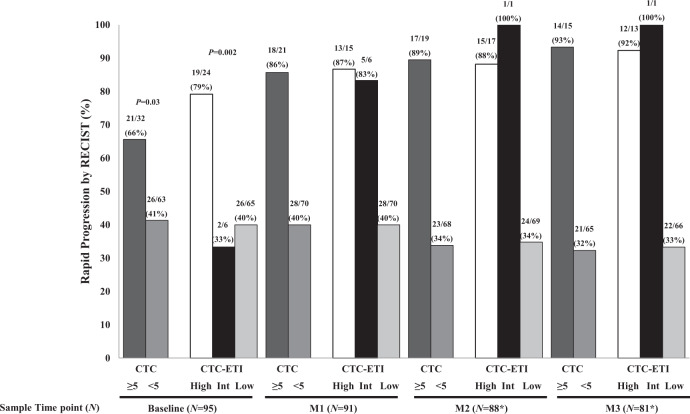
Association of CTC enumeration and CTC-ETI at baseline, and month 1 (M1), month 2 (M2), and month 3 (M3) with rapid progression. CTC enumeration:  ≥5;  <5 CTC/7.5 ml whole blood. CTC-ETI:  CTC-ETI high;  CTC-ETI intermediate;  CTC-ETI low. *One sample at M2 and one sample at M3 did not have assessable CTC level or CTC-ETI.

Of the 107 patients included in the clinical analyses, 95 were assessable for, and 47 [50% (95% CI 39–60%)] had, RP (Fig. 1). Patients with ≥5 vs. <5 CTC/7.5 ml WB at baseline significantly more frequently experienced RP [66% (95% CI 47–81%) vs. 41% (95% CI 29–54%); *P* = 0.03] (Fig. 4).

Elevated CTC-ETI was also associated with RP (*P* = 0.002). In particular, 19/24 (79%; 95% CI, 58–93%) patients with high vs. 26/65 (40%; 95% CI 28–53%) with low CTC-ETI experienced RP (Fig. 4). In the small group with intermediate CTC-ETI, two of six patients (33%) experienced RP. However, only two patients with CTC ≥ 5 had low CTC-ETI, preventing analysis of whether CTC-ETI contributed to the estimate of RP compared to CTC enumeration alone (Fig. 4).

For the 95 patients, 91/91, 87/88, and 80/81 samples were assessable for CTC at M1, M2, and M3, respectively. Patients with ≥5 vs. <5 CTC/7.5 ml WB at M1 experienced higher rate of RP (86% vs. 40%; Fig. 4). Likewise, similar results were obtained for M2 (89% vs. 34%) and M3 (93% vs. 32%; Fig. 4). CTC-ETI at M1 also was associated with RP. The rate of RP for patients with high CTC-ETI was 87% (13/15; 95% CI 60–98%) vs. 83% with intermediate (5/6; 95% CI 36–100%) and 40% (28/70; 95% CI 29–52%) for those with low CTC-ETI (Fig. 4). Similar results were found with CTC-ETI in the M2 and M3 samples (Fig. 4).

### Retraining of CTC-ETI

The CTC-ETI algorithm was initially constructed based on assumptions of ET sensitivity and resistance from prior investigations^6^. Since the contributions of CTC-ETI as prospectively evaluated, compared to CTC enumeration alone, were disappointing in this study, we proceeded to conduct exploratory analyses to determine if a redesigned CTC-ETI algorithm might have more clinical importance. The small number of patients having ≥5 CTC/7.5 ml WB at baseline limited the ability of these analyses to detect meaningful relationships of the CTC phenotypes with clinical outcomes. Nonetheless, focusing on patients with ≥5 CTC/7.5 ml WB at baseline, the percentages of CTC that were positive for each of the four markers did not improve upon CTC enumeration in exploratory modeling (Supplementary Fig. 2). The absence of relationship of the prespecified CTC-ER phenotype with PFS was consistent with the modeling (Supplementary Fig. 3).

## Discussion

In this prospective, multi-institutional, and multi-national phase 2 trial, we investigated the prognostic effect of a phenotypically based CTC assay, the CTC-ETI, in patients with ER-positive, HER2-negative MBC starting second-line or later ET^6^. As expected^4,5,8–10^, we observed that prognosis of patients with MBC who had elevated CTC at baseline was worse than those who did not. However, the results did not support our original hypothesis that CTC-ETI, which is an empirically derived weighted algorithm based on the assumed relative contributions of CTC-ER, BCL2, HER2, and Ki67 expression, would be superior to CTC enumeration alone in identifying patients with ER-positive, HER2-negative MBC who would not benefit from ET.

Nonetheless, the COMETI trial has confirmed that failure to reduce CTC to <5/7.5 ml WB within the first or second month of systemic antineoplastic therapy, specifically ET, for patients with ER-positive MBC is associated with rapid subsequent progression of disease, and that perhaps such patients are refractory to ET alone. Failure to reduce CTC within 1 month of therapy in patients with MBC has been reported in several prior studies, most of which included patients with a variety of intrinsic subtypes and treated with various therapies^9^. Indeed, in the SWOG S0500 trial, patients with MBC starting first-line chemotherapy and who had elevated CTC at baseline, but failed to reduce them to <5/7.5 ml WB had very poor prognosis. The median OS of these patients was ~13 months, and 75% died within 18 months^5^. Similar findings were reported in a retrospective analysis of serial CTC in CALGB 40502 (ref. ^11^). The COMETI trial only included patients with ER-positive, HER2-negative MBC who were starting a new ET, and 86% of patients whose CTC were >5/7.5 ml WB at first 1-month follow-up experienced RP on the ET regimen initiated by their oncologist. Therefore, as established for first-line chemotherapy in the S0500 trial, our data indicate that failure to experience a CTC response provides an early indication of lack of benefit from ET in ER-positive MBC. However, CTC-ETI at later time points did not appear to add to CTC enumeration for prediction of either PFS or RP.

Exploratory efforts to “retrain” the CTC-ETI, using the clinical outcomes data generated in this trial, were unsuccessful in identifying either a single CTC-biomarker or a combined CTC-biomarker signature that provided more prognostic information than CTC enumeration alone. Specifically, CTC-ER, which we “overweighted” in our original CTC-ETI algorithm based on its importance as the target of ET, did not contribute to CTC levels alone.

Recently reported results of the multicenter phase III STIC CTC trial^12^ are consistent with our data that CTC enumeration is a prognostic marker in patients with ER-positive, HER2-negative MBC receiving ET. In the STIC CTC trial, patients with newly diagnosed metastases were randomly assigned to using CTC or according to physician’s choice (without knowledge of CTC) for selection of ET or chemotherapy as first-line therapy. In this trial, in which only CTC enumeration, but not phenotyping, was determined, PFS was slightly longer for the CTC arm, with a HR for progression of 0.94 (90% C.I. 0.81–1.09). The investigators concluded that baseline CTC might be useful to drive palliative ET or chemotherapy choice as first-line therapy in ER-positive, HER2-negative MBC. Serial CTC were not obtained, so the potential benefit of early identification of ET refractory disease in patients with ER-positive MBC could not be ascertained.

The COMETI study has several strengths. One primary objective was to determine if CTC-ETI could be successfully performed in central laboratories to which blood specimens from patients in multiple institutions in North America were submitted, which was achieved. Further, accrual eligibility and outcomes were prospectively dictated and determined by protocol with clinical trial quality. However, treatment recommendations were left to the patients’ oncologists, making this study more generalizable to clinical practice. The COMETI trial did not include randomization, to specifically address the clinical utility of CTC at baseline or later time points in patients with ER-positive MBC. Further, the data demonstrating the benefit of CDK4/6 inhibitors when added to ET only came into consideration in the latter portion of accrual^13^, so we were unable to determine what effect CTC, or CTC-ETI, might have on outcomes in patients treated with this combination. A limitation of this work is the lack of circulating tumor DNA (ctDNA) data. Previous studies have reported that activating mutations in the ligand-binding domain of the gene encoding for ER (*ESR1*) assessed in ctDNA are associated with worse prognosis in ER-positive, HER2-negative MBC^14,15^. Adequate plasma specimens for ctDNA analysis were not collected in the COMETI study.

In summary, CTC enumeration at initiation of ET, and at early and following time points was prognostic in ER-positive MBC starting second-line or later ET. Although we demonstrated that the phenotypic-based CTC-ETI algorithm could be accurately determined in a multi-institutional, multi-national, prospective study, the results of the COMETI trial failed to demonstrate that it adds to enumeration alone, at baseline or during serial follow-up time points^6,16^. Further research is necessary to identify patients with ER-positive MBC who, like patients with ER-negative MBC, will not benefit from ET and would be better treated with either chemotherapy or other targeted systemic therapies.

## Methods

### Study design

Patients with ER-positive, HER2-negative (based on ASCO/CAP criteria)^17^, and progressive MBC after one or more lines of ET or, who developed metastases during or within 12 months of completing adjuvant ET, and who were initiating a new ET were enrolled onto a prospective multi-institutional phase 2 trial. Patients must have had an ECOG performance status of 0–2 (ref. ^18^) and either measurable or nonmeasurable, but evaluable MBC according to RECIST v1.1 (ref. ^19^) with at least one nonirradiated distant site of metastasis. Patients who were progressing within the 120-day wash-out period of fulvestrant (three half-life times of the drug), or those with brain metastases only, were not eligible for the study. The choice of ET was at the discretion of the treating physician and included, but was not limited to, surgical ovariectomy, tamoxifen, LH–RH agonists, aromatase inhibitors, fulvestrant, megestrol acetate, or pharmacologic doses of estrogen. The combination of ET with everolimus was allowed and after the FDA approval of palbociclib, an amendment permitted the combination of it with ET. No investigational drugs were allowed. The study was approved by the Institutional Review Board of each participating center, and all the enrolled patients provided written informed consent in accordance with the Declaration of Helsinki. The full protocol is provided in Supplementary Material.

All patients were staged at baseline (within 30 days of beginning treatment) with body imaging [computed tomography (CT) scans of chest/abdomen/pelvis alone or PET/CT alone]. If the CT scans provided sufficient evaluation of bone metastases, inclusion of a standard bone scan was optional. Clinical follow-up and imaging studies were repeated 3 months after the initiation of therapy (within ±14 days) and when the patient was taken off study (i.e., maximum of 12 months after the initiation of therapy, at the time of disease progression, or at the time of discontinuation of treatment, whichever occurred first), using the same imaging modalities employed at baseline to assess the selected target and/or nontarget lesions according to RECIST v1.1 guidelines. Other imaging performed during the course of the study was at the discretion of the managing physician (Supplementary Fig. 4).

### Specimen collection

Prior to starting a new ET treatment (baseline, BL) and at four subsequent time points [month 1 (M1), 2 (M2), 3 (M3), and 12 (M12) or progression] all patients had ~40 ml WB drawn into five CellSave tubes. These tubes were pooled and divided into four different 7.5 ml aliquots for CTC enumeration and characterization using the CellSearch® system, as previously described^6^. Collectively, these four aliquots were considered one sample for each time point. CTC and CTC-ETI were determined at BL, M1, M2, M3, and M12 (EOS) or at the time of progression (M12/EOS), whichever occurred first (Supplementary Fig. 4). Specimens from the first 32 patients were sent to the Breast Oncology Laboratory at the University of Michigan (UM). Subsequently, samples were either sent to UM or to a second laboratory at the Mayo Clinic, to provide assessment of the ability to calculate CTC-ETI in separate laboratories. CellSearch images obtained at Mayo Clinic were subsequently reread, without knowledge of prior scoring, by the UM lab. Because the concordance between the two laboratories was slightly lower than expected compared to the concordance within UM, the UM scoring was used in all analyses of clinical validity.

### Isolation, enumeration, and characterization of CTC

CTC were isolated, enumerated, and phenotyped for ER, BCL2, HER2, and Ki67 using the CellSearch® CXC Kit and CellSearch® system, according to manufacturer’s instructions (Menarini Silicon Biosystems, Inc., Huntingdon Valley, PA)^4,6^. In the CXC Kit, three fluorescent channels were used to distinguish CTC from WBC using DAPI, anti-cytokeratin, and anti-CD45. The fourth channel was used to measure expression of each biomarker in separate aliquots of the pooled WB specimens: ER (10 µg/µl; monoclonal murine ER-119.3; Menarini Silicon Biosystems, Inc.), BCL2 (1 µg/µl; monoclonal murine BCL-2/ (100); BD Pharmingen Cat 340576), HER2 (8 µg/µl; monoclonal murine Her81; Menarini Silicon Biosystems, Inc.), and Ki67 (0.625 µg/µl; monoclonal murine B56; BD Pharmingen Cat 556027), as previously described^6^.

### Successful CTC-ETI determination

Pre-analytical errors as well as unresolved technical failure (reagents and instrument failure, unsatisfactory sample quality and results due to interfering substances or inability to interpret marker results, and analytical failure), and laboratory failures were prespecified in the protocol. Considerations for calculation of CTC-ETI, as well as rules to round the average enumeration were all prespecified in the protocol (see “Protocol” in Supplementary Material).

### CTC-ETI determination and algorithm (modified from previously reported method^6^)

To generate CTC-ETI, assumptions were made about the relative prognostic and predictive effects of each of the biomarkers, including the use of a CTC cutpoint of ≥5/7.5 ml WB, as previously described^6^. CTC-ETI was considered low if a patient had <5 CTC/7.5 ml WB.

CTC levels were enumerated in each of the four different aliquots of 7.5 ml WB. The average CTC count of the four tubes was used to assign the CTC-enumeration points for that blood draw using modified CTC-positive categories as follows: 0 points = average <5 CTC/7.5 ml WB (favorable outcome), 1 point = average 5–10 CTC/7.5 ml WB, 3 points = average 11–100 CTC/7.5 ml WB (intermediate outcome), and 4 points = average >100 CTC/7.5 ml WB (worst outcome) (Supplementary Table 1a). In the original CTC-ETI algorithm, there were only three CTC-enumeration point categories. The modified CTC-ETI algorithm separated the previously highest CTC-enumeration point category (>10 CTC) into 10–100 and >100 CTC categories, which now account for 3 and 4 CTC-enumeration points, respectively, increasing the maximum CTC-ETI score from 14 to 16 (see Supplementary Table 1).

If the average CTC/aliquot was ≥5/7.5 ml WB, CTC-Bio-Points were determined for each marker, based on the percentage of CTC that were positive (2+ or 3+) for the respective marker. Arbitrarily, we established three categories of positive staining: 0%, 1–10%, and >10% of CTC staining for each marker (Supplementary Table 1b), as previously reported. Positive CTC-ER and BCL2 readings were given low points (=sensitivity to ET), while positive CTC-HER2 and Ki67 readings were given high points (=resistance to ET; Supplementary Table 1b). The sum of assigned CTC-Bio-Points for each marker produces a final CTC-Bio-Score.

The CTC-enumeration points were combined with the CTC-Bio-Score to derive the final CTC-ETI score (Supplementary Table 1c), according to the following equation:

CTC-ETI = [CTC-enumeration points] + [Bio-Score].

Thus, CTC can range from 0 to 16 (Supplementary Table 1d). To make the CTC-ETI score clinically applicable, the scores were placed into three categories, much as histologic grading is calculated: low CTC-ETI score = 0–3, intermediate CTC-ETI score = 4–6, and high CTC-ETI score = 7–16.

CTC-ETI was determined by two independent operators at UM (K.A., E.M.D., E.P.D., and C.P.). Discordant results were reconciled by joint readings. No CTC-enumeration, CTC-biomarker, or CTC-ETI results were provided to patients or their caregivers, and all analyses were conducted without knowledge of the patients’ treatment or clinical status or other CTC results.

### Analytical validity

The CTC-ETI assay using WB specimens was developed at a single institution (UM)^6^. One of the objectives of the COMETI trial was to determine if CTC-ETI could be successfully performed in WB specimens drawn at multiple institutions and mailed to a central reference laboratory. To demonstrate that the CTC-ETI can be accurately determined at baseline in patients from multiple centers across North America, the baseline blood sample was evaluated for successful calculation of CTC-ETI, including successful enumeration of CTC in the four aliquots and successful determination of CTC Bio-Score for all four markers when the average number of cells is ≥5 CTC/7.5 ml WB. There were two independent, sequential analyses of analytical validity planned, the first after ~35–40 patients were enrolled and the second after ~70–80 patients were enrolled. Both analyses were based on the binary endpoint of successfully calculating a CTC-ETI in the baseline sample. For each analysis, we desired ≥80% success, whereas ≤60% success was considered as too low. If the number of successes was ≥24/32 then the null hypothesis was rejected with *α* = 0.057 (target *α* = 0.06); if the number of successes was ≤23/32 then the alternative hypothesis was rejected with *β* = 0.175 (target *β* = 0.20). All patients for whom the baseline blood sample was collected and shipped to the study laboratory without handling/pre-analytical errors would be considered evaluable for the purposes of analytical validity analyses. During trial conduct, there was at least one occasion when the sample was shipped but the patient was determined to be ineligible before the sample arrived at the laboratory, and it was decided that the sample should not be assessed and should be excluded from all analyses, and documented in the study flow diagram.

Samples were initially all sent to the UM Breast Oncology Laboratory to evaluate analytical validity associated with assessing baseline CTC-ETI in blood collected at multiple clinical sites and processed in one study laboratory. Once the baseline CTC-ETI was attempted in the first 32 evaluable patients (approximately the first 35–40 enrolled patients) in the single study laboratory, the first analysis took place.

Subsequently samples were sent to two study laboratories to evaluate the analytical validity associated with assessing baseline CTC-ETI collected at multiple clinical sites and assayed in multiple study laboratories. Once the baseline CTC-ETI was attempted in the next 32 evaluable patients (approximately the next 35 enrolled patients, or a total of approximately 70–80 enrolled patients) at two study laboratories, the second analysis took place in the subsequent 32 evaluable patients, in the same manner as the first (see Supplementary Material for greater detail and protocol for analytical plan).

### Statistical analysis

The clinical validity of the CTC-ETI was assessed by its association with PFS and RP. PFS was measured as the time from the date of baseline sample until the date of first documentation of progressive disease according to RECIST v1.1 criteria, or death due to any cause. In absence of these events, PFS was censored at the date of the last objective assessment (up to a maximum of 12 months after the initiation of ET). RP was defined as the presence or absence of objective radiographic progression according to RECIST v1.1 criteria or death due to MBC within 3 months. Patients without reimaging at 3 months to determine RP status were omitted from the analysis, even in the situation of symptomatic deterioration or rising serum tumor markers (i.e., CA 15-3/27.29 or CEA).

The distribution of PFS was estimated using Kaplan–Meier method. The associations of elevated CTC (≥5 CTC/7.5 ml WB) and of CTC-ETI categories with PFS were assessed, using logrank test and test for trend (2 degrees of freedom), respectively. For post-baseline samples, a landmark analysis approach was used^20^ in which PFS was redefined from the date of the relevant sample among the subset of patients who had a sample and were progression free at the sample time point. Changes in CTC were analyzed similarly, with categories defined as: increase (baseline < 5, M ≥ 5 CTC/7.5 ml WB), decrease (baseline ≥ 5, M < 5 CTC/7.5 ml WB), low (baseline < 5, M < 5 CTC/7.5 ml WB), and high (baseline ≥ 5, M ≥ 5 CTC/7.5 ml WB). The associations with RP were assessed using Fisher’s exact test and test for trend.

The statistical design anticipated a total of 120 patients would be required to obtain at least 51 (42%) RP events and 85 PFS events for analysis after the last enrolled patient reached 3 months follow-up, with maximum 12 months follow-up. With assumptions that 30%, 40%, and 30% of patients would have low, intermediate, and high CTC-ETI, respectively, and 20%, 34%, and 75% of patients would experience RP, respectively, there was >90% power on the basis of a Fisher’s exact test and a logrank test (each two-sided *α* = 0.05).

### Retraining CTC-ETI

Exploratory analyses investigated the contributions of CTC phenotypes (percentage of CTC positive for the respective marker) to CTC enumeration in relation to RP and aimed to retrain the CTC-ETI. A feature of the data is that biomarker expression values were analyzed only if the CTC count is at least 5 CTC/7.5 ml WB. This introduces the challenge of combining patients with low CTC counts and high CTC counts into the model. For example, it did not seem appropriate to equate 100% ER if 1/1 CTC was ER positive and 100% ER if 100/100 CTC were ER positive. Different approaches of weighting to compensate for this issue led to a weighted variable as an expression of CTC count rather than biomarker percentage, which was the real interest in the analysis. To focus in on the marker expression, only the 32 patients that were eligible from the RP analysis and had an average CTC count of ≥5 CTC/7.5 ml WB were included in the modeling. Among the 32 eligible patients, 21 (66%) experienced RP. The data were split into training (*N* = 22) and validation sets (*N* = 10). To compensate for the small sample size, sampling with replacement was used on the training dataset to increase the number of observations to 500. This process was repeated 500 times creating 500 unique pairs of validation and training sets.

To model the relationship between average CTC count and the biomarkers both random forests and logistic regression were used. Random forest was the chosen method due to its flexible nature and its lack of underlying assumptions and logistic regression as an additional method to act as a point of comparison. Each tree in the random forest was built using the variables: average CTC count (from the four aliquots in which marker immunostaining was determined), percentage ER, percentage BCL2, percentage HER2, and percentage Ki67. In total, 500 trees were modeled using each of the 500 training datasets once. For every training dataset that built a tree, its matching validation data set was then used to calculate predicted probabilities. The predicted probabilities from all validation datasets were then combined, and the mean predicted probability for all patients across all validation datasets was calculated. These validated probabilities were then used to estimate a receiver operating characteristic (ROC) curve and accompanying area under the ROC curve (AUC) statistic. The trees also generated measures of variable importance, and the most important variable in each tree was recorded.

Each logistic model utilized the same variables as the random forest. In a similar manner, models were built on each training dataset, and predicted probabilities were calculated applying the validation dataset to their respective model. The means of the predicted probabilities for each patient was then calculated and used to estimate a ROC curve and accompanying AUC statistic.

Biomarkers variables that were found to be consistently most important in the tree building process were then evaluated, using univariate logistic models to characterize the relationship (see Supplementary Material for greater detail).

The study is reported according to the REMARK guidelines^21^.

### Reporting summary

Further information on research design is available in the Nature Research Reporting Summary linked to this article.

## Supplementary information

Supplementary Information

Reporting Summary

## Data Availability

The data generated and analyzed during this study are described in the following data record: 10.6084/m9.figshare.14473242 (ref. ^22^). The clinical outcomes data are not publicly available for the following reason: data contain information that could compromise research participant privacy. The CTC enumeration data/time point data and the CTC-endocine therapy index data have not been made openly available, but they are available upon request to the corresponding author.

## Code availability

The code for data processing and visualization is written in SAS v8 and v9 (SAS Institute, Cary, NC) and available upon reasonable request.
