# Supplementary material for: Circulating tumor cell number and endocrine therapy index in ER positive metastatic breast cancer patients
Source: NPJ Breast Cancer. 2021 Jun 11;7:77. doi: 10.1038/s41523-021-00281-1 (PMC8196036; doi:10.1038/s41523-021-00281-1)
Supplement: Supplementary file 1 — Supplementary Information [file 41523_2021_281_MOESM1_ESM.pdf]

**SUPPLEMENTARY MATERIAL**

**Circulating tumor cell number and endocrine therapy index in ER positive metastatic breast cancer patients**

Costanza Paoletti, Meredith M. Regan, Samuel M. Niman, Emily M. Dolce, Elizabeth P. Darga, Minetta C. Liu, P. Kelly Marcom, Lowell L. Hart, John W. Smith II, Karen L. Tedesco, Eitan Amir, Ian E. Krop, Angela M. DeMichele, Pamela J. Goodwin, Margaret Block, Kimberly Aung, Martha E. Brown, Robert T. McCormack, Daniel F. Hayes

## SUPPLEMENTARY RESULTS

### Analytic validity of CTC-ETI

Between April 2013 to November 2015, baseline samples were collected from 121 patients enrolled at 19 centers in North America (**Figure 1**). Five samples were excluded because of pre-analytical errors (*N*=4) or patient ineligibility (*N*=1) and those five patients ended study participation. Therefore, 116 baseline samples defined the primary population for assessing analytical validity. An additional 344 post-baseline samples were collected, of which 335 contributed to assessment of analytical validity. The sample collection compliance was 97%.

### Success of CTC-ETI in specimens collected in multiple centers

CTC-ETI was successfully determined in 106/116 (91%; 90% CI, 86% to 95%) baseline samples according to pre-determined protocol criteria, meeting the first primary objective of the trial. Reasons for unsuccessful determination of CTC-ETI included delayed processing (≥96 hours) after collection (*N*=1), or had unresolved technical failures (*N*=9; 3 samples had unsatisfactory results [due to interfering substances or inability to interpret marker results], 4 analytic failures, 2 analytic and reagent/instrument failures).

Of note, seven of the patients were classified as analytically unsuccessful according to the protocol definition because one of the four WB aliquots had a high CTC count outlier (i.e., one ≥5 and others <5). However, these seven patients had adequate information to calculate CTC-ETI and were therefore included in clinical analyses.

CTC-ETI was also successfully determined in 317/335 (95%; 95% CI, 92% to 97%) post-baseline samples, ranging from 98% to 88% across the 4 time points. CTC-ETI could not be determined in 18 samples for the following reasons: 1. delayed processing ≥96 hours after collection (N=1 sample); 2. unresolved technical failures [*N*=17 samples: unsatisfactory results

(*N*=8); analytic failures (*N*=5), reagent/instrument failure (*N*=1), unsatisfactory results and analytic failure (*N*=1); analytic and reagent/instrument failures (*N*=1); sample had insufficient blood in one aliquot (*N*=1)].

Taken together, these data confirm that CTC-ETI was performed satisfactorily at baseline and subsequent time points in a multi-institutional, multi-national clinical trial according to predetermined, protocol-stipulated criteria.

**Concordance of Multiple CTC-ETI Readings**

Twelve samples originally read at UM, all with ≥5 CTC/7.5 ml WB, were randomly selected and reread at UM without knowledge of prior scoring. Strong agreement in assessing CTC-ETI was found between two readings at UM laboratory. In particular, among these samples, 12/12 (100%) were concordant for CTC-ETI category (**Supplementary Table 2a**) and 9/12 (75%) were concordant for CTC-ETI score (**Supplementary Table 2b**).

One hundred-three samples were received and analyzed by Mayo Clinic laboratory and reread by UM laboratory without knowledge of prior scoring. Ninety-three (90%) were concordant for CTC-ETI category between the two laboratories (**Supplementary Table 3a**) and 66/103 (64%) were concordant for CTC-ETI score (**Supplementary Table 3b**). Of the 10 discordant samples between CTC-ETI categories, four samples moved from CTC-ETI high to intermediate, four samples moved from CTC-ETI low to “unable to be successfully determined” category, one sample moved from “unable to be successfully determined” category to CTC-ETI high, and one sample moved from “unable to be successfully determined” to CTC-ETI low.

Overall, 81/99 (82%) were concordant for CTC-ETI category between baseline and M1: 1 moved from CTC-ETI “low” to “intermediate”; 6 moved from “intermediate” to “low” (*N*=3) or

to “high” (*N*=3); 11 moved from “high” to “low” (*N*=7) or to “intermediate” (*N*=4) (**Supplementary Table 4**). In addition, 77/94 (82%), 66/85 (78%) were concordant for CTC-ETI category between baseline and M2 and M3, respectively.

**Changes in CTC enumeration at subsequent time points from baseline**

Among the 95 patients with M1 sample, only one patient with high CTC levels at M1 were increased from low CTC levels at baseline. Twenty-one patients’ CTC levels remained high, 62 remained low, and 11 decreased from high to low. The median PFS from time of sample collection among patients with high, low, and decreased CTC was 1.9, 5.0, and 7.6 months, respectively (**Supplementary Figure 1a**). The results were similar for the 91 patients with CTC levels determined at M2 (median PFS of 1.2, 5.0, 6.7 months from the sample collection) and for the 83 patients with CTC levels determined at M3 (0.3, 5.8, and 5.7 months from sample collection) (**Supplementary Figures 1b, c**).

**Retraining CTC-ETI**

The distribution of PFS was estimated according to the pre-defined categorization of percentage ER (**Supplementary Figure 3)** in a subset of 40 patients with ≥5 CTC in aliquot of WB in which CTC-ER was assessed at baseline, with estimated median PFS of 3.7, 2.8, and 3.4 months for the 0%, 1-10%, and >10% ER categories respectively, consistent with other results.

In order to evaluate model performance, the ROC curves and AUCs for both models were compared to the ROC curve and AUC based on the average CTC count of all 32 patients (**Supplementary Figure 2a**). The ROC curve with the highest AUC (0.8593) was average CTC count, followed by the AUC (0.8009) for the random forest models and the AUC (0.7749) for the logistic models. When examining variable importance of the 500 trees, average CTC count was

the most important variable in 444 (89%) of them. Ki67 was the most important variable in 50 (10%), BCL2 in 3 (0.6%), HER2 in 3 (0.6%), and ER in 0 (0%).

With an interest of determining which of the biomarkers had any prognostic value outside of average CTC, we reran the random forest and logistic models without average CTC to see how the biomarkers would perform in its absence. The ROC curve with the highest AUC (0.8593) was average CTC (as noted above), followed by the AUC (0.7316) for logistic models and the AUC (0.6883) for the random forests (**Supplementary Figure 2b**). Both the logistic models and random forests without average CTC had lower AUCs than average CTC alone. Ki67 was the most important variable in 318 (64%) of trees, followed by ER in 128 (26%), BCL2 in 31 (6%) and HER2 in 23 (5%). Ki67 being the most important biomarker in a majority of trees was consistent with the modeling that included average CTC. ER being the most important biomarker in 26% of trees was unexpected, given that it was never the most important variable in the presence of average CTC.

Further exploration of the relationship of Ki67 and ER with rapid progression used univariate logistic regression models including all 32 patients deemed eligible for this analysis. The parameter estimates were generally consistent with the CTC-ETI assumptions, suggesting odds of rapid progression increased as percentage of Ki67 increased and as percentage of ER decreased. The model for percentage Ki67 produced a parameter estimate of 0.11 (95% CI: 0.02 to 0.20) and an odds ratio of 1.1 (95% CI: 1.0 to 1.2). The model for percentage ER yielded a parameter estimate of -0.02 (95% CI: -0.06 to +0.02) and odds ratio of 0.98 (95% CI: 0.95 to 1.02).

This exploratory analysis confirmed the limited ability of CTC phenotypes, measured as percentage of CTC having marker positivity, to add to CTC enumeration, and an inability to

refine the CTC-ETI in this trial cohort. These analyses were limited by a very small sample size because of the small number of patients having ≥5 CTC/7.5 ml WB at baseline. An analysis with a larger sample size of patients with elevated CTC may yield more definitive results.

**REFERENCES**

1 Paoletti, C. *et al.* Development of circulating tumor cell-endocrine therapy index in patients with hormone receptor-positive breast cancer. *Clin Cancer Res* **21**, 2487-2498, doi:10.1158/1078-0432.CCR-14-1913 (2015).

## SUPPLEMENTARY TABLES (Table 1 modified from previously reported reference[1])

**Supplementary Table 1.**

**a.** CTC-Enumeration Points based on CTC Enumeration

| | Low (Favorable) | Intermediate | | High (Unfavorable) |
|---|---|---|---|---|
| **Number of CTC/7.5 ml WB (mean of 4 aliquots)** | <5 | 5-10 | 11-100 | > 100 |
| **CTC-Enumeration Points** | 0 | 1 | 3 | 4 |

**b.** CTC-Bio-Points[a]

| | Presumed Prediction of Response to ET | | | | | |
|---|---|---|---|---|---|---|
| | **Favorable** | | **Intermediate** | | **Unfavorable** | |
| | *% CTC positive* | *Assigned Bio-Points* | *% CTC positive* | *Assigned Bio-Points* | *% CTC positive* | *Assigned Bio-Points* |
| ***ER*** | >10% | 0 | 1-10%[b] | 2 | 0%[b] | 6[b] |
| ***BCL2*** | >10% | 0 | 1-10% | 1 | 0% | 2 |
| ***HER2*** | 0% | 0 | 1-10% | 1 | >10% | 2 |
| ***Ki67*** | 0% | 0 | 1-10% | 1 | >10% | 2 |

[a]Only aliquots with ≥5 CTC/7.5 ml WB evaluated

[b]Low or negative ER expression is weighed more than BCL2, HER2, and Ki67 due to its fundamental role in endocrine responsiveness.

**c.** Modified Calculation of CTC-ETI Scores[a]

| Average CTC Counts | CTC-Enumeration Points | Marker | CTC-Bio-Points | | | Potential CTC-ETI Score (sum of CTC Assigned and Bio-Points) |
|---|---|---|---|---|---|---|
| | | | *Low* | *Intermediate* | *High* | |
| ***CTC <5*** | 0 | N/A | 0 | 0 | 0 | 0 |
| ***CTC 5-10*** | 1 | ER | 0 | 2 | 6 | 1-13 |
| | | BCL2 | 0 | 1 | 2 | |
| | | HER2 | 0 | 1 | 2 | |
| | | Ki67 | 0 | 1 | 2 | |
| ***CTC 11-100*** | 3 | ER | 0 | 2 | 6 | 3-15 |
| | | BCL2 | 0 | 1 | 2 | |
| | | HER2 | 0 | 1 | 2 | |
| | | Ki67 | 0 | 1 | 2 | |
| ***CTC >100*** | 4 | ER | 0 | 2 | 6 | 4-16 |
| | | BCL2 | 0 | 1 | 2 | |
| | | HER2 | 0 | 1 | 2 | |
| | | Ki67 | 0 | 1 | 2 | |

[a]In the original CTC-ETI algorithm, there were only 3 CTC-enumeration point categories. The modified CTC-ETI algorithm separated the previously highest CTC-enumeration point category (>10 CTC) into 10-100 and >100 CTC categories, which now account for 3 and 4 CTC-enumeration points respectively, increasing the maximum CTC-ETI score from 14 to 16.

**d.** CTC-ETI Categories

| **Category** | **CTC-ETI** | **Presumed Outcome for Patient with ER Positive MBC starting new ET** | **Presumed Clinical Action** |
|---|---|---|---|
| ***Low*** | 0-3 | Favorable; respond to ET and/or indolent disease; long time to progression | Treat with ET |
| ***Intermediate*** | 4-6 | Probably respond or moderately indolent disease; modest time to progression | Treat with ET |
| ***High*** | 7-16 | Poor; Resistant to ET, rapid progression | Treat as ER negative with chemotherapy |

**Abbreviations:** CTC= circulating tumor cells; ER= estrogen receptor; ET= endocrine therapy; MBC= metastatic breast cancer.

**Supplementary Table 2. Concordance of two CTC-ETI readings as classified by UM laboratory original read and reread**

**a.** Comparison of CTC-ETI category

| Original (UM) CTC-ETI Category | UM Reread CTC-ETI Category | | | | Total |
|---|---|---|---|---|---|
| | High (7 - 16) | Intermediate (4 - 6) | Low (0 - 3) | Unable to be determined | |
| | *N* | *N* | *N* | *N* | *N* |
| High (7 - 16) | 7 | - | - | - | 7 |
| Intermediate (4 - 6) | - | 2 | - | - | 2 |
| Low (0 - 3) | - | - | 1 | - | 1 |
| Unable to be determined | - | - | - | 2 | 2 |
| Total | 7 | 2 | 1 | 2 | 12 |

**b.** Comparison of CTC-ETI score

| Original (UM) CTC-ETI Score | UM Reread CTC-ETI Score | | | | | | | | Total |
|---|---|---|---|---|---|---|---|---|---|
| | . | 3 | 4 | 5 | 7 | 8 | 9 | 13 | |
| | *N* | *N* | *N* | *N* | *N* | *N* | *N* | *N* | *N* |
| . | 2 | - | - | - | - | - | - | - | 2 |
| 3 | - | 1 | - | - | - | - | - | - | 1 |
| 4 | - | - | 1 | - | - | - | - | - | 1 |
| 5 | - | - | - | 1 | - | - | - | - | 1 |
| 7 | - | - | - | - | 1 | - | - | - | 1 |
| 8 | - | - | - | - | - | 1 | 1 | - | 2 |
| 9 | - | - | - | - | 1 | - | 1 | - | 2 |
| 11 | - | - | - | - | - | - | - | 1 | 1 |
| 13 | - | - | - | - | - | - | - | 1 | 1 |
| Total | 2 | 1 | 1 | 1 | 2 | 1 | 2 | 2 | 12 |

**Supplementary Table 3. Concordance of two CTC-ETI category readings between original laboratory (Mayo) and UM laboratory reread**

**a**. Comparison of CTC-ETI category

| Original (Mayo) CTC-ETI Category | UM Reread CTC-ETI Category | | | | Total |
|---|---|---|---|---|---|
| | High (7 - 16) | Intermediate (4 - 6) | Low (0 - 3) | Unable to be determined | |
| | *N* | *N* | *N* | *N* | *N* |
| **High (7 - 16)** | 16 | 4 | - | - | 20 |
| **Intermediate (4 - 6)** | - | 1 | - | - | 1 |
| **Low (0 - 3)** | - | - | 72 | 4 | 76 |
| **Unable to be determined** | 1 | - | 1 | 4 | 6 |
| **Total** | 17 | 5 | 73 | 8 | 103 |

**b**. Comparison of CTC-ETI score

| Original (Mayo) CTC-ETI Score | UM Reread CTC-ETI Score | | | | | | | | | | Total |
|---|---|---|---|---|---|---|---|---|---|---|---|
| | . | 0 | 5 | 6 | 7 | 8 | 9 | 10 | 12 | 13 | 15 | |
| | *N* | *N* | *N* | *N* | *N* | *N* | *N* | *N* | *N* | *N* | *N* | *N* |
| . | 2 | 16 | - | - | - | - | 1 | - | - | - | - | 19 |
| 0 | 2 | 59 | - | - | - | - | - | - | - | - | - | 61 |
| 1 | 1 | - | - | - | - | - | - | - | - | - | - | 1 |
| 3 | 1 | - | - | - | - | - | - | - | - | - | - | 1 |
| 4 | - | - | 1 | - | - | - | - | - | - | - | - | 1 |
| 7 | - | - | - | - | 1 | 1 | - | - | - | - | - | 2 |
| 8 | - | - | 1 | - | 2 | - | - | - | - | - | - | 3 |
| 9 | - | - | - | 1 | - | 1 | - | 1 | - | - | - | 3 |
| 10 | - | - | - | 1 | 1 | - | - | 1 | - | - | - | 3 |
| 11 | - | - | - | - | - | - | - | - | - | 1 | - | 1 |
| 12 | - | - | - | 1 | - | - | - | - | 1 | - | - | 2 |
| 13 | - | - | - | - | - | - | 1 | - | 2 | 1 | - | 4 |
| 14 | - | - | - | - | - | - | - | - | - | - | 1 | 1 |
| 15 | - | - | - | - | - | - | - | - | - | - | 1 | 1 |
| Total | 6 | 75 | 2 | 3 | 4 | 2 | 2 | 2 | 3 | 2 | 2 | 103 |

**Supplementary Table 4. Concordance of two CTC-ETI category readings between baseline and serial samples, according to time point**

| | Time point CTC-ETI | | | | | | | | | | | | | |
|---|---|---|---|---|---|---|---|---|---|---|---|---|---|---|
| | M1 | | | | M2 | | | | | M3 | | | | |
| | 1:Low (0-3) | 2:Intm (4-6) | 3:High (7-16) | Total | . | 1:Low (0-3) | 2:Intm (4-6) | 3:High (7-16) | Total | . | 1:Low (0-3) | 2:Intm (4-6) | 3:High (7-16) | Total |
| | *N* | *N* | *N* | *N* | *N* | *N* | *N* | *N* | *N* | *N* | *N* | *N* | *N* | *N* |
| **Baseline CTC-ETI** | | | | | | | | | | | | | | |
| 1:Low (0-3) | 65 | 1 | - | 66 | 1 | 62 | - | 2 | 65 | 1 | 58 | 1 | 3 | 63 |
| 2:Intm (4-6) | 3 | 1 | 3 | 7 | - | 4 | - | 1 | 5 | - | 3 | - | 2 | 5 |
| 3:High (7-16) | 7 | 4 | 15 | 26 | - | 8 | 1 | 15 | 24 | - | 9 | - | 8 | 17 |
| Total | 75 | 6 | 18 | 99 | 1 | 74 | 1 | 18 | 94 | 1 | 70 | 1 | 13 | 85 |

**a**

| M1 CTC status | Patients | Events | Median PFS from landmark |
|---|---|---|---|
| Decrease | 11 (12%) | 9 | 7.6 |
| Increase | 1 (1%) | 1 | -- |
| High | 21 (22%) | 18 | 1.9 |
| Low | 62 (65%) | 43 | 5.0 |

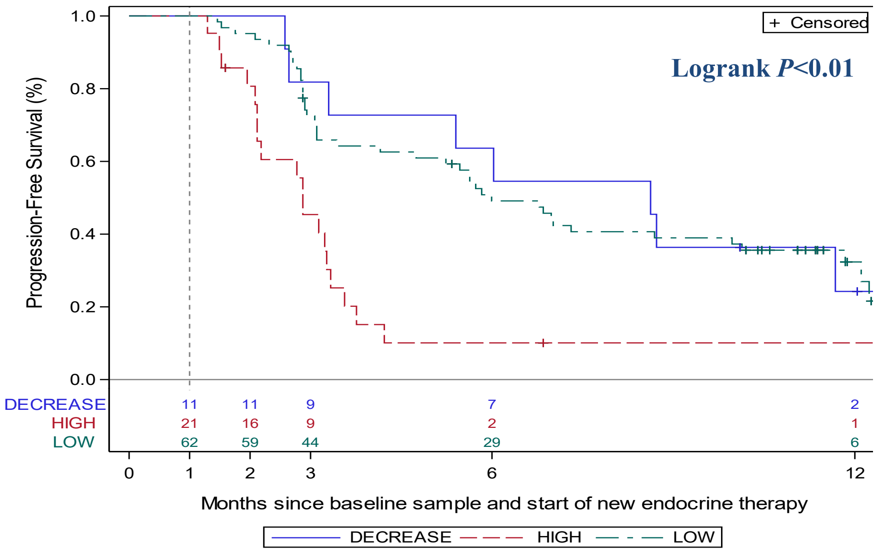


**b**

| M2 CTC status | Patients | Events | Median PFS from landmark |
|---|---|---|---|
| Decrease | 14 (15%) | 10 | 6.7 |
| Increase | 3 (3%) | 3 | 0.9 |
| High | 17 (19%) | 15 | 1.2 |
| Low | 57 (63%) | 38 | 5.0 |

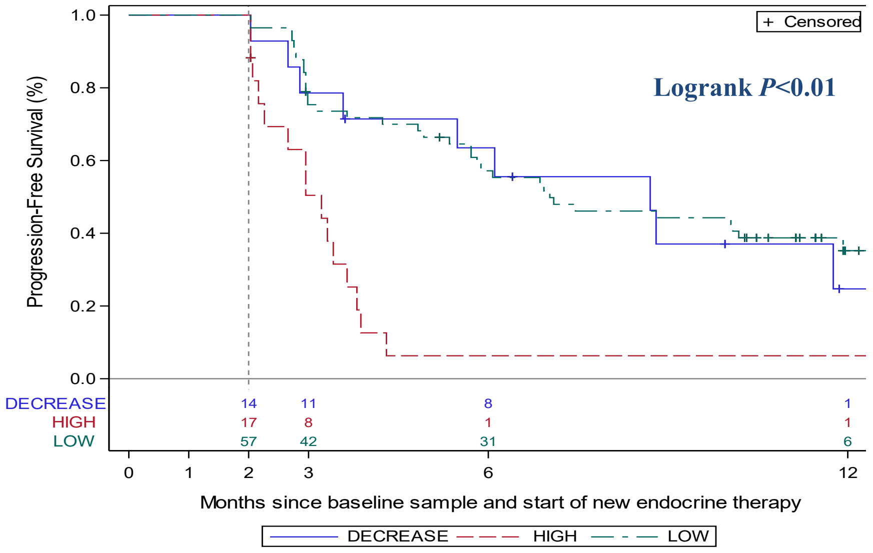


**Supplementary Figure 1.** Progression-free survival (PFS) according to changes in circulating tumor cell (CTC) enumeration change at subsequent blood draw time points from baseline.

(Blue line, decrease [≥5 CTC→<5 CTC/7.5 ml whole blood]; Red line, high [≥5 CTC→≥5 CTC/7.5 ml whole blood]; Green line, low [<5 CTC→<5 CTC/7.5 ml whole blood])

**a.** Baseline to Month 1 (M1):

*N*=99 patients with M1 sample. Only 95 patients were analyzed in landmark analysis of PFS (*N*=4 PFS censored on D1, no re-imaging)

**b.** Baseline to Month 2 (M2):

*N*=94 patients with M2 sample. Only 91 patients were analyzed in a landmark analysis of PFS (*N*=2 PFS censored at D1, no re-imaging; *N*=1 no CTC count)

a-c: vertical dashed line indicates sample time point landmark; median PFS is calculated from this landmark.

**c**

| M3 CTC status | Patients | Events | Median PFS from landmark |
|---|---|---|---|
| Decrease | 14 (17%) | 10 | 5.7 |
| Increase | 5 (6%) | 5 | 0.0 |
| High | 10 (12%) | 9 | 0.3 |
| Low | 54 (65%) | 35 | 5.8 |

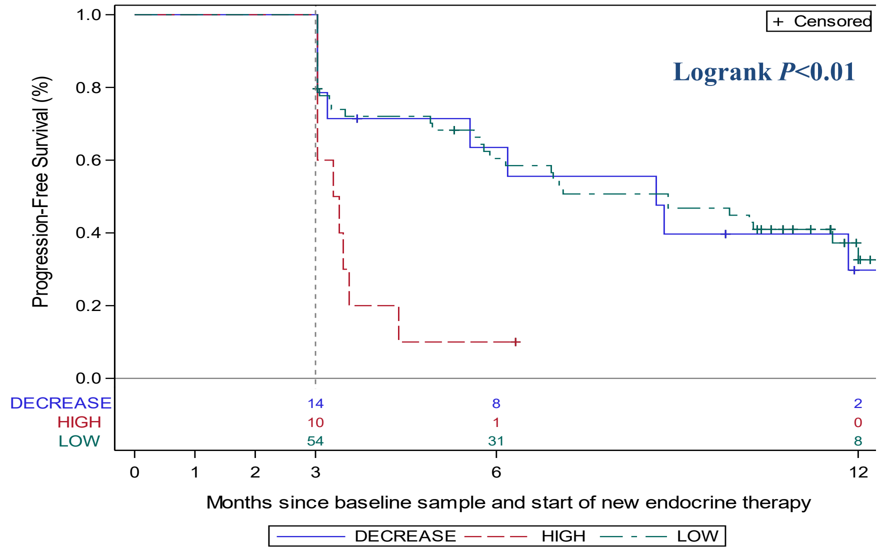


**Supplementary Figure 1.** Progression-free survival (PFS) according to changes in circulating tumor cell (CTC) enumeration change at subsequent blood draw time points from baseline.

(Blue line, decrease [≥5 CTC→<5 CTC/7.5 ml whole blood]; Red line, high [≥5 CTC→≥5 CTC/7.5 ml whole blood]; Green line, low [<5 CTC→<5 CTC/7.5 ml whole blood])

**c.** Baseline to Month 3 (M3):
*N*=85 patients with a M3 sample. Only 83 patients were analyzed in a landmark analysis of PFS (N=1 PFS censored on D1, no re-imaging; N=1 no CTC count)

a-c: vertical dashed line indicates sample time point landmark; median PFS is calculated from this landmark.

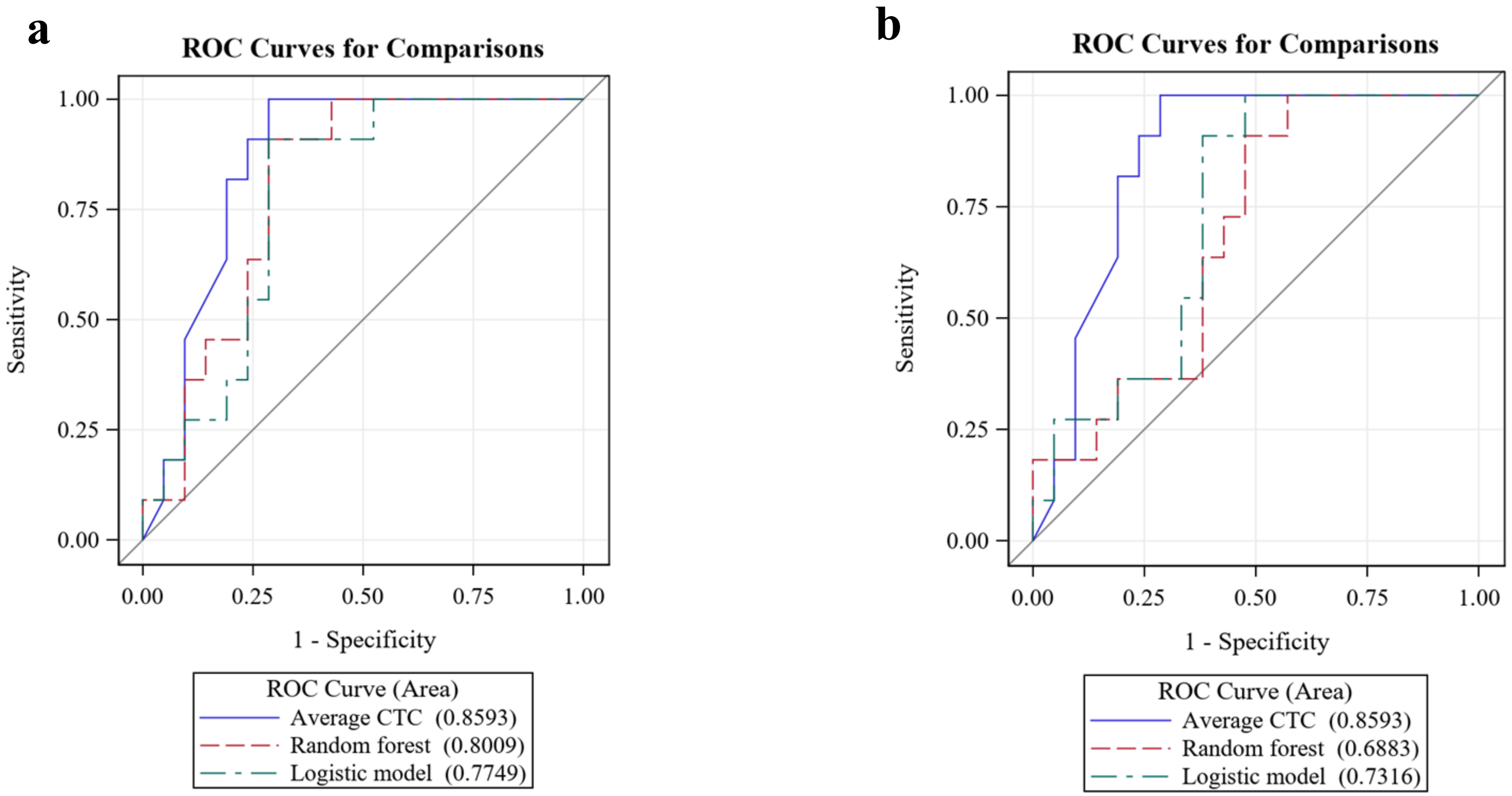


**Supplementary Figure 2**. Receiver Operating Characteristic (ROC) curves for random forest models, logistic regression models, and average CTC.

**a.** The random forest and logistic regression models included average CTC and biomarker percentages.

**b.** The random forest and logistic regression models included biomarker percentages.

| BL CTC-ER status | Patients | Events | Median PFS |
|---|---|---|---|
| 0% CTC-ER | 14 (35%) | 10 | 3.7 |
| 1-10% CTC-ER | 11 (27.5%) | 10 | 2.8 |
| >10% CTC-ER | 15 (37.5%) | 10 | 3.4 |

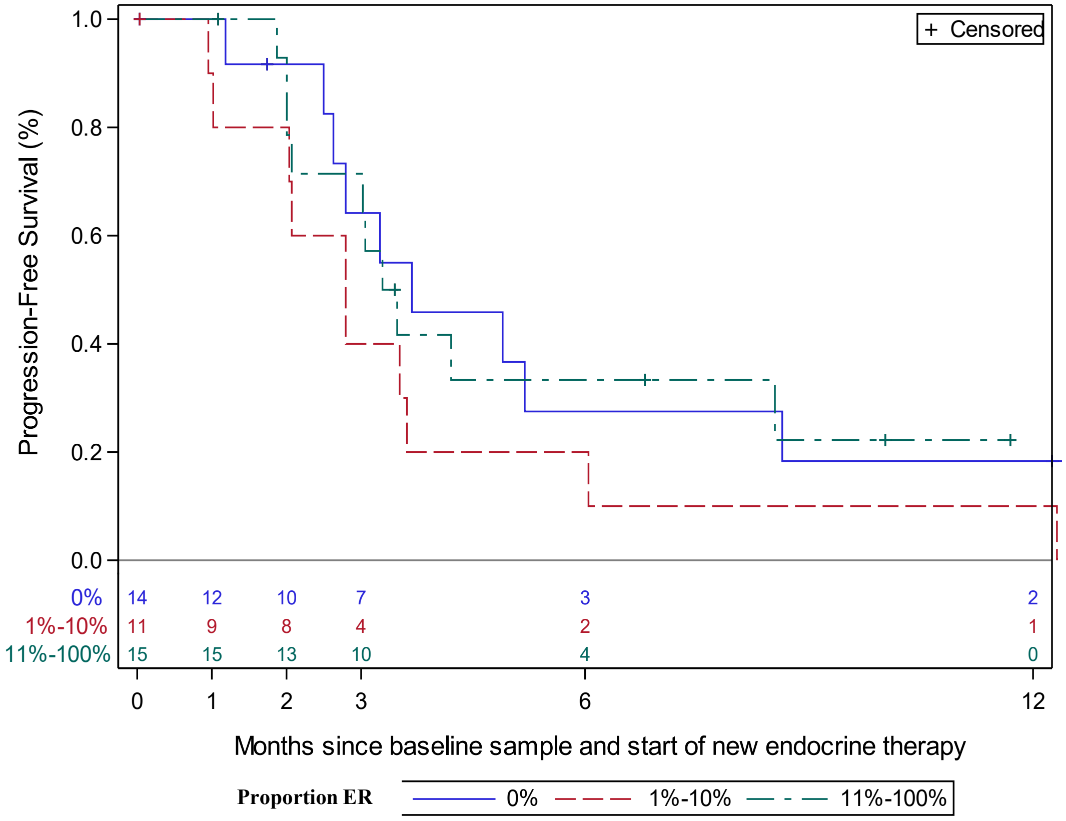


**Supplementary Figure 3.** Progression-free survival (PFS) according to proportion of CTC-ER staining in subset of 40 patients with ≥5 CTC (0%, 1-10%, and >10%) in aliquot of WB in which CTC-ER was assessed at baseline (BL)

(Blue line, 0% CTC-ER; Red line, 1-10% CTC-ER; Green line, >10% ER).

Eligibility:
ER positive, HER2 negative, progressive MBC starting new Endocrine Therapy

| | BL[a] | M1[b] | M2[b] | M3[b] | M6[b] | M9[b] | M12/EOS[b,c] |
|---|---|---|---|---|---|---|---|
| Medical Record Review/History | X | | | | | | |
| Physician (or Designee): Assessment of Clinical Status | X | X | X | X | X | X | X |
| Blood draw for CTC-ETI | X | X | X | X | | | X |
| Imaging Assessments: CT or MRI scan (minimum: chest & abdomen) plus Bone or PET scan OR PET-CT | X | | | X | ← Physician Discretion | → | X |
| Optional: Provision of Archival Tissue Specimens (blocks or slides) | X | | | | | | |

[a]Baseline Assessments: Must be done within 30 days prior to initiation of new endocrine therapy.
[b]Follow-up Assessments: Must be performed +/- 14 days of designated time point. Frequency of clinical follow-up is every 3 months. Imaging between 2-12 months is at the discretion of the managing physician, however, all target and/or non-target lesions selected at baseline must be assessed at 3 months and at progression/EOS/12 months with the same imaging modality used at baseline (RECIST v1.1 guidelines).
[c]M12/EOS Assessments: Must be done at the time the patient is taken off the study (i.e. at disease progression, discontinuation of therapy or 12 months after the initiation of therapy without disease progression, whichever occurs first). All target and/or non-target lesions selected at baseline must be assessed with the same imaging modality used at baseline (RECIST v1.1 guidelines).

**Supplementary Figure 4**. Study schema. Abbreviations: BL= baseline; M1= month 1; M2= month 2, M3= month 3, M6= month 6, M9= month 9, M12= month 12, EOS= end of study.

Janssen Diagnostics, LLC
700 Route 202 South
Raritan, NJ 08869

# CLINICAL STUDY PROTOCOL

| | |
|---|---|
| **PROTOCOL NUMBER:** | COMETI-P2-2012.0 |
| **PROTOCOL TITLE:** | COMETI Phase 2: Characterization of Circulating Tumor Cells from Subjects with Metastatic Breast Cancer Using the CTC-Endocrine Therapy Index |
| **IDE NUMBER:** | NA; Non-significant risk study |
| **ORIGINAL PROTOCOL DATE:** | September 21, 2012 |
| **AMENDMENT 1 DATE:** | January 14, 2013 |
| **AMENDMENT 2 DATE:** | April 3, 2013 |
| **AMENDMENT 3 DATE:** | December 22, 2014 |
| **SPONSOR:** | Janssen Diagnostics, LLC*<br>700 Route 202 South<br>Raritan, NJ 08869 |



| | |
|---|---|
| **PRINCIPAL INVESTIGATOR:** | Daniel F. Hayes, M.D.<br>Phone: 734-615-6725<br>Fax: 734-647-9271<br>E-Mail: hayesdf@med.umich.edu |
| **CO-PRINCIPAL INVESTIGATOR:** | Costanza Paoletti, M.D.<br>Phone: 734-647-7250<br>Fax: 734-647-9480 or 734-647-9271<br>E-mail: pcostanz@med.umich.edu |
| **STATISTICIAN:** | Meredith M. Regan, ScD<br>Phone: 617-632-2471<br>Fax: 617-632-2444<br>E-mail: mregan@jimmy.harvard.edu |
| **STUDY MANAGER:** | Robert McCormack, Ph.D.<br>Office: 908-927-4824<br>Mobile: 908-625-2588<br>E-Mail: Rmccorma@its.jnj.com |



**Compliance:** This study will be conducted in compliance with this protocol, Good Clinical Practice (GCP), and applicable regulatory requirements.

*Janssen Research & Development is a global organization that operates through different legal entities in various countries. Therefore, the legal entity acting as the sponsor for Janssen Research & Development studies may vary, such as, but not limited to Janssen Diagnostics, LLC; Janssen Biotech, Inc.; Janssen Products, LP; Janssen Biologics, BV; Janssen-Cilag International, NV; Janssen, Inc; or Janssen Research & Development, LLC. The term "sponsor" is used throughout the protocol to represent these various legal entities; the sponsor is identified on the Contact Information page that accompanies the protocol.

**Confidentiality Statement**

Janssen Diagnostics, LLC
700 Route 202 South
Raritan, NJ 08869

The information in this document contains trade secrets and commercial information that are privileged or confidential and may not be disclosed unless such disclosure is required by applicable law or regulations. In any event, persons to whom the information is disclosed must be informed that the information is *privileged* or *confidential* and may not be further disclosed by them. These restrictions on disclosure will apply equally to *all* future information supplied to you that is indicated as *privileged* or *confidential*.

# CLINICAL STUDY PROTOCOL APPROVAL PAGE

| | |
|---|---|
| **PROTOCOL NUMBER:** | COMETI-P2-2012.0 |
| **PROTOCOL TITLE:** | COMETI Phase 2: Characterization of Circulating Tumor Cells from Subjects with Metastatic Breast Cancer Using the CTC-Endocrine Therapy Index |
| **IDE NUMBER:** | NA; Non-significant risk study |
| **ORIGINAL PROTOCOL DATE:** | September 21, 2012 |
| **AMENDMENT 1 DATE:** | January 14, 2013 |
| **AMENDMENT 2 DATE:** | April 3, 2013 |
| **AMENDMENT 3 DATE:** | December 22, 2014 |

**APPROVED BY:**

| | | |
|---|---|---|
| *Nicholas Dracopoli, Ph.D.* | *Head – Oncology Biomarkers* | *Date* |
| *Robert McCormack, Ph.D.* | *Sr. Director – Oncology Biomarkers* | *Date* |
| *Scott Adams* | *Assoc Dir, Clinical Research* | *Date* |

# TABLE OF CONTENTS

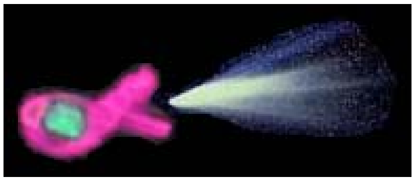

**1.0 STUDY SUMMARY ......................................................................... 6**
- **1.1 STUDY OBJECTIVES** ........................................................................ 6
- **1.2 STUDY DESIGN** .............................................................................. 7
- **1.3 TEST ARTICLE** ............................................................................... 7
- **1.4 STUDY SITES** ................................................................................. 8
- **1.5 STUDY POPULATION** ......................................................................... 8
- **1.6 SAMPLE SIZE** ................................................................................. 8
- **1.7 METHODS** ...................................................................................... 8

**2.0 ABBREVIATIONS AND DEFINITIONS .................................................. 11**

**3.0 INTRODUCTION AND STUDY RATIONALE ........................................ 12**

**4.0 STUDY OBJECTIVES AND DURATION ................................................ 15**
- **4.1 STUDY OBJECTIVES** ........................................................................ 15
- **4.2 STUDY DURATION** ........................................................................... 16

**5.0 DEVICE DESCRIPTION AND STUDY SUPPLIES ................................ 16**
- **5.1 ASSAY/DEVICE DESCRIPTIONS** ........................................................ 16
  - 5.1.1 CXC Assay ..................................................................................... 17
  - 5.1.2 CXC Assay Controls ......................................................................... 17
  - 5.1.3 Marker Controls .............................................................................. 18
  - 5.1.4 BioMarQ Software ............................................................................ 18
- **5.2 CALCULATION OF CTC-ETI** .............................................................. 19
- **5.3 STUDY SUPPLIES, STORAGE, USE AND ACCOUNTABILITY** ................... 21
  - 5.3.1 Clinical Sites ................................................................................... 21
  - 5.3.2 Study Laboratory Sites ..................................................................... 21

**6.0 SUBJECT SELECTION ...................................................................... 22**
- **6.1 INCLUSION CRITERIA** ...................................................................... 22
- **6.2 EXCLUSION CRITERIA** ..................................................................... 23

**7.0 STUDY LABORATORY TRAINING AND PROFICIENCY TESTING 24**

**8.0 STUDY METHODS & PROCEDURES ................................................. 24**
- **8.1 INVESTIGATIONAL SITES** ................................................................. 24
- **8.2 STUDY SUBJECT SELECTION AND REGISTRATION** ............................. 25
- **8.3 BLINDING & USE OF CTC** ................................................................. 25

8.3.1 Blinding of CTC-ETI assay operators ........ 25
8.3.2 Blinding of clinicians ........ 26
8.3.3 Use of CTC as standard of care ........ 26
**8.4 STUDY SCHEMA** ........ 26
**8.5 ALLOWABLE TREATMENT(S)** ........ 27
**8.6 IMAGING** ........ 27
**8.7 BLOOD SAMPLE COLLECTION & HANDLING PROCEDURES** ........ 28
**8.8 UNANTICIPATED ADVERSE DEVICE EFFECTS** ........ 28
**8.9 BLOOD SAMPLE PROCESSING PROCEDURES** ........ 29
8.9.1 Pooling ........ 29
8.9.2 CTC/Marker Enumeration ........ 29
**8.10 TISSUE SAMPLE COLLECTION & HANDLING PROCEDURES** ........ 30
**8.11 TISSUE SAMPLE PROCESSING PROCEDURES** ........ 31

**9.0 IMAGING MEASUREMENT AND INTERPRETATION CRITERIA .32**
**9.1 MEASURABILITY OF LESIONS** ........ 32
**9.2 SELECTION OF TARGET AND NON-TARGET LESIONS** ........ 33
**9.3 DETERMINATION OF OBJECTIVE TUMOR RESPONSE** ........ 33
**9.4 DEFINITION OF RAPID DISEASE PROGRESSION** ........ 36
**9.5 DEFINITION OF PROGRESSION FREE SURVIVAL (PFS)** ........ 36

**10.0 SUBJECT COMPLETION/WITHDRAWAL ........36**
**10.1 COMPLETION** ........ 36
**10.2 DISCONTINUATION OF TREATMENT** ........ 36
**10.3 WITHDRAWAL FROM THE STUDY** ........ 37

**11.0 DATA COLLECTION AND HANDLING........38**
**11.1 CASE REPORT FORMS** ........ 38
**11.2 CASE REPORT FORM COMPLETION** ........ 39
**11.3 DATA QUALITY ASSURANCE / QUALITY CONTROL** ........ 40
**11.4 PRIVACY OF PERSONAL DATA** ........ 40

**12.0 STATISTICAL CONSIDERATIONS ........41**
**12.1 SAMPLE SIZE** ........ 41
**12.2 1ST PRIMARY OBJECTIVE** ........ 41
**12.3 2ND PRIMARY OBJECTIVE** ........ 42
**12.4 3RD PRIMARY OBJECTIVE** ........ 44
**12.5 SECONDARY OBJECTIVES** ........ 44
**12.6 DEFINITIONS OF EVALUABLE** ........ 45
**12.7 ADDITIONAL ANALYSES** ........ 45
**12.8 GUIDELINES FOR FAILURE/FUTILITY AND MONITORING** ........ 45

**13.0 REGULATORY/ETHICAL REQUIREMENTS ........47**
**13.1 INVESTIGATOR RESPONSIBILITIES:** ........ 47
**13.2 INDEPENDENT ETHICS COMMITTEE OR INSTITUTIONAL REVIEW BOARD** ........ 47
**13.3 INFORMED CONSENT** ........ 49

Janssen Diagnostics, LLC
700 Route 202 South
Raritan, NJ 08869

13.4 LONG-TERM STORAGE OF SAMPLES FOR FUTURE RESEARCH ........ 50

**14.0 ADMINISTRATIVE REQUIREMENTS ........ 50**

14.1 MODIFICATION OF THE PROTOCOL (AMENDMENTS) ........ 50
14.2 REQUIRED PRE-STUDY DOCUMENTATION ........ 51
14.3 SUBJECT IDENTIFICATION, ENROLLMENT, AND SCREENING LOGS ........ 51
14.4 SOURCE DOCUMENTATION ........ 52
14.5 RECORD RETENTION ........ 52
14.6 MONITORING ........ 53

**15.0 STUDY COMPLETION AND TERMINATION ........ 53**

15.1 STUDY COMPLETION ........ 53
15.2 STUDY TERMINATION ........ 53

**16.0 INVESTIGATOR STATEMENT AND AGREEMENT ........ 55**

**17.0 REFERENCES ........ 56**

## APPENDICES

**Appendix A – Example Informed Consent Form……..58**
**Appendix B – Example Study Logs & CRFs……. 78**
**Appendix C – Blood Sample Shipping & Handling Instructions……...102**
**Appendix D – Tissue Sample Shipping & Handling Instructions……...105**
**Appendix E – Determination of CTC-Assigned Points……106**

**CONTACT INFORMATION SHEET……...112**

Janssen Diagnostics, LLC
700 Route 202 South
Raritan, NJ 08869

# 1.0 STUDY SUMMARY

Metastatic breast cancer (MBC) patients with estrogen receptor (ER) negative breast cancer have almost no chance of benefit from anti-estrogen (or "endocrine") therapies, and are therefore treated with more toxic, but more likely beneficial, chemotherapy. Patients with ER positive MBC initiating first line endocrine therapy (ET) have only a 30-50% chance of receiving clinical benefit. For the other 50-70%, ET is ineffective and these patients should probably be treated with chemotherapy, as is done for ER negative patients. Unfortunately, there is no easy way to identify which ER positive patients will most likely respond to therapy and those that will not.

Utilizing CELLSEARCH® technology, we have demonstrated our ability to both enumerate and reliably and reproducibly characterize circulating tumor cells (CTC) for tumor markers that predict for endocrine sensitivity (ER and Bcl-2) and resistance (HER2 and Ki67). We have generated an algorithm for a CTC-Endocrine Therapy Index (CTC-ETI) that can be calculated for each subject using the CTC enumeration and marker results. The primary goal of this study is to determine a CTC-ETI in ER positive, HER2 negative MBC patients before the initiation of a new ET that can identify those subjects that will progress rapidly.

## 1.1 STUDY OBJECTIVES

The primary objectives of this study are to:

- Demonstrate that the CTC-ETI can be accurately determined at initiation of a new ET (baseline) in subjects with ER positive, HER2 negative MBC in multiple centers across North America (analytical validity);
- Determine if the current CTC-ETI algorithm is associated with rapid progression, defined as progression within three (3) months of starting a new ET in subjects with ER positive, HER2 negative MBC (clinical validity);
- Refine the current CTC-ETI algorithm, if necessary, so that it accurately predicts rapid progression in subjects with ER positive, HER2 negative MBC starting a new ET.

The secondary objectives of this study are to:

- Determine if a CTC-ETI can be accurately determined at serial time points during treatment in subjects with ER positive, HER2 negative MBC after starting a new ET in multiple centers across North America.
- Determine if the current/refined CTC-ETI algorithm is associated with progression free survival (PFS) in subjects with ER positive, HER2 negative MBC after starting a new ET who have been followed for up to 12 months.
- Determine changes in CTC-ETI during ET and see if these changes are associated with outcomes (i.e. rapid progression and PFS).
- Correlate the status of the biomarkers on baseline CTC with the status of the same biomarkers in primary and/or metastatic tissue collected from the subjects.

PRODUCTS ARE FOR RESEARCH USE ONLY AND THEIR PERFORMANCE CHARACTERISTICS ON CLINICAL SAMPLES HAVE NOT BEEN DETERMINED

Janssen Diagnostics, LLC
700 Route 202 South
Raritan, NJ 08869

- Store CTC positive cartridges for future molecular analyses.
- Extract and store cell-free DNA for future analyses.

### 1.2 STUDY DESIGN

This will be a multi-center, prospective study. All subjects will have blood drawn for CTC-ETI determination at baseline (within 30 days prior to the initiation of ET). The CTC-ETI data will not be reported back to the physicians and it will not be used for patient/disease management. The only exception to this is that physicians will be notified by the Sponsor when a subject has an unsuccessful baseline CTC-ETI calculation, as these subjects will be removed from further participation in the study. Subjects with a successful baseline CTC-ETI calculation will have subsequent serial blood draws performed 1, 2, 3 and up 12 months after the initiation of ET, or at the time of disease progression or discontinuation of treatment, whichever occurs first. Subjects with unsuccessful baseline CTC-ETI calculations will be excluded from further blood draws and will not be followed for progression. For each subject with a successful baseline CTC-ETI calculation, we will also attempt to obtain archived tumor specimens, if available, from the primary and metastatic cancers.

At pre-specified subject accrual points, we will assess the CTC-ETI testing for analytical validity and the study design for futility. Upon completion of the enrollment (if the trial has not been stopped for futility reasons) and after a minimum of 3 months of follow-up for all subjects evaluable for clinical validity, the CTC-ETI algorithm will be assessed and, if necessary, refined to identify subjects who will experience rapid progression with high specificity (low false-positive fraction). Rapid progression will be defined as disease progression according to RECIST v1.1 criteria or death due to MBC within 3 months of starting a new ET. PFS will be measured as the time from the date of starting ET until the date of first documentation of progressive disease according to RECIST v1.1 criteria or death due to any cause. In the absence of these events, PFS will be censored at the date of the last objective disease assessment (up to a maximum of 12 months after the initiation of ET). The refined CTC-ETI algorithm will be validated in a future study designed for submission to the FDA to establish its positive predictive value.

### 1.3 TEST ARTICLE

**All assays performed on the blood collected specifically for the purposes of this study are investigational products and their performance characteristics on clinical samples have not been determined.** The Research Use Only (RUO) CELLSEARCH® CXC Kit will be used for the isolation and staining of CTC. The following tumor profiling antibodies, conjugated to phycoerythrin (PE), will be used to characterize the isolated CTC: ER (monoclonal antibody ER-119.3), Bcl-2 (monoclonal antibody Bcl-2 (100), BD Pharmingen), CELLSEARCH® Tumor Phenotyping Reagent HER2/neu Kit for HER2 (monoclonal antibody HER 81), and Ki67 (monoclonal antibody B56, BD Pharmingen).

Janssen Diagnostics, LLC
700 Route 202 South
Raritan, NJ 08869

### 1.4 Study Sites

It is anticipated that we will have approximately 25 clinical sites located within the United States and Canada enrolling subjects into this study. Blood samples will initially all be sent to the Breast Oncology Laboratory at the University of Michigan for testing until the first 32 subjects evaluable for analytical validity have had a baseline sample for CTC-ETI calculation (approximately the first 35-40 enrolled subjects). After confirmation of analytical validity of the first group of subjects, Mayo Clinic laboratories will also be utilized for the CTC-ETI testing. Study sites will be provided appropriate direction and materials regarding which lab to send specimens.

### 1.5 Study Population

Female subjects 18 years or older with ER positive, HER2 negative, progressive MBC after one or more lines of ET who are initiating a new ET will be enrolled into the study. Subjects must have immunohistochemistry (IHC) proven ER positive disease, IHC and/or fluorescence in-situ hybridization (FISH) proven HER2 negative disease, and an ECOG performance status of 0-2. Subjects with brain metastases only or those who are progressing on fulvestrant are not eligible for the study. All subjects must be informed of the investigational nature of this study and be willing to provide written informed consent in accordance with Institutional guidelines and Good Clinical Practice (GCP).

### 1.6 Sample Size

A sample size of approximately 200 subjects will provide a sufficient number of evaluable subjects and statistical power for evaluation of the primary and secondary objectives. It is estimated that a total of 120 subjects evaluable for clinical validity analyses will be required to obtain the minimum of 51 subjects with rapid progression for evaluation of the clinical validity of the CTC-ETI. We estimate that each site will enroll a total of between 10–30 subjects at a rate of 1–3 subjects per month, resulting in a study duration of approximately 2.5 years (1.5 years for enrollment, 1 year for follow-up). Enrollment will continue until a sufficient number of evaluable subjects to achieve statistical power for the evaluation of the primary objectives have been acquired. Subjects will be considered evaluable for clinical validity if they have successful calculation of a baseline CTC-ETI and progression status within 3 months of starting ET can be ascertained.

### 1.7 Methods

The sites will collect and ship the blood samples on the day of collection to the designated laboratory at ambient temperature via overnight courier using the tubes and shipping supplies provided by the Sponsor. At each blood draw ~40mL of blood will be obtained in 5 different 10mL CellSave Preservative Tubes (actual volume will be ~8mL of blood per tube). A minimum of 30mL of blood is required to complete the assays. Upon receipt in the laboratory, the blood will be pooled, and four separate 7.5mL aliquots will be evaluated for CTC + ER, CTC + Bcl-2, CTC + HER2, ,and CTC + Ki67 using the CELLSEARCH® CXC assay and marker reagents. If a patient has four cartridges containing 5 or more CTC, two will be fixed and stored frozen (-20°C) for future molecular analysis (i.e. FISH) and the other

Janssen Diagnostics, LLC
700 Route 202 South
Raritan, NJ 08869

2 will be stored in the refrigerator (4°C) for additional CTC-DNA analyses. If the patient consents, an optional, one tube of whole blood (~10 mL) will be collected into a Streck Cell-Free DNA Blood Collection Tube (or equivalent tube) for analysis of cell-free DNA. After completing cell-free DNA extraction, the product will be stored (either -20°C or -80°C) for future collaboration and analysis.

Subjects will also undergo serial body imaging as defined by their institution to follow disease outcomes. The protocol allows for PET/CT alone or CT scans of the chest/abdomen/pelvis alone. If the CT scans provide enough of an evaluation of bone metastases, a standard bone scan will be optional. The imaging studies that were performed at baseline (within 30 days prior to the initiation of ET) must be repeated 3 months after the initiation of therapy (within +/- 14 days) and when the patient is taken off study (i.e. maximum of 12 months after the initiation of therapy, at the time of disease progression, or at the time of discontinuation of treatment, whichever occurs first, using the same imaging modalities employed at baseline to assess the selected target and/or non-target lesions (RECIST v1.1 guidelines). Any other imaging performed during the course of the study will be at the discretion of the managing physician. At each CTC-ETI blood draw and/or follow-up evaluation, the physician's overall impression (or their designee, such as nurse practitioner or physician's assistant) of the subject's clinical status, as well as the results of any serum tumor marker testing that is done, will be recorded. The study schema below illustrates all planned study interventions:

PRODUCTS ARE FOR RESEARCH USE ONLY AND THEIR PERFORMANCE CHARACTERISTICS ON CLINICAL SAMPLES HAVE NOT BEEN DETERMINED

Janssen Diagnostics, LLC
700 Route 202 South
Raritan, NJ 08869

***STUDY SCHEMA***

| Eligibility: ER positive, Her2 negative, progressive MBC starting new Endocrine Therapy | BL [1] | 1 mo [2] | 2 mo [2] | 3 mo [2] | 6 mo [2] | 9 mo [2] | Off Study / 12 mo [2,3] |
|---|---|---|---|---|---|---|---|
| Medical Record Review / History | X | | | | | | |
| Physician (or Designee): Assessment of Clinical Status | X | X | X | X | X | X | X |
| Blood draw: CTC-ETI + optional cfDNA | X | X | X | X | | | X |
| Imaging Assessments: Refer to Section 8.6 | X | | | X | ← Physician Discretion | → | X |
| Optional: Provision of Archival Tissue Specimens (blocks or slides) | X | | | | | | |

*Abbreviations: BL=baseline ; mo = month*

[1] *__Baseline Assessments__: Must be done within 30 days prior to initiation of new endocrine therapy.*

[2] *__Follow-up Assessments__: Must be performed +/- 14 days of designated time point. Frequency of clinical follow-up is every 3 months. Imaging between 3-12 months is at the discretion of the managing physician, however, all target and/or non-target lesions selected at baseline must be assessed at 3 months and at progression/off study/12 months with the same imaging modality used at baseline (RECIST v1.1 guidelines).*

[3] *__12 Month or Off Study Assessments__: Must be done at the time the patient is taken off the study (i.e. at disease progression, discontinuation of therapy, or 12 months after the initiation of therapy without disease progression, whichever occurs first). All target and/or non-target lesions selected at baseline must be assessed with the same imaging modality used at baseline (RECIST v1.1 guidelines).*

For each evaluable subject, we will also attempt to obtain archived tumor specimens, if available, from the primary and metastatic cancers (either as tissue blocks or tissue sections on slides, in that order of importance). The tissue will be analyzed in a single laboratory using standard IHC for ER, Bcl-2, HER2 and Ki67. HER2 will also be analyzed with FISH.

Clinical and laboratory data will be collected from the subject's medical records / clinic chart using paper case report forms (CRFs). The completed CRFs will be sent to the Sponsor for input into a database to be used for the analyses. CTC-ETI results (i.e. CTC counts, with average and relative Assigned CTC Points, proportion of CTC positive for each marker, CTC-ETI Bio-Points and Bio-Score, final CTC-ETI Score and relative Category) from the study laboratory(ies) will also be captured and input into the database. The data will be analyzed according to the statistical analysis plan. **The CTC-ETI results (i.e. CTC counts and/or biomarker results) will not be provided to the subjects and/or their physician.** However, the study physician will be notified by the Sponsor when a subject has an unsuccessful baseline CTC-ETI calculation, and these subjects will be notified by the physician and/or site study staff that they will be removed from further participation in the

Janssen Diagnostics, LLC
700 Route 202 South
Raritan, NJ 08869

study. The study laboratory(ies) will be blinded to subjects' clinical data. The imaging studies and physician assessments outlined in the study schema are considered part of routine clinical care while the medical record review / history, the blood draws for CTC-ETI testing, and the optional provision of archival tissue specimens are being done specifically for the purposes of this research study.

## 2.0 ABBREVIATIONS AND DEFINITIONS

| | |
|---|---|
| APC | Allophycocyanin |
| Bcl-2 | B-cell lymphoma 2 |
| BMC | BioMarker Cartridge |
| CA15-3 | Cancer Antigen 15-3 |
| CA27.29 | Cancer Antigen 27.29 |
| CEA | Carcinoembryonic antigen |
| cfDNA | Cell-Free DNA |
| CNS | Central Nervous System |
| CRF | Case Report Form |
| CT | Computed tomography |
| CTC | Circulating Tumor Cell |
| DAPI | 6-diamidino-2-phenylindole, dihydrochloride |
| DCF | Data Correction Form |
| DIOC | Dihexyloxacarboeyanine Iodide |
| EpCAM | Epithelial Cell Adhesion Molecule |
| ER | Estrogen Receptor |
| ET | Endocrine Therapy |
| ETI | Endocrine Therapy Index |
| FDA | Food & Drug Administration |
| FISH | Fluorescence In-Situ Hybridization |
| FLU | Fluorescein isothiocyanate |
| GCP | Good Clinical Practice |
| HER2 | Human Epidermal Growth Factor Receptor 2 |
| ICH | International Conference on Harmonisation |
| IEC/IRB | Independent Ethics Committee / Institutional Review Board |
| IHC | Immunohistochemistry |
| MAb | Monoclonal Antibody |
| MBC | Metastatic Breast Cancer |
| mL | Milliliter |
| MRI | Magnetic Resonance Imaging |
| OS | Overall Survival |

Janssen Diagnostics, LLC
700 Route 202 South
Raritan, NJ 08869

| | |
|---|---|
| PE | Phycoerythrin |
| PET | Positron Emission Tomography |
| PFS | Progression Free Survival |
| QC | Quality Control |
| RECIST | Response Evaluation Criteria in Solid Tumors |
| RUO | Research Use Only |
| WB | Whole Blood |

# 3.0 INTRODUCTION AND STUDY RATIONALE

Breast cancer can be treated with a variety of ET approaches, which can basically be divided into additive and ablative [1]. Selection of appropriate treatment for patients with MBC is based on two important factors: prognosis and prediction. MBC patients with ER negative breast cancer have almost no chance of benefit from anti-estrogen (or “endocrine”) therapies, and are therefore treated with more toxic, but more likely beneficial, chemotherapy [2]. Patients with ER positive MBC initiating first line ET have only a 30-50% chance of receiving clinical benefit [1,2]. For the other 50-70%, ET is ineffective and these patients should probably be treated with chemotherapy, as is done for ER negative patients. More importantly, in nearly every clinical trial of ET in ER positive, MBC patients, between 15-30% of enrolled subjects progress in the first 2-3 months, regardless of whether they are receiving first or later lines of ET [3-6]. Currently there is no validated method to identify which ER positive MBC patients will be refractory to ET. Therefore, almost all ER positive patients are treated with serial endocrine therapies prior to switching to chemotherapy. We propose that a subset of these patients would be better served with chemotherapy, in spite of its increased toxicity profile, rather than delaying chemotherapy during a several month trial of ineffective, albeit less toxic, ET.

One method of identifying patients who are refractory to ET would be to perform a biopsy of their metastases at each clinical juncture. However, this strategy is impractical, unsafe, and costly. We hypothesize that evaluation of CTC might serve as a “real time biopsy” that can be performed easily, safely, and relatively inexpensively. Many technologies have been developed to isolate, enumerate, and characterize CTC [7], but the CELLSEARCH® system (Veridex, LLC) is the only FDA-approved or cleared assay to do so. Consequently, it has become the most widely adopted CTC assay in clinical care. Enumeration of CTC using the CELLSEARCH® System has been shown to be a sensitive, independent predictor of PFS and OS in patients with metastatic breast, colon and prostate cancer [8-10]. We hypothesize that in addition to enumerating CTC, characterization of CTC might provide biologic insight into their behavior.

Utilizing CELLSEARCH® technology, the University of Michigan Comprehensive Cancer Center conducted pre-clinical and pilot clinical studies [11,12] that demonstrated the ability to both enumerate and reliably and reproducibly characterize CTC for a number of tumor markers, including those that predict for either endocrine sensitivity (ER [13,14] and Bcl-2 [15-17]) or resistance (HER2 [18-20] and Ki67 [21-24]). For the most part, these markers have been assayed in tissue taken from the primary cancer, although on occasion they are measured in the

Janssen Diagnostics, LLC
700 Route 202 South
Raritan, NJ 08869

metastases. Importantly, several of these markers are incorporated in the multi-parameter 21-gene RT-PCR-based assay (Oncotype DX® Genomic Health Inc; Redwood City, CA), which is functional in formalin-fixed, paraffin-embedded breast cancer tissue. Relative values of ER, HER2, Bcl-2, and Ki67 expression are primary drivers in the algorithm used to derive the Oncotype DX® "Recurrence Score". Highly validated correlative studies have demonstrated that both node negative and node positive patients with low recurrence scores, reflecting high ER and Bcl-2, and low HER2 and Ki67, have an extraordinarily favorable prognosis when treated with tamoxifen alone. Those with high recurrence scores, reflecting low ER and Bcl-2 and high HER2 and Ki67, have a much worse prognosis when treated with tamoxifen alone, and additionally, are much more likely to benefit from chemotherapy [17, 25-26].

Unlike development of new drugs, research of cancer biomarkers has been chaotic, in part due to poor systematic "rules" and semantics on how best to generate data to determine if a new marker should be used to direct care [27]. Recently, the Centers for Disease Control has convened the Evaluation of Genomic Applications in Practice and Prevention (EGAPP) initiative [28], which has provided three definitions to articulate necessary components of tumor biomarker research:

- *Analytical Validity*: Defined as reliable, accurate, and reproducible assay development. Analytical validity is essential to apply a biomarker assay to clinical care.
- *Clinical/Biological Validity*: Defined as evidence that the biomarker divides a single population into two groups with statistically significant biologic or clinical outcomes differences (such as progression free or overall survival). Clinical validity may or may not be of use in caring for patients, but in general if a tumor biomarker does not have clinical validity, it almost certainly will not have clinical utility.
- *Clinical Utility*: Defined as the presence of high levels of evidence that the use of a tumor biomarker lead to better outcomes compared to not using the marker, and therefore should be used to manage a patient's care.

**In the clinical trial proposed in this protocol, we plan to address the first two of these issues for a newly developed assay: the CTC-ETI.** In order to predict benefit from or resistance to ET, we have created an index (the CTC-ETI) that takes into account the number of CTC (which is prognostic) as well as the phenotype of the CTC, based on our hypothesis that relative levels of ER and Bcl-2 (high=benefit) and HER2 and Ki67 (high=resistance) are predictive of ET responsiveness or resistance.

In the pilot study at the University of Michigan, 28 of 40 patients enrolled had ≥5 CTC/7.5mL of whole blood (WB), and a CTC-ETI was successfully determined in 22 patients (79% of patients with elevated CTC). Expression of the biomarkers among CTC in single patients was heterogeneous, suggesting that future iterations of the CTC-ETI will have to consider expression variability. Blinded determination of CTC-ETI demonstrated high inter-observer concordance, as shown in the table below.

CTC-ETI Concordance between 2 reviewers

| Marker | Concordance | Kappa (95% Conf. Limits) | CTC–ETI Important Discordance* |
|---|---|---|---|

PRODUCTS ARE FOR RESEARCH USE ONLY AND THEIR PERFORMANCE CHARACTERISTICS ON CLINICAL SAMPLES HAVE NOT BEEN DETERMINED

Janssen Diagnostics, LLC
700 Route 202 South
Raritan, NJ 08869

| | | | |
|---|---|---|---|
| ER | 94% | 0.91 (0.89-0.92) | 3% |
| Bcl-2 | 95% | 0.92 (0.91-0.94) | 2% |
| HER2 | 95% | 0.89 (0.88-0.92) | 2% |
| Ki-67 | 96% | 0.94 (0.92-0.95) | 1% |

*"Important discordance" = would have changed CTC-ETI category for patient: CTC-ETI was calculated after resolution of discordance between observers

Based on the results of the pre-clinical and pilot clinical studies performed at the University of Michigan Comprehensive Cancer Center, an algorithm for a CTC-ETI that can be calculated for each subject was generated (described in Section 5.2 below).

The primary goal of the current study is to determine a CTC-ETI in ER positive, HER2 negative MBC subjects before the initiation of a new ET that can identify those subjects that will progress rapidly.

In summary, selection of appropriate therapy is critical to ensure optimal palliation, if not prolonged survival, in patients with MBC. The clinician would prefer to achieve response with relatively non-toxic ET, but one would prefer not to expose a patient to ineffective therapy for several months if they stand a considerably higher chance of responding to chemotherapy. While lack of ER is a highly accurate indicator that ET will not work, and therefore chemotherapy is preferable, no currently existing marker is sufficient to withhold a trial of ET from ER positive MBC patients. However, only approximately 30-50% of ER positive patients will benefit from anti-estrogen treatment. In these patients, the 21 gene recurrence score could be performed on metastatic tissue, but to do so requires invasive, aggressive, and sometimes dangerous re-biopsy procedures. We hypothesize that baseline and serial CTC-ETI scores will provide a similar biological "snapshot" that can be done easily with minimal invasion, and thus might permit rapid identification of subjects for whom ET is unlikely to be of value. Although the preliminary data demonstrate our ability to detect, enumerate, and characterize CTC, the purpose of the current study is to establish proof of principle that these 4 markers can be used to generate a CTC-ETI which can be performed at baseline from subjects enrolled at different centers, and that baseline CTC-ETI predicts relative outcome for subjects with ER positive MBC starting a new ET, and can be monitored in such subjects during ET. Successful completion of this study will set the stage for a larger, definitive study designed to demonstrate the clinical utility of a "refined" CTC-ETI in subjects with ER positive, HER2 negative MBC.

<u>Rationale for Amendment 1 dated January 14, 2013</u>: After Investigator review of the original protocol dated September 21, 2012, conducted at a group meeting held on December 5, 2012, it was agreed by the majority that the definition of standard of care with regard to tumor markers and imaging studies varied among institutions. Based on this concern, the amended protocol dated January 14, 2013 deletes the requirement for the collection of serum tumor markers and the standardization of imaging at the 6 and 9 month follow-up time points. The imaging language and schema were modified to indicate that the imaging between 3 and 12 months would be done "at the discretion of the physician". However, all assessments must still be done 3 months after the initiation of therapy (within +/- 7 days) and when the patient is taken off study (i.e. maximum of 12 months after the initiation of therapy or at the time of progression, whichever occurs first), using the same imaging modalities employed at baseline (RECIST v1.1 guidelines). This is to ensure an adequate assessment of rapid progression and

progression free survival. These changes were made to avoid absolute requirement of continued bone scans and other scans that might not be required for following the target lesions or that would not be covered by insurance carriers and/or considered "standard of care" by the various investigators and institutions. None of these changes impact the primary objectives or the sample size.

Rationale for Amendment 2 dated April 3, 2013: Following additional discussions with several investigators, the study PI, and the study statistician, it was decided that the imaging windows at baseline and the follow-up time points should be expanded. At baseline, the timeframe was extended from 21 days prior to therapy to 30 days prior to therapy. At the follow-up time points, the timeframe was extended from +/- 7 days to +/- 14 days of the specified time point. The types of imaging assessments expected were also clarified further. The main reason for these changes was to bring the study imaging requirements closer to the current "standard of care" at the various institutions. None of these changes impact the primary objectives or sample size.

Rationale for Amendment 3 dated December 22, 2014: This amendment is being written because new technologies and assays are being developed and we would like to request permission for further analysis on the CellSearch enriched CTCs. We are asking permission to send specimens collected during the conduct of this study to outside institutions or collaborators in order to sequence DNA of the CTCs captured by the CellSearch technique. The existing informed consent has been revised to reflect this request and an addendum informed consent has been drafted for those patients who are already off of the study. The study sites will make efforts to collect consent from the off study patients. Additionally, the protocol excludes any subject exposed to a Category A infectious substance, clarifies the protocol-required washout period for fulvestrant, adds an optional collection of blood for cfDNA analysis, incorporates GINA language for future additional molecular analyses, confirms that Mayo Clinic will be the second testing laboratory for the CTC-ETI, and allows for the Investigator to follow each patients disease at his/her discretion provided the imaging performed at baseline is the same imaging method use for the follow-up imaging time points.

# 4.0 STUDY OBJECTIVES AND DURATION

## 4.1 STUDY OBJECTIVES

The primary objectives of this study are to:

- Demonstrate that the CTC-ETI can be accurately determined at initiation of a new ET (baseline) in subjects with ER positive, HER2 negative MBC in multiple centers across North America (analytical validity [27]);
- Determine if the current CTC-ETI algorithm is associated with rapid progression, defined as progression within three (3) months of starting a new ET in subjects with ER positive, HER2 negative MBC (clinical validity [27]);
- Refine the current CTC-ETI algorithm, if necessary, so that it accurately predicts rapid progression in subjects with ER positive, HER2 negative MBC starting a new ET.

The secondary objectives of this study are to:

Janssen Diagnostics, LLC
700 Route 202 South
Raritan, NJ 08869

- Determine if a CTC-ETI can be accurately determined at serial time points during treatment in subjects with ER positive MBC after starting a new ET in multiple centers across North America.
- Determine if the current/refined CTC-ETI algorithm is associated with PFS in subjects with ER positive, HER2 negative MBC after starting a new ET who have been followed for up to 12 months.
- Determine changes in CTC-ETI during ET and see if these changes are associated with outcomes (i.e. rapid progression, PFS).
- Correlate the status of the biomarkers on baseline CTC with the status of the same biomarkers in primary and/or metastatic tissue collected from the subjects.
- Store CTC positive cassettes for future molecular analyses.
- Store cfDNA for future analyses.

### 4.2 STUDY DURATION

The subject accrual for this study is expected to begin sometime in early Q2 2013. We estimate that each site will enroll a total of between 10–30 subjects at a rate of 1–3 subjects per month, resulting in a study duration of approximately 2.5 years (1.5 years for enrollment, 1 year for follow-up). Subjects will have blood drawn for CTC-ETI calculation at baseline (within 30 days prior to the initiation of ET) and then subsequently 1, 2, 3 and up 12 months after the initiation of therapy (within +/- 14 days of the specified time point), or at the time of disease progression, whichever occurs first. **Only subjects with a successful baseline CTC-ETI calculation (see Appendix E) will remain on study and have subsequent serial blood draws performed.**

At pre-determined subject accrual points (i.e. after approximately 35–40 subjects have been enrolled and again after approximately 70–80 subjects have been enrolled) we will assess the baseline CTC-ETI testing for analytical validity and the study design for futility (see Section 12.8). If the criteria for futility are met, the study may be stopped early. Upon completion of the enrollment (if the trial has not been stopped for futility reasons) and after a minimum of 3 months of follow-up for all evaluable subjects, the CTC-ETI algorithm will be assessed and refined to identify subjects who will experience rapid progression with high specificity (low false-positive fraction). The refined CTC-ETI algorithm will be validated in a future study designed for submission to the FDA to establish its positive predictive value.

## 5.0 DEVICE DESCRIPTION AND STUDY SUPPLIES

### 5.1 ASSAY/DEVICE DESCRIPTIONS

**All assays performed on the blood collected specifically for the purposes of this study are for research use only and the results may not be used for patient management.** The RUO CELLSEARCH® CXC Kit enables the immunomagnetic selection of CTC of epithelial origin from WB and the characterization of user-defined markers with low antigen density (~50,000 antigens/cell). CTC are isolated from a 7.5mL sample of WB using the reagents in

Janssen Diagnostics, LLC
700 Route 202 South
Raritan, NJ 08869

the CELLSEARCH® CXC Kit and a CELLTRACKS® AUTOPREP® System and subsequently identified using the CELLTRACKS ANALYZER II®. CTC are defined as Epithelial Cell Adhesion Molecule (EpCAM) positive, cytokeratin positive, 4', 6-diamidino-2-phenylindole dihydrochloride (DAPI) positive, CD45 negative events with a cellular morphology including an intact cytoplasmic diameter of at least 4µm containing a nucleus.

Before placing samples on the CELTRACKS® AUTOPREP® System for processing, blood is pooled, aliquoted into separate CELLTRACKS® AUTOPREP® sample tubes, buffer is added, and the samples are centrifuged (see CXC Kit Instructions for Use [IFU] for detailed procedure). After incubation and magnetic separation with magnetic nano-particles (ferrofluid) labeled with antibodies specific for EpCAM, the cell suspension enriched for cells expressing EpCAM is incubated with fluorescein isothiocyanate (FLU) labeled monoclonal antibodies recognizing cytokeratins 8, 18 and/or 19 (markers of epithelial cells) [CK-FLU], allophycocyanin (APC) labeled monoclonal antibodies specific for CD45 (a broad-spectrum leukocyte marker) [CD45-APC], the nucleic acid dye DAPI, and the selected PE conjugated tumor profiling antibody in the presence of the staining buffer. Excess fluorescent material is removed by repeated magnetic washes, and the fluorescently labeled cells are resuspended in a cellular preservative to a final volume of 320µL and transferred to a cell presentation chamber (cartridge). This cartridge is held in a magnetic device called a MAGNEST® Cartridge Holder, which orients the magnetically labeled cells for fluorescence microscopic examination in the CELLTRACKS ANALYZER II®. This semi-automated image analysis system identifies and captures images of the fluorescently labeled cells and presents them to the operator for classification and enumeration. One CXC control is run on the instrument each day. Positive and negative controls for each marker will be run with each batch of samples.

#### 5.1.1 CXC Assay

The CELLSEARCH® CXC Kit contains EpCAM labeled ferrofluid, CK-FLU, CD45-APC, DAPI and a staining buffer. The following tumor profiling antibodies, conjugated to PE, will be used to characterize the isolated CTC: ER, HER2, Bcl-2, Ki67.

#### 5.1.2 CXC Assay Controls

The CELLSEARCH® CXC Control Cell Kit contains 24 single-use bottles containing a fixed breast cancer cell line (SK-BR-3) pre-labeled with fluorescent dyes. A CELLSEARCH® CXC Control Cell bottle is used to verify the performance of the CELLSEARCH® CXC Kit reagents, sample processing by the CELLTRACKS® AUTOPREP® System, and cell analysis by the CELLTRACKS ANALYZER II®. The fixed SK-BR-3 cells are a single level population and differentiated as control cells by the detection of fluorescence in the dihexyloxacarboeyanine iodide (DIOC) control channel of the CELLTRACKS ANALYZER II®. The number of SK-BR-3 cells isolated is automatically tallied by the system and must fall within a pre-determined range specific to that particular lot of control cells. One CXC control is run per instrument per day.

Janssen Diagnostics, LLC
700 Route 202 South
Raritan, NJ 08869

### 5.1.3 Marker Controls

Positive and negative controls for the tumor profiling antibodies consist of the appropriate cell line(s) positive and/or negative for the marker being evaluated. Normal blood or buffer spiked with the negative cell lines and processed in the presence of the marker reagent (negative control) is used to establish the background signal in the PE channel. The normal blood or buffer spiked with the positive cell lines and processed in the presence of the marker reagent (positive control) is used to control the marker reagent. Each cell will be evaluated for positive staining of the marker (i.e. staining above the negative control), and the proportion of cells positive for each of the markers will be determined. The table below indicates the cell lines and their marker reactivity.

| Control Cell Line | Marker Positive | Marker Negative |
|---|---|---|
| MCF-7 | **ER**, Bcl-2, Ki67 | **HER2** |
| SK-BR-3 | **HER2**, Ki67 | **ER**, **Bcl-2** |
| BT-474 | ER, **Bcl-2**, HER2, Ki67 | **---** |

To reduce the number of marker controls that have to be run, it may be possible to spike two cell lines into a single aliquot of blood or buffer to control for two different markers at once. For example, the MCF-7 and SK-BR-3 cell lines can be combined into a single aliquot to act as the positive and negative controls, respectively for ER (nuclear localization) and the negative and positive controls, respectively, for HER2 (cytoplasmic localization). Similarly, the SK-BR-3 and BT-474 cell lines can be combined into a single aliquot to act as the negative and positive controls, respectively, for Bcl-2 (cytoplasmic localization). All of the cell lines are positive at some level for Ki67 (nuclear localization), however, there should always be a population of cells that are not undergoing proliferation and thus you will always have a population of cells that will be negative for Ki67.

### 5.1.4 BioMarQ Software

We have also developed RUO software for the CELLTRACKS ANALYZER II® that allows quantitation of the marker expressions. The software, called BioMarQ, is an enhancement to the CELLTRACKS ANALYZER II® platform that allows for quantitative analysis of Tumor Profile Reagents (i.e. markers). BioMarQ is for research use only and consists of a new standardization cartridge (called a BioMarker Cartridge, or BMC) and an external hard drive loaded with a modified version of the CELLTRACKS ANALYER II® software. Before scanning the samples or controls on the CELLTRACKS ANALYZER II®, the BMC is scanned and the images are analyzed. Next, the blood samples and positive/negative controls are loaded and a test definition is selected with the associated marker definition. Immediately prior to scanning the sample or control, camera settings for the marker channels are adjusted using the BMC test results. After the images captured from the samples and/or controls are classified in the browser, image clips are generated from the collected raw images. These image clips are used for segmentation of the CTC; images of selected CTC can be either saved to disk or printed. A series of features, including marker intensity, are extracted from the segmented cells and these features are saved to a disk. The extracted features can be

analyzed using scatter plots and histograms and the results and CTC image related to selected data points can be visualized on the screen.

Primary correlation with rapid progression and PFS in this trial will be made using CTC-ETI calculation derived from the visual determination of the biomarker expression on the CTC and not from the BioMarQ data. We will use the data collected from the BioMarQ software in conjunction with the operator's visual interpretations of the markers to help determine marker intensity thresholds that could possibly be used in future studies to classify CTC images as marker positive or marker negative.

### 5.2 Calculation of CTC-ETI

For each blood draw (five tubes of WB, ~40-50mL total), the WB collected will be pooled and four separate 7.5mL aliquots will be prepared. The remaining WB will be saved to be used as a backup for retesting if necessary. Upon completion of the testing, all used and unused WB (and tubes) will be appropriately disposed of.

Each marker will be evaluated in a separate 7.5mL aliquot of WB. CTC will be enumerated in each of the four different 7.5mL aliquots of WB, and the average CTC count of the four tubes will be calculated. **Table 1** below provides the CTC Assigned Points for each category of average CTC levels.

***Table 1. CTC Assigned Points: Prognosis Based on Average CTC Number Only***

| | **Low (Good)** | **Intermediate** | | **High (Bad)** |
|---|---|---|---|---|
| ***Number of CTC/7.5mL (average of 4 aliquots)*** | 0-4 | 5-10 | 11-100 | > 100 |
| ***Assigned Points*** | 0 | 1 | 3 | 4 |

Next, for those subjects who have an average of 5 or more CTC/7.5mL of WB (average of CTC values from the 4 aliquots), we assign "Biologic Points" (or Bio-Points) based on the percentage of CTC that are positive for the respective marker (**Table 2**). Note that we have weighed low or negative ER expression more heavily than HER2, Ki67, or Bcl-2 due to its fundamental role in endocrine responsiveness. These Bio-Points are added to calculate a final CTC Bio-Score (NOTE: this score is currently for informational purposes only and may not be used in the management of patients).

***Table 2. CTC Assigned Biologic Points: Prediction of Endocrine Sensitivity Based on Relative Expression of Each Biological Marker Within CTC (elevated CTC ≥ 5/7.5mL of WB only).***

| **Response Prediction to ET** | **Favorable** | | **Intermediate** | | **Unfavorable** | |
|---|---|---|---|---|---|---|
| | *% CTC positive* | *Assigned Bio-Points* | *% CTC positive* | *Assigned Bio-Points* | *% CTC positive* | *Assigned Bio-Points* |
| ***ER*** | >10% | 0 | 1-10%* | 2 | 0%* | 6 |
| ***Bcl-2*** | >10% | 0 | 1-10% | 1 | 0% | 2 |
| ***HER2*** | 0% | 0 | 1-10% | 1 | >10% | 2 |
| ***Ki67*** | 0% | 0 | 1-10% | 1 | >10% | 2 |

*NOTE: we have weighed low or negative ER expression more than HER2, Ki67, or Bcl-2, due to its fundamental role in endocrine responsiveness. Thus final CTC Bio-Score can range from 0 (ER and Bcl-2 high; HER2 and Ki67neg) to 12 (ER and Bcl-2 neg; HER2 and Ki67 high). Abbreviation: ET= Endocrine Therapy

Finally, we add the CTC Assigned Points and CTC Bio-Score (defined as the combination of CTC Assigned Biologic Points for each marker) to derive the CTC-ETI Score (**Table 3**).

***Table 3. CTC-ETI Scores***

| **Average CTC Counts** | **CTC Assigned Points** | **Marker** | **CTC Assigned Biologic Points** | | | **Potential CTC-ETI Score (sum of CTC Assigned and Bio- Points)** |
|---|---|---|---|---|---|---|
| | | | *Low* | *Intermediate* | *High* | |
| ***CTC 0-4*** | 0 | N/A | 0 | 0 | 0 | 0 |
| ***CTC 5-10*** | 1 | ER | 0 | 2 | 6 | 1-13 |
| | | Bcl-2 | 0 | 1 | 2 | |
| | | HER2 | 0 | 1 | 2 | |
| | | Ki67 | 0 | 1 | 2 | |
| ***CTC 11-100*** | 3 | ER | 0 | 2 | 6 | 3-15 |
| | | Bcl-2 | 0 | 1 | 2 | |
| | | HER2 | 0 | 1 | 2 | |
| | | Ki67 | 0 | 1 | 2 | |
| ***CTC > 100*** | 4 | ER | 0 | 2 | 6 | 4-16 |
| | | Bcl-2 | 0 | 1 | 2 | |
| | | HER2 | 0 | 1 | 2 | |
| | | Ki67 | 0 | 1 | 2 | |

Each of the factors (CTC, ER, HER2, Ki67, and Bcl-2) were placed into categories to develop the CTC-ETI by combining the number of CTC (CTC Assigned Points) and the Biologic Points of these CTC (CTC Bio-Score), as determined using the CELLSEARCH® CXC assay and marker reagents. A subject can have a CTC-ETI Score ranging from 0 (0-4 CTC/7.5mL WB), or 1 (5-10 CTC/7.5mL WB, but all Bio-Scores are 0) to 16 (>100 CTC/7.5mL WB, 0% of these cells are positive for ER and Bcl-2, and >10% of CTC are positive for HER2 and Ki67).

To make the CTC-ETI Score clinically applicable, the scores are placed into 3 categories, much as histologic grading is categorized. We hypothesize that a Low CTC-ETI will predict a favorable outcome (response, long time to progression) for subjects with ER positive MBC starting a new ET, while a High CTC-ETI will predict rapid progression. Intermediate CTC-ETI scores should fall in between the other two. These proposed categories are provided in

**Table 4**.

***Table 4. Proposed CTC-ETI Categories***

| *CTC-ETI Category* | CTC-ETI Score | Predictive clinical outcomes | Treatment strategy |
|---|---|---|---|
| ***Low*** | 0-3 | Favorable; respond to ET and/or indolent disease; long time to progression | Treat with ET* |
| ***Intermediate*** | 4-6 | Probably respond or moderately indolent disease; modest time to progression | Treat with ET* |
| ***High*** | 7-16 | Poor; Resistant to ET, rapid progression | Treat as ER neg with chemotherapy |

Abbreviations: *ET= Endocrine Therapy.

(NOTE: The proposed CTC-ETI Score and Categories are for informational purposes only and may not be used in the management of patients).

## 5.3 Study Supplies, Storage, Use and Accountability

### 5.3.1 Clinical Sites

The Sponsor will provide all participating clinical sites that are enrolling subjects with the following study supplies: CellSave Preservative tubes, Streck cfDNA blood collection tubes, insulated shippers (which consists of an insulated cooler, a plastic canister, an absorbent pouch, a ZipLock bag, and two different gel/water packs), pre-printed FedEx airway bills for shipment of the blood samples to the study laboratory (**Appendix C**), and CRFs (**Appendix B**). The tubes and shippers must be stored at ambient temperature, and the gel/water packs must NOT be refrigerated or frozen prior to use. The supplies provided by the Sponsor are to be used only for the conduct of this study. Sponsor will keep a record of all study supplies provided to each clinical site for the conduct of this study. Upon completion of the study, all unused study supplies will either be returned to the Sponsor (at the expense of the Sponsor) or destroyed and appropriate documentation of the destruction of each item provided to the Sponsor.

### 5.3.2 Study Laboratory Sites

The Sponsor will provide all participating study laboratory sites that are processing specimens with the following study supplies: CELLSEARCH® CXC Kits, CELLSEARCH® CXC Control Cell Kits, CELLTRACKS® AUTOPREP® System buffer, PE labeled marker reagents for HER2, ER, Bcl-2 and Ki67, fixed cell lines for control of markers (to be spiked into normal donor blood), CellSave Preservative Tubes for collection of blood from healthy volunteers (to be used for collection of blood for spiking marker controls, if required), appropriate CRFs for results reporting, notebooks for

enrollment and accession logs, control results, etc. (**Appendix B**). All study laboratory sites will also be provided with a research instrument (called a Neon), to be used for the fixation and drying of CTC positive cartridges, which will need to be stored at -20$^{o}$C for future molecular analyses (i.e. FISH). The other two positive cartridges will be stored in the refrigerator (4° C) for additional analyses. All kits and reagents must be stored according to the manufacturer's instructions or specific instructions provided with the reagents. Sponsor will keep a record of all study supplies provided to each study laboratory for the conduct of this study. Upon completion of the study, all unused study supplies will either be returned to the Sponsor (at the expense of the Sponsor) or destroyed and appropriate documentation of the destruction of each item provided to the Sponsor.

# 6.0 SUBJECT SELECTION

The inclusion and exclusion criteria for enrolling subjects in this study are described below. If there is a question about the inclusion or exclusion criteria below, the investigator should consult with the primary Sponsor contact before enrolling a subject in the study. For a detailed discussion of the statistical considerations for the sample size, refer to Section 12, Statistical Considerations.

## 6.1 INCLUSION CRITERIA

Female subjects meeting all of the following criteria will be considered for enrollment into the study:

- All subjects must be informed of the investigational nature of this study and be willing to provide written informed consent (**Appendix A**) in accordance with Institutional guidelines and GCP indicating that they understand the purpose of and procedures required for the study and are willing to participate in the study.
- Women who are 18 years or older.
- Subjects must have metastatic breast cancer (i.e. primary tumor histopathology and imaging showing distant sites of metastasis or biopsy-proven metastatic lesions) that is either measurable or non-measurable according to RECIST v1.1 with at least one non-irradiated distant site of metastasis (non-local metastases are required) [29].
- Subjects with an ECOG performance status of 0-2 [31].
- Subjects must have IHC-determined ER positive breast cancer according to institutional guidelines (although evaluation of metastatic lesions is preferred, tissue evaluated for ER can be from primary tumor or metastatic lesions).
- Subjects must have HER2 negative breast cancer defined by CAP/ASCO criteria (IHC 0-1; IHC 2 & FISH ratio $\leq$2.0 or average of HER2 gene copy number of <4 signals/nucleus for test systems without an internal probe; or FISH ratio <2.0 or average of HER2 gene copy number of <4 signals/nucleus for test systems without an internal probe) [30] (although evaluation of metastatic lesions is preferred, tissue evaluated for HER2 can be from primary tumor or metastatic lesions).

*PRODUCTS ARE FOR RESEARCH USE ONLY AND THEIR PERFORMANCE CHARACTERISTICS ON CLINICAL SAMPLES HAVE NOT BEEN DETERMINED*

Janssen Diagnostics, LLC
700 Route 202 South
Raritan, NJ 08869

- Subjects must have currently progressive metastatic disease according to RECIST v1.1 criteria which may include objective criteria for progression based on physical findings and/or whole body anatomic and/or bone scintigraphic imaging, AND
    - o They have progressed on at least one previous line of ET for their metastatic disease (but are not currently progressing on fulvestrant or have had a Fulvestrant washout of at least 120 days– see Exclusion Criteria below), OR;
  - o They show evidence of disease progression during or within 12 months of the end of adjuvant ET.
- Subjects are about to start a new line of ET for their metastatic disease (may be enrolled regardless of prior surgery, radiation, chemotherapy, or investigational therapy, as long as they have at least one identifiable metastatic lesion that has not been irradiated).
- Subject is willing and able to undergo standard of care imaging studies (same imaging/staging modality being used at each evaluation), which are anticipated to be performed prior to the initiation of therapy and subsequently every 3 months.
- Subject agrees to the collection and testing of their blood and is willing and able to provide approximately 40mL blood draw(s) at:
  - o Baseline (prior to the initiaton of new ET), and;
  - o Subsequently at 1, 2, 3 and 12 months after the initiaton of therapy, and/or;
  - o Time of disease progression or discontinuation of treatment.

### 6.2 Exclusion Criteria

Any subject who meets any of the following criteria will be excluded from participating in the study:

- Male subjects.
- Subjects who are concurrently participating in a therapeutic clinical trial addressing ET plus or versus an additional investigational treatment.
- Subjects with local regional recurrence only or brain only metastasis (subjects with Central Nervous System (CNS) metastases are eligible if they also have progression of a metastatic disease elsewhere, and the CNS metastases are stable for 3 months or more after the completion of CNS therapy (surgery, radiation)).
- Subjects who are progressing on current fulvestrant therapy (subjects who have had fulvestrant therapy in the past and were subsequently treated with other therapies or those who are starting fulvestrant as their next line of ET are eligible for the study). If a potential study patient has previously been treated with fulvestrant, the wash out period must be at least 120 days since the last dose (this represents three times the half-life of the drug) until the patient can be eligible fot this study. NOTE: Since fulvestrant downregulates ER, it is likely that fulvestrant therapy will artificially induce a very high CTC-ETI, since most CTC would be expected to be ER negative. It is possible

that CTC might re-upregulate ER as they progress on fulvestrant, but we are unaware if this is occurs or not. Therefore, patients who are progressing on fulvestrant just prior to being considered for this study are not eligible.

- Subjects who are or will be taking other unapproved (i.e. not cleared/approved by the FDA) anti-neoplastic therapies concurrently are not eligible (exception: ET with everolimus is acceptable).
- Subjects with concomitant malignancies or previous malignancies within the last 5 years, with exception of adequately treated basal or squamous cell carcinoma of the skin or carcinoma in situ of the cervix.
- Unable to provide informed consent or high risk that subject may not comply with protocol requirements (i.e. due to health and/or participation in other research studies).

# 7.0 STUDY LABORATORY TRAINING AND PROFICIENCY TESTING

Testing of the blood samples for CTC-ETI calculation will be done only at selected laboratories. Initially, only the Breast Oncology Laboratory at the University of Michigan (where the CTC-ETI testing was developed) will be receiving and testing samples. Mayo Clinic will be trained to perform the CTC-ETI testing (using a written training program specific to CTC-ETI testing) once feasibility has been shown with the first 32 subjects evaluable for analytical validity. Once both study laboratory sites are established, a quality control program will be formalized and initiated. Blinded QC/proficiency samples and processing instructions will be provided to each operator at the study laboratory sites every two to three months, and the results will be sent back to the Sponsor for evaluation and documentation. Trainees with significant variations from expected results will be retrained, and all study samples evaluated by those individuals will be re-reviewed.

# 8.0 STUDY METHODS & PROCEDURES

## 8.1 INVESTIGATIONAL SITES

There will be approximately 25 geographically dispersed clinical sites enrolling subjects into this study. Each participating site will be assigned a unique site identification number (for example 01, 02, etc.) by the Sponsor. Prior to subject enrollment, each participating site is required to obtain its IEC/IRB Approval of the protocol, informed consent and any other subject materials and must provide documentation of approval to the Sponsor.

CTC-ETI samples will be sent to a specified study laboratory site for processing, and we anticipate having two to three study laboratory sites available for processing of the CTC-ETI samples. Initially, all samples will be sent to the University of Michigan Breast Oncology Laboratory for processing. The goal is to use only the University of Michigan Breast Oncology Laboratory until the first 32 subjects evaluable for analytical validity have had a baseline sample for CTC-ETI calculation (see Section 12.2). Subsequently, samples will be sent to the University of Michigan and Mayo Clinic (once they have been appropriately

trained and have shown proficiency as described in Section 7.0). Each site will be assigned to a study laboratory by the Sponsor and will be notified where to send samples.

### 8.2 Study Subject Selection and Registration

This will be a multi-center, prospective study. Subjects who qualify for the study based on the inclusion and exclusion criteria identified in Sections 6.1 and 6.2 above, and who sign an informed consent form, will be assigned a Subject ID number consisting of the site identification number (for example 01, 02, etc.) and an ascending sequential number assigned by the site (001, 002, 003, etc). For example, site 01's first subject will be 001, so the Subject ID number for that subject will be 01-001. Each site will maintain a screening/enrollment log (**Appendix B**) that includes all subjects screened and enrolled at the site. The log will contain the reason why a subject that was screened for the study was not enrolled. For subjects that are enrolled, the log will contain the Subject ID number assigned by the site, the date the informed consent document was signed, and any other required screening information. Any deviations from the eligibility criteria will need to be sent to the Sponsor via e-mail prior to enrollment of the subject into the study. The deviation will be reviewed by the Sponsor and the study PI, and the site will be notified via e-mail about whether or not the deviation is acceptable. The site will be required to complete a Protocol Deviation form (**Appendix B**) for all such deviations, whether approved or not, and keep a running log of all such deviations (**Appendix B**). Upon the enrollment of a subject (within 1 day of the consent form being signed), the site must e-mail a PDF copy of the completed Eligibility Form (**Appendix B**) to the Sponsor Study Manager.

### 8.3 Blinding & Use of CTC

**Study laboratory personnel will not receive any information about the clinical demographics, treatment, or outcomes of the subjects and the CTC-ETI results will not be reported back to the physicians and/or subjects (Exception: Site Principal Investigators will be notified by the Sponsor when a subject has an unsuccessful baseline CTC-ETI calculation, and these subjects will be notified by the site Principal Investigator and/or the appropriate site staff that they will be removed from further participation in the study). The CTC-ETI results will not be used for patient/disease management.**

#### 8.3.1 Blinding of CTC-ETI assay operators

All assays will be performed without knowledge of clinical demographics, treatment, outcomes or other information. The person receiving the samples in the laboratory and logging them in on the accessioning log sheet (**Appendix B**) will remove the tube labels, attach them to the accession log, and blind the samples with a sequential study and lab specific Sample ID (i.e. CP2L1-0001). The person receiving the samples will NOT be the technician who processes the samples. The technician(s) processing the samples will not have access to the sample accession log, site information, or any clinical demographics, treatment, or outcomes data, thereby blinding the technician that runs the samples to the source of the samples and all clinical data. On bi-weekly basis (or otherwise as needed/requested), the laboratory will fax a copy of the accession log to the

Sponsor. The laboratory must notify the Sponsor of all samples unable to be processed due to a pre-analytical error within 7 days after receipt of the sample (**Appendix E**) and of all samples where a CTC-ETI category is unable to be determined (**Appendix E**) within 14 days after the receipt of the sample by providing the Sponsor a copy of the CTC-ETI requisition and results forms (**Appendix B**).

#### 8.3.2 Blinding of clinicians

**Results regarding either CTC enumeration or CTC-ETI will not be provided to the subject or her caregiver.** Since CTC-phenotype is investigational and has not been shown to predict benefit from specific therapies such as ET or anti-HER2, if a subject is found to be either CTC-ER negative or CTC-HER2 positive, neither the subject nor their clinician will be notified. Physicians will be notified by the Sponsor when a subject has an unsuccessful baseline CTC-ETI calculation, and these subjects will be notified by the physician and/or site study staff that they will be removed from further participation in the study.

#### 8.3.3 Use of CTC as standard of care

A CTC count of 5 or more per 7.5mL of WB, as detected by the CELLSEARCH® CTC assay, is associated with decreased PFS and decreased OS in subjects with MBC. This *in-vitro* diagnostic assay was cleared by the FDA as an aid in the monitoring of women with MBC. Serial testing for CTC should be used in conjunction with other clinical methods for monitoring MBC. Evaluation of CTC at any time during the course of disease allows assessment of patient prognosis and is predictive of PFS and OS. Thus, if a physician or subject wishes to use CELLSEARCH® for clinical purposes, they may do so through commercial sources available in the U.S., by using a CLIA-approved commercial laboratory. However, only objective physical or radiographic findings, as described in Section 9 below, and not CTC levels, will be used to determine disease progression in this study.

### 8.4 STUDY SCHEMA

The imaging studies and physician assessments outlined in the study schema below are considered part of routine clinical care while the medical record review / history, the blood draws for CTC-ETI testing, and the optional provision of archival tissue specimens are being done specifically for the purposes of this research study.

| Eligibility: ER positive, Her2 negative, progressive MBC starting new Endocrine Therapy | BL [1] | 1 mo [2] | 2 mo [2] | 3 mo [2] | 6 mo [2] | 9 mo [2] | Off Study / 12 mo [2,3] |
|---|---|---|---|---|---|---|---|
| Medical Record Review / History | X | | | | | | |
| Physician (or Designee): Assessment of Clinical Status | X | X | X | X | X | X | X |
| Blood draw: CTC-ETI + optional cfDNA | X | X | X | X | | | X |
| Imaging Assessments: Refer to Section 8.6 | X | | | X | ← Physician Discretion | → | X |
| *Optional*: Provision of Archival Tissue Specimens (blocks or slides) | X | | | | | | |

*Abbreviations: BL=baseline ; mo = month*

[1] *Baseline Assessments: Must be done within 30 days prior to initiation of new endocrine therapy.*

[2] *Follow-up Assessments: Must be performed +/- 14 days of designated time point. Frequency of clinical follow-up is every 3 months. Imaging between 3-12 months is at the discretion of the managing physician, however, all target and/or non-target lesions selected at baseline must be assessed at 3 months and at progression/off study/12 months with the same imaging modality used at baseline (RECIST v1.1 guidelines).*

[3] *12 Month or Off Study Assessments: Must be done at the time the patient is taken off the study (i.e. at disease progression, discontinuation of therapy, or 12 months after the initiation of therapy without disease progression, whichever occurs first). All target and/or non-target lesions selected at baseline must be assessed with the same imaging modality used at baseline (RECIST v1.1 guidelines).*

At each CTC-ETI blood draw and/or follow-up evaluation, the physician's overall impression (or their designee, such as nurse practitioner or physician's assistant) of the subject's clinical status, as well as the results of any serum tumor marker testing done as part of routine care, will be recorded on the appropriate CRF (**Appendix B**).

### 8.5 ALLOWABLE TREATMENT(S)

- Treatment must be with an ET for subjects with ER positive, HER2 negative MBC. Choice of ET is left to the treating physician's discretion and include, but are not limited to, surgical ovariectomy, tamoxifen, LH-RH agonists, aromatase inhibitors, fulvestrant, megestrol acetate, or pharmacologic doses of estrogen.
- Combination ET is allowed if the treating physician deems it is appropriate.
- Combination of ET with everolimus is allowed if the treating physician deems it is appropriate.
- Subjects who are participating in a therapeutic clinical trial addressing ET plus or versus another, unapproved investigational therapy are not eligible.
- Co-administration of bisphosphonates, RANKL inhibitors or other supportive-care therapies that are considered standard of care are allowed.
- Palliative radiotherapy is allowed for treatment of pre-existing symptoms or conditions, however, palliative radiotherapy for emerging symptoms or conditions would be considered treatment for progressive disease.

### 8.6 IMAGING

All subjects will undergo an evaluation of metastatic sites by means of standard imaging studies at baseline that must be performed within 30 days prior to commencing a new line of ET. All imaging studies will be repeated 3 months after the initiation of therapy (within +/- 14 days) and when the patient is taken off study (i.e. maximum of 12 months after the initiation of therapy, at the time of disease progression, or at the discontinuation of treatment, whichever occurs first) **using the same imaging modalities employed at baseline to assess the selected target and/or non-target lesions (RECIST v1.1 guidelines)**. Any other imaging performed during the course of the study will be at the discretion of the managing physician.

NOTE: Clinicians may use their discretion regarding which imaging studies they prefer, but at baseline and each follow-up imaging assessment, subjects must have the same type of imaging performed for evaluation of the selected target and non-target lesions:

- The protocol allows for PET/CT alone or CT scans of the chest/abdomen/pelvis alone. If the CT scans provide enough of an evaluation of bone metastases, a standard bone scan will be optional.

Subjects are off study at time of progression or after 12 months of ET (whichever occurs first). Disease status will be assessed according to RECIST v1.1 criteria [29], described in detail in Section 9 below.

### 8.7 BLOOD SAMPLE COLLECTION & HANDLING PROCEDURES

Prior to collection of blood, the phlebotomist, nurse or investigator must confirm that a valid signed informed consent document has been obtained from the subject and the subject has not been exposed to a Category A infectious substance. All subjects will have blood drawn for CTC-ETI calculation and for optional cfDNA analysis at baseline (within 30 days prior to the initiation of ET) and then subsequently 1, 2, 3 and up 12 months after the initiation of therapy, at the time of disease progression, or at the discontinuation of treatment, whichever occurs first (within +/- 14 days of the specified time point). Only subjects with a successful baseline CTC-ETI calculation will have the subsequent serial blood draws performed. The study Principal Investigator will be notified by the Sponsor about the status of the baseline CTC-ETI calculation (i.e. successful vs. unsuccessful) within 15 days of receipt of the sample by the study laboratory. Subjects with an unsuccessful baseline CTC-ETI calculation will be will be excluded from further blood draws and will not be followed for progression or survival. These subjects will be notified by the study Principal Investigator and/or the appropriate site staff that they are being removed from further participation in the study.

Samples should be drawn just prior to the next administration of therapy. This protocol is designed to allow for blood donation in concert with the timing of therapy. We do not anticipate the need to schedule subjects for blood donation on a different schedule than their therapy or standard of care follow-up dictates. Refer to **Appendix C** for the specific Blood Sample Collection & Handling Procedures.

**ONLY COLLECT AND SHIP SAMPLES FOR CTC-ETI TO THE DESIGNATED STUDY LABORATORY ON MONDAY THROUGH THURSDAY. DO NOT DRAW SAMPLES ON A FRIDAY OR THE DAY BEFORE A HOLIDAY.**

### 8.8 UNANTICIPATED ADVERSE DEVICE EFFECTS

This is a minimal risk, non-intervention, non-treatment protocol. Unanticipated Adverse Device Effects are unlikely in this study; however, adverse events related to the blood collection such as syncope or uncontrolled bleeding could occur and must be reported (**Appendix B**). Timely, accurate, and complete reporting of this safety information is crucial for the protection of subjects. An investigator shall submit to the study Sponsor and to the reviewing IRB a report of any unanticipated adverse device effects occurring during an investigation as soon as possible, but no later than 10 working days after the investigator first learns of the effect. All adverse events across centers will be reviewed by the Sponsor and study Principal Investigator. See the Contact Information Sheet for the appropriate Sponsor contacts.

Janssen Diagnostics, LLC
700 Route 202 South
Raritan, NJ 08869

### 8.9 Blood Sample Processing Procedures for CTC-ETI

Samples will be shipped to the designated laboratory for processing. Two laboratories will be utilized: University of Michigan and Mayo Clinic. Upon receipt in the laboratory, the samples for the CTC-ETI calculation will be accessioned and blinded (as described in Section 8.3.1 above), the blood will be pooled, and four separate 7.5mL aliquots will be created and evaluated for CTC + ER, CTC + Bcl-2, CTC + HER2, and CTC + Ki67 using the CELLSEARCH® CXC assay and marker reagents. Any excess blood will be aliquoted into a separate 7.5mL aliquot and saved for up to 96 hours (4 days) after the time of collection to be used as a backup in case one or more of the assays has a technical failure and needs to be re-run. Remaining blood will be appropriately disposed of upon successful completion of the CTC assays. Results of the CTC-ETI assay will be recorded on the appropriate CRF(s) (**Appendix B**) by the study laboratory. **As indicated above in Section 8.3.1, the study laboratory will notify the Sponsor via e-mail of all samples unable to be processed due to a pre-analytical error within 7 days after receipt of the sample and of all samples with an unsuccessful CTC-ETI calculation within 14 days after the receipt of the sample and provide a PDF copy of the CTC requisition and results forms.** In the case a patient has four cartridges containing ≥5 CTC/7.5 ml, the study laboratory will also fix and store 2 cartridges (HER2 and BCL2) containing 5 or more CTC at -20°C for future molecular analyses (i.e. FISH). The other 2 cartridges (ER and Ki67) containing 5 or more CTC will be stored in the refrigerator (4°C) for future CTC-DNA extraction. In the case cartridges contain 1-4 CTC, they will be all stored in the refrigerator (4°C) for future CTC-DNA extraction.

#### 8.9.1 Pooling

In the study laboratory, the blood from the five different CellSave Preservative Tubes collected from each subject will be pooled, mixed together (total volume of ~40-50mL), and separated into five different 7.5mL aliquots (if there is a sufficient volume of blood). A minimum of four 7.5mL aliquots are required for CTC-ETI testing. Four out of the five aliquots will be used to enumerate CTC and to determine ER, Bcl-2, HER2, or Ki67 expression using the CELLSEARCH® CXC assay and to calculate the final CTC Assigned Points and CTC Biologic Points. The fifth aliquot (if a sufficient volume of blood was available) will be used as backup in case any instrument and/or reagent failures cause a loss of results from one of the first four aliquots. If there are no assay failures, the remaining volume of blood will be processed and stored for future molecular analysis of cfDNA. The purpose is to test the feasibility of extract cfDNA from CellSave tube and compared to the cfDNA extracted from the Streck tubes.

#### 8.9.2 CTC/Marker Enumeration

CTC will be isolated from each of the four different 7.5mL aliquots of blood utilizing the CELLSEARCH® System (Veridex, LLC) and the CELLSEARCH® CXC Kit according to the manufacturer's instructions and characterized using the following PE labeled antibodies: ER, Bcl-2, HER2, and Ki67. Before scanning the samples or controls on the CELLTRACKS ANALYZER II®, scan the BMC first and then scan the blood samples and positive/negative controls, loading the appropriate test and marker definitions for each one prior to scanning.

The relative expression of each biomarker will be visually determined as 0, 1+, 2+, or 3+ based on the expression of that marker in cultured human cell lines known to express that marker very highly, not at all, or in an intermediate fashion. Two separate readers at each laboratory will review the images from each sample to determine the CTC count and the number of CTC that are marker positive in each aliquot, and discordant results (CTC vs. not and marker positive vs. negative) will be reconciled by a joint review. CTC-ETI results (CTC counts, proportion of CTC positive for each marker, CTC-ETI Assigned Points, CTC-ETI Bio-Points, final CTC-ETI Score) and relative Category (low, intermediate, high) will be assigned based on the joint results.

- CTC enumeration: CTC are defined as DAPI+, CK+, and CD45- events that are larger than 4 microns. The number of CTC per 7.5mL of WB in each of the four 7.5mL aliquots will be determined according to the manufacturer's instructions. The average # of CTC per 7.5mL of WB for all aliquots will be calculated and used to determine the CTC-Assigned Points in the CTC-ETI. Refer to **Table 1** in Section 5.2 and **Appendix E** for specific instructions on determination of CTC-Assigned Points.

- CTC characterization (Identification of ER, Bcl-2, HER2, Ki67): The CTC will be visually scored for each marker for different degrees of staining above background in the ER-PE/Control channel, Bcl-2-PE/Control channel, HER2-PE/Control channel, and Ki67-PE/Control channel, respectively. The visual score for each CTC will be recorded along with the event number.

– ER and Ki67 staining (both nuclear markers) will be scored by 4 different degrees of staining: 0 (negative or cytoplasmic only staining), 1+ (slight nuclear positivity), 2+ (moderate nuclear positivity), 3+ (strong nuclear positivity). For the ER staining, a distinction between negative and cytoplasmic will be made in the laboratory records.

– HER2 and Bcl-2 staining (both cytoplasmic markers) will be scored by 4 different degrees of staining: 0 (negative or nuclear staining), 1+ (slight cytoplasmic positivity), 2+ (moderate cytoplasmic positivity), 3+ (strong cytoplasmic positivity).

– CTC with marker scores of 2+ or 3+ will be considered positive for the marker. CTC with marker scores of 0 or 1+ will be considered negative for the marker.

– The CTC-Biologic Points of the markers will be calculated based on the % of CTC that are positive for the respective marker (see **Table 2** in Section 5.2).

– CTC-ETI Scores and relative Category (low, intermediate, high) will be calculated as described in **Tables 3** and **4** in Section 5.2, respectively.

– The BioMarQ data will be collected and evaluated separately (see Section 12.7).

## 8.10 TISSUE SAMPLE COLLECTION & HANDLING PROCEDURES

For each subject, archived tumor biopsy and/or surgical resection specimens will be obtained, if available, from the primary and metastatic tumor sites. Submission of these specimens is strongly urged but not mandatory. They will be collected at each site, clearly labeled with the protocol number, subject study number, type of sample and date of collection. See **Appendix D** for the specific Tissue Sample Collection & Handling Procedures.

Janssen Diagnostics, LLC
700 Route 202 South
Raritan, NJ 08869

Preferably, one block from the primary tumor and one block from the metastatic sites, if available, will be submitted. If both are not available, whichever is available will be requested. Recognizing not all pathology laboratories will provide tissue blocks, we will request tissue types using the following preferred hierarchy:

1. Tissue block; or

2. Ten (10) unstained glass slides from each tissue block consisting of 5μm thick tissue sections on "plus" (i.e. positively charged) slides.

Tissue blocks are preferable, since it will allow for construction of a tissue microarray. In addition, if tissue blocks are provided, RNA will be harvested from 7μm tissue sections for intrinsic subtype [32] and 21 gene recurrence score assays [33-34].

The referring pathologist should examine a H&E section of the block or slides to be sent, to assess for the presence of viable tumor. Tissue will be analyzed using standard IHC for ER, Bcl-2, HER2 and Ki67. HER2 will also be analyzed with FISH. Remaining tissue blocks and/or slides will be archived and consolidated in one place at the University of Michigan for storage and future analyses that will be determined at a later date. A separate request to perform other investigational assay using these stored pathologic samples will be submitted for IRB approval.

## 8.11 TISSUE SAMPLE PROCESSING PROCEDURES

Sponsor and/or its designee will document and archive all tissue samples (either blocks or slides) that are received for each subject. Upon completion of the enrollment into the study, Dr. Dafydd Thomas from the University of Michigan Department of Pathology will perform the appropriate pathology, IHC, and FISH assessments on the archived tissue specimens.

Immunohistochemical staining will be performed on the DAKO Autostainer (DAKO, Carpinteria, CA) using DAKO LSAB+ and diaminobenzadine (DAB) as the chromogen. De-paraffinized sections of formalin fixed five-micron thick tissue sections will be labeled with anti-Bcl-2 (mouse monoclonal antibody, 1:100, DAKO, M-0887, clone 124), anti-HER2 (Rabbit polyclonal antibody, 1:2000, DAKO, A0485), anti-ERα (mouse monoclonal antibody, 1:50, DAKO, M-7047, clone 1D5) or anti-Ki67 (mouse monoclonal antibody, 1:100, DAKO, M-7240, clone MIB-1). Microwave citric acid epitope retrieval will be used for antibodies against Bcl-2 and HER2. Epitope retrieval in 1mM EDTA pH 8 will be used for anti-Ki67 and ERα. Appropriate negative (no primary antibody) and positive controls (breast carcinoma) will be stained in parallel with each set of tumors studied. IHC scoring will be performed using the method of Harvey et al [35] by two independent reviewers. Discrepancies will be resolved via repeat review of the slides in question by the reviewers to reach consensus.

HER2 fluorescence in-situ hybridization (FISH) will be performed as previously described [36], with the exception that the HER2/Chr17 probe pair will be utilized (KI-10701, Veridex, Raritan, NJ). Briefly, 5-micron thick tissue sections are hybridized to the pre-labeled probes (HER2, red and Chr17 Satellite Enumeration probes, green) after dewaxing, rehydration and digestion of the slide. The slides are washed under defined conditions and the nuclei are

Janssen Diagnostics, LLC
700 Route 202 South
Raritan, NJ 08869

labeled with DAPI in a non-fade mounting media. After overnight drying the slides are read at 1000x for the three different fluorochromes and at least 100 cells are enumerated for the presence of Chr17 and HER2. Cells are considered diploid if the ratio between HER2 and Chr17 is below 1.8, equivocal between 1.8 and 2.2 and amplified if the ratio is above 2.2 as per CAP guidelines [30].

### 8.12 MOLECULAR ANALYSIS OF CELL-FREE DNA

The optional WB sample collected in the Streck tube will be processed within 14 days upon receipt at the laboratory. The plasma will be stored either at -20 or -80 °C and stored for future analysis and clinical correlation after the end of the study.

## 9.0 IMAGING MEASUREMENT AND INTERPRETATION CRITERIA

Subjects will undergo serial, standard of care imaging to follow disease outcomes, with disease assessment according to RECIST v1.1 criteria [29]. All baseline evaluations should be performed as close as possible to the treatment start and never more than 30 days prior to the beginning of treatment.

### 9.1 MEASURABILITY OF LESIONS

Measurable Tumor Lesions: Lesions that can be accurately measured in at least one dimension (longest diameter in the plane of measurement is to be recorded) with a minimum size of:

- 10mm by CT scan (irrespective of scanner type, CT scan slice thickness no greater than 5mm) and MRI (no less than double the slice thickness and a minimum of 10mm)
- 10mm caliper measurement by clinical exam (when superficial)
- 20mm by chest X-ray (if clearly defined and surrounded by aerated lung)
- **Malignant Lymph Nodes:** To be considered pathologically enlarged and measurable, a lymph node must be >15mm in short axis when assessed by CT scan (CT scan slice thickness no greater than 5mm). At baseline and in follow-up, only the short axis will be measured and followed.
- **Lytic bone lesions or mixed lytic-blastic lesions** with identifiable soft tissue components that can be evaluated by cross-sectional imaging technique such as CT or MRI can be considered measurable if the soft tissue component meets the definition of measurability described above.
- **"Cystic lesions"** thought to represent cystic metastases can be considered measurable if they meet the definition of measurability described above. However, if non-cystic lesions are present in the same patient, these are preferred for selection as target.

Non-Measurable Tumor Lesions: Non measurable lesions are all other lesions, including lesions too small to be considered measurable (longest diameter <10mm or pathological

lymph nodes with 10 to <15mm short axis), as well as truly non-measurable lesions. Lesions considered truly non-measurable include: leptomeningeal disease, ascites, pleural or pericardial effusions, inflammatory breast cancer, and lymphangitis involvement of skin or lung, abdominal masses/abdominal organomegaly identified by physical exam that is not measurable by reproducible imaging techniques.

– **Blastic bone lesions** are non measurable.

– **Lesions with prior local treatment**, such as those situated in a previously irradiated area or in an area subjected to other loco-regional therapy, are usually not considered measurable unless there has been demonstrated progression in the lesion. Study protocols should detail the conditions under which such lesions would be considered measurable.

### 9.2 SELECTION OF TARGET AND NON-TARGET LESIONS

At baseline, measurable lesions up to a maximum of five lesions total (and a maximum of two lesions per organ) representative of all involved organs should be identified as **target lesions**. Target lesions should be selected on the basis of their size (lesions with longest diameter) and be representative of all involved organs, as well as their suitability for accurate repetitive measurements. All measurements should be recorded in metric notation using calipers if clinically assessed. A sum of the diameters (longest for non-nodal lesions, short axis for nodal lesions) for all target lesions will be calculated and reported as the baseline sum diameters. The baseline sum diameters will be used as reference to further characterize any objective tumor regression in the measurable dimension of the disease. If lymph nodes are to be included in the sum, only the short axis will contribute.

All other lesions (or sites of disease) not identified as target lesions, including pathological lymph nodes and all non-measurable lesions, should be identified as **non-target lesions** and be recorded at baseline. Measurements of these lesions are not required and they should be followed as “present” or “absent” or in rare case, “unequivocal progression”.

### 9.3 DETERMINATION OF OBJECTIVE TUMOR RESPONSE

The subject’s objective tumor response will be determined from imaging time point to imaging time point using the RECIST v1.1 criteria [29]. Objective status is to be recorded at each evaluation.

Objective Response Criteria:

- Evaluation of Target Lesions
  - *Complete Response (CR)*: Disapperance of all target lesions. Any pathological lymph nodes (whether target or non-target) must have reduction in short axis to <10mm.
  - *Partial Response (PR)*: At least a 30% decrease in the sum of diameters of target lesions, taking as reference the baseline sum of diameters.

Janssen Diagnostics, LLC
700 Route 202 South
Raritan, NJ 08869

– *Stable Disease (SD)*: Neither sufficient shrinkage to qualify for PR nor sufficient increase to qualify for PD, taking as reference the smallest sum diameters while on study.

– *Progressive Disease (PD)*: At least a 20% increase in the sum of diameters of target lesions, taking as reference the smallest sum on study (this may includes the baseline sum if that is the smallest on study). The sum must also demonstrate an absolute increase of at least 5mm. The appearance of one or more new lesions is also considered progression. However, new lesions on bone scintigraphy must be confirmed to be litic, or mixed lytic/sclerotic, to distinguish from a "healing flare" bone scan response.

**Special notes on the assessment of target lesions**

- **Lymph nodes identified as target lesions** should always have the actual short axis mesurament recorded even if the nodes regress to below 10mm on study. When the lymph nodes are included as target lesions, the "sum" of lesions may not be zero even if complete response criteria are met since a normal lymph node id defined as having a short axis of <10mm.

- **Target lesions that become "too small to measure"**. While on study, all lesions (nodal and non-nodal) recorded at baseline should have their actual mesurements recorded at each subsequent evaluatuion, even when very small. However, sometimes lesions or lymph nodes become so fain on a CT scan that the radiologist may not feel confortable asssigning an exact mesure and may report them as being "too small to mesure", in which case a default value of 5mm should be assigned.

- **Lesions that split or coalesce on treatment.** When non-nodal lesions "fragment", the longest diameters of the fragmented portions should be added together to calculate the target lesion sum. Similarly, as lesions coalesce, a plane between them may be maintained that would aid in obtaining maximal diameter mesuraments of each individual lesion. If the lesions have truly coalesced such that they are no longer seperable, the vector of the longest diameter in this instance should be maximal longest diameter for the "coalesced lesion".

PRODUCTS ARE FOR RESEARCH USE ONLY AND THEIR PERFORMANCE CHARACTERISTICS ON CLINICAL SAMPLES HAVE NOT BEEN DETERMINED

Janssen Diagnostics, LLC
700 Route 202 South
Raritan, NJ 08869

- Evaluation of Non-Target Lesions
  - *Complete Response (CR)*: Disapperance of all non-target lesions and normalization of tumor marker level. All lymph nodes must be non-pathological in size (short axis <10mm).
  - *Non-CR/Non-PD*: Persistence of one or more non-target lesion(s) and/or maintenance of tumor marker level above the normal limits.
  - *Progressive Disease (PD)*: Unequivocal progression of existing non-target lesions (unequivocal means, comparable in magnitude to the increase that would be required to declare PD for measurable disease or an overall substantial increase in tumor burden that merits treatment discontinuation). The appearance of one or more new lesions is also considered progression.
  - *Not Evaluated (NE)*: No non-target lesions were assessed, because no imaging/mesurament is done at all a particular time point. If only a subset of lesions are made at an assessment, usually the case is also considered NE at that time point, unless a convincing argument can be made that the contribution of the individual missing lesion(s) would not change tha assigned time to response. This would most likely happen in the case of PD.

**Table 5** below provides a summary of the overall response status calculation at each time point for patients who have measurable disease at baseline. When subjects have non-measurable (therefore non-target) disease only, **Table 6** is to be used for the determination of overall response status calculation at each time point.

**Table 5. Time point response: Patients with target (+/- non-target) disease.**

| Target Lesions | Non-target Lesions | New Lesions | Overall Response |
|---|---|---|---|
| CR | CR | No | CR |
| CR | Non-CR/non-PD | No | PR |
| CR | NE | No | PR |
| PR | Non-PD/or not all evaluated | No | PR |
| SD | Non-PD/or not all evaluated | No | SD |
| Not all evaluated | Non-PD | No | NE |
| PD | Any | Yes or No | PD |
| Any | PD | Yes or No | PD |
| Any | Any | Yes | PD |

Abbreviations: CR= Complete Response, PR= Partial Response, SD= Stable Disease, PD= Progressive Disease, NE= Inevaluable

Janssen Diagnostics, LLC
700 Route 202 South
Raritan, NJ 08869

**Table 6. Time point response: Patients with non-target disease.**

| Non-Target Lesions | New Lesions | Overall Response |
|---|---|---|
| CR | No | CR |
| Non-CR/non-PD | No | Non-CR/non-PD[1] |
| Not all evaluated | No | NE |
| Unequivocal PD | Yes or No | PD |
| Any | Yes | PD |

Abbreviations: CR= Complete Response, PD= Progressive disease, NE= Inevaluable

[1]Non-CR/non-PD is preferred over "Stable disease" for non-target disease since SD is increasingly used as an endpoint for assessment of efficacy in some trials. To assign this category when no lesions can be measured is not advised.

### 9.4 Definition of Rapid Disease Progression

Rapid disease progression will be defined as disease progression according to RECIST v1.1 criteria or death due to MBC within 3 months of starting a new ET. Symptomatic deterioration or rising serum tumor markers (i.e. CA15-3/27.29 or CEA) or rising CTC counts (ordered by physician as routine care, since the CTC level done as part of this trial will not be returned to the treating physician) may be an indication to repeat imaging earlier than planned at the treating physician's discretion, but will NOT be considered evidence of rapid disease progression. A rising serum tumor marker in the first 3 months of follow-up may represent a tumor marker spike and is NOT an indication to perform repeat imaging sooner than stipulated in the protocol unless the subject has other signs or symptoms of progression.

### 9.5 Definition of Progression Free Survival (PFS)

PFS will be measured as the time from the date of starting ET until the date of first documentation of progressive disease according to RECIST v1.1 criteria, or death due to any cause. In the absence of these events, PFS will be censored at the date of the last objective disease assessment (up to a maximum of 12 months after the initiation of ET).

## 10.0 SUBJECT COMPLETION/WITHDRAWAL

### 10.1 Completion

A subject will be considered to have completed the study if she has completed all blood draws and imaging assessments for up to 12 months, until her disease progresses, or death due to any cause, whichever occurs first.

### 10.2 Discontinuation of Treatment

If a subject discontinues study treatment before the end of the 12 month period or prior to disease progression, obtain end-of-treatment assessments (see Study Schema in Section 8.4) and document on the appropriate CRFs (**Appendix B**).

Janssen Diagnostics, LLC
700 Route 202 South
Raritan, NJ 08869

### 10.3 Withdrawal From the Study

A subject will be withdrawn from the study for any of the following reasons:

- Protocol deviation(s) not approved by the Sponsor.
- Only subjects with a successful baseline CTC-ETI calculation will have the subsequent serial blood draws performed. Subjects with an unsuccessful baseline CTC-ETI calculation will be removed from the study, excluded from further blood draws, and will not be followed for progression or survival.
- Subjects discontinuing study treatment within 3 months after the initiation of the new treatment for any reason other than disease progression will be excluded from further blood draws and will not be followed for progression or survival.
- Subject withdraws consent. A subject who withdraws their consent and no longer wishes to participate in the study will have the following options regarding the future research on their stored materials (i.e. cartridges containing CTC and tissue samples):
    - The stored CTC cartridges and tissue samples will be retained and used in accordance with the subject's original informed consent.
    - The subject may withdraw consent for the additional research, in which case the CTC cartridges will be destroyed and no further testing will take place and the tissue samples will be returned to the providing instutition. To initiate the sample destruction and return process, the investigator must notify the Sponsor in writing to request sample destruction. The Sponsor will, in turn, contact the study laboratory to execute destruction of the CTC cartridges and the University of Michigan to have the tissue samples returned. If requested, the investigator will receive written confirmation from the Sponsor that the samples have been destroyed.
- Lost to Follow-Up. If a subject is lost to follow-up, every reasonable effort must be made by the study-site personnel to contact the subject and determine the reason for discontinuation/withdrawal. The measures taken to follow up must be documented.

When a subject withdraws their consent or is withdrawn from the study for the reasons mentioned above before completing the study, the reason(s) for withdrawal must be documented in the appropriate CRF(s), the site enrollment log, and in the source documentation.

Janssen Diagnostics, LLC
700 Route 202 South
Raritan, NJ 08869

# 11.0 DATA COLLECTION AND HANDLING

## 11.1 CASE REPORT FORMS

Clinical and laboratory data will be collected from the subject's medical records / clinic chart using paper CRFs [**Appendix B**]. Original pages for all completed CRFs will be sent to the Sponsor on a monthly basis for input into a database to be used for the analyses. Sites will be notified about the status of the baseline sample (successful or unsuccessful CTC-ETI calculation) by the Sponsor within 15 days after receipt of the baseline sample by the study laboratory. The table below lists the CRFs and provides details about their completion and any special instructions for submission to the Sponsor:

| CRF Name | Instructions |
|---|---|
| Eligibility Form (E01) | Required form for all subjects who sign a consent form. Complete at the time of enrollment. E-mail a PDF copy of this completed form to the Sponsor within 1 day of the consent form being signed. Any deviations from the eligibility criteria must be reviewed and approved by the Sponsor prior to enrollment of the subject and will require a completed Protocol Deviation form. |
| Baseline Form (BL01) | Required form for all subjects who have a successful baseline CTC-ETI calculation. Complete within 30 days after enrollment of a subject. |
| Prior Breast Cancer Therapy Form (PT01) | Required form for all subjects who have a successful baseline CTC-ETI calculation. Complete within 30 days after enrollment of a subject. Include all prior and ongoing treatments for breast cancer. Use additional pages if necessary, indicating A, B, C, etc. after page number to indicate multiple pages. |
| On-Study Therapy Form (TX01) | Required form for all subjects who have a successful baseline CTC-ETI calculation. Update on an ongoing basis and complete within 30 days of a subject going off study. |
| Physicians Notes / Serum Tumor Markers (PNST01) | Required form for all subjects who have a successful baseline CTC-ETI calculation. Update at each visit (i.e. baseline, months 1, 2, 3, 6, 9 and 12/off study). Complete within 30 days of a subject going off study. |
| Baseline Tumor Assessment Form (TM01) | Required form for all subjects who have a successful baseline CTC-ETI calculation. Complete within 30 days after the initiation of the new line of ET. Imaging methods used to assess target and/or non-target lesions at baseline should be the same throughout the course of the study. |
| Follow-Up Tumor Assessment Forms (FT01, Pages 1 & 2) | Required form for all subjects who have a successful baseline CTC-ETI calculation. Complete within 30 days of each follow-up disease assessment visit (i.e. Month 3, 6, 9, and 12/off study). Record target and non-target lesions on Page 1 in same order as recorded on the baseline form (TM01), even if not assessed. Record the evaluation of the target and non-target lesions compared to the prior imaging study (Page 1). Record any new lesions on Page 2 along with overall assessment compared to prior imaging study and any comments. |
| CTC-ETI Blood Sample Requisition Form (LAB01) | Required form for all subjects at each blood draw. Section A to be completed by the site at the time of the blood draw, and the original copy of the form is to be included with the blood shipment to the study laboratory. Study laboratory will complete Section B of the form, make a copy of the completed form for their records, and send the completed original to the Sponsor. |

| | |
|---|---|
| Cell-Free DNA Blood Sample Requisition Form (LAB03) | Required form for all subjects at each blood draw if consent was provided for this optional blood draw. Section A to be completed by the site at the time of the blood draw, and the original copy of the form is to be included with the blood shipment to the study laboratory. Study laboratory will complete Section B of the form, make a copy of the completed form for their records, and send the completed original to the Sponsor. |
| CTC-ETI Results Form (LAB02) | Required form for all blood samples received by a study laboratory. If a sample cannot be processed due to a pre-analytical error, the study laboratory must check the appropriate boxes at the top of the form and e-mail a PDF copy (along with the appropriate requisition form – LAB01) to the Sponsor within 7 days after receipt of the sample. If a sample can be processed, but one or more of the aliquots fail, resulting in the inability to determine a CTC-ETI category, a PDF copy of the completed form (along with the appropriate requisition form – LAB01) must be e-mailed to the Sponsor within 14 days after receipt of the sample in the laboratory. For all successful samples, the form should be completed within 30 days of the receipt of the sample in the laboratory. |
| Tissue Sample Requisition Form (TIS01) | Required form for all subjects who have a successful baseline CTC-ETI calculation. If no tissue is being provided, form must be completed within 30 days after enrollment of the subject and original sent directly to Sponsor. If tissue is being provided, site should complete Section A and include the original copy with the shipment of the tissue slides/blocks to the University of Michigan Tissue Bank. The University of Michigan will complete Section B of the form, make a copy of the completed form for their records, and send the completed original to the Sponsor. |
| Off Study Evaluation Form (OFF01) | Required form for all subjects who sign a consent form. Complete, including signature by the appropriate individual at the site with signatory authority, within 10 days of a subject going off study. |
| Protocol Deviation Form (DEV01) | Use this form to document any deviations from the study protocol. |
| Unanticipated Adverse Device Effects (AE01) | Required form for all subjects who sign a consent form. Any serious, unexpected adverse events related to the blood collection must be reported (see Section 8.8). Report any unanticipated adverse events to the Sponsor and the IRB as soon as possible, but no later than 10 working days after investigator first learns of the event. Complete within 30 days of a subject going off study. |

## 11.2 CASE REPORT FORM COMPLETION

Case report forms will be provided for each subject in printed format.

All printed forms must be filled out legibly in black ballpoint pen or typed. The appropriate page(s) of the CRF must be signed and dated by the investigator.

Worksheets may be used for the capture of some data to facilitate completion of the CRF. Any such worksheets will become part of the subject's source documentation. All data relating to the study must be recorded in CRFs prepared by the Sponsor. Data must be entered into CRFs in English. Designated study-site personnel must complete the CRF as soon as possible after a subject visit, and the forms should be available for review at the next scheduled monitoring visit.

The site Principal Investigator must confirm that all data entries in the CRFs are accurate and correct. All CRF entries, corrections, and alterations must be made by the Principal Investigator or other authorized study-site personnel. Corrections to paper CRFs must be made in such a way that the original entry is not obscured. Correction fluid or tape must NOT be used. The correct data must be inserted, dated, and initialed by the Principal Investigator or an authorized member of the study-site personnel. If multi-part pressure-sensitive CRFs are used, the study-site personnel must not write on separated parts of the CRFs left at the study site once the original has been sent to the sponsor. Completed CRFs will be continuously submitted according to the Sponsor's instructions and reviewed by the Sponsor to determine their acceptability. If necessary, Data Correction/Clarification Forms (DCFs) will be generated and transmitted to the study site. The investigator or an authorized member of the study-site personnel must complete, sign, and date the DCFs.

### 11.3 Data Quality Assurance / Quality Control

Steps to be taken to ensure the accuracy and reliability of data include the selection of qualified investigators and appropriate study sites, review of protocol procedures with the investigator and associated personnel before the study, and potential periodic monitoring visits by the Sponsor. Written instructions will be provided for collection, preparation, and shipment of blood and tissue samples (**Appendices C & D**).

Guidelines for CRF completion will be provided and reviewed with study personnel before the start of the study. The Sponsor will review CRFs for accuracy and completeness during on-site monitoring visits and/or after their return to the Sponsor; any discrepancies will be resolved with the investigator or designee, as appropriate. The data will be entered into the study database and verified for accuracy and consistency with the data sources.

### 11.4 Privacy of Personal Data

The collection and processing of personal data from subjects enrolled in this study will be limited to those data that are necessary to fulfill the objectives of the study.

These data must be collected and processed with adequate precautions to ensure confidentiality and compliance with applicable data privacy protection laws and regulations. Appropriate technical and organizational measures to protect the personal data against unauthorized disclosures or access, accidental or unlawful destruction, or accidental loss or alteration must be put in place. Sponsor personnel whose responsibilities require access to personal data agree to keep the identity of study subjects confidential.

The informed consent obtained from the subject includes explicit consent for the processing of personal data and for the investigator to allow direct access to his or her original medical records for study-related monitoring, audit, IEC/IRB review, and regulatory inspection. This consent also addresses the transfer of the data to other entities and to other countries.

The subject has the right to request, through the investigator, access to her personal data and the right to request rectification of any data that are not correct or complete. Reasonable steps will be taken to respond to such a request, taking into consideration the nature of the request, the conditions of the study, and the applicable laws and regulations.

Janssen Diagnostics, LLC
700 Route 202 South
Raritan, NJ 08869

Exploratory research is not conducted under standards appropriate for the return of data to subjects. In addition, the Sponsor cannot make decisions as to the significance of any findings resulting from exploratory research. Therefore, exploratory research data will not be returned to subjects or investigators, unless required by law. Privacy and confidentiality of data generated in the future on stored samples will be protected by the same standards applicable to all other clinical data.

All subject information remains confidential and will be maintained in the following manner.

a) Access to study data will only be permitted to necessary personnel.

b) All investigational site subject identifiers will already have been removed by the site before submission of the data to the Sponsor. Only the study-specific identifiers assigned by the site and will be maintained and used by the necessary Sponsor personnel.

c) Data will be stored in locked cabinets and/or on secure servers.

The Sponsor, the Principal Investigator, and all other individuals involved in the research study shall comply with all applicable Health Insurance Portability and Accountability Act (HIPAA) Privacy Requirements. The "HIPAA Privacy Requirements" refer collectively to the applicable provisions of the Administrative Simplification section of HIPAA- the Health Insurance Portability and Accountability Act of 1996, (as codified at 42 U.S.C. § 1320d - d-8) and any regulations promulgated there under, including without limitation, the federal privacy regulations (45 CFR Part 164) and the federal security standards (45 CFR Part 142). "PHI" refers to "protected health information" as defined in 45 CFR 164.504, and De-Identify shall be as defined in 45 CFR 164.514.

# 12.0 STATISTICAL CONSIDERATIONS

## 12.1 SAMPLE SIZE

A sample size of approximately 200 subjects will provide a sufficient number of evaluable subjects and statistical power for evaluation of the primary and secondary objectives, as described in subsequent sections.

It is estimated that a total of 120 subjects evaluable for clinical validity analyses will be required to obtain the minimum of 51 subjects with rapid progression for evaluation of the clinical validity of the CTC-ETI. It is assumed that up to 40% of enrolled subjects will not be evaluable for clinical validity (see Section 12.3). Enrollment will continue until a sufficient number of evaluable subjects, to achieve statistical power for the evaluation of the primary objectives, have been acquired.

## 12.2 1ST PRIMARY OBJECTIVE

For the first primary objective, which is to demonstrate that the CTC-ETI can be accurately determined at baseline in subjects with ER positive, HER2 negative MBC from multiple

Janssen Diagnostics, LLC
700 Route 202 South
Raritan, NJ 08869

centers across North America and Canada (analytical validity), the baseline blood sample will be evaluated for the following:

- successful calculation of CTC-ETI;
- successful enumeration of CTC in the four aliquots;
- successful determination of CTC Bio-Score for all 4 markers when the average number of cells is ≥5 CTC/7.5mL of blood

All subjects for whom the baseline blood sample is collected and shipped to the study laboratory without handling/pre-analytical errors will be considered evaluable for the purposes of this evaluation (i.e., evaluable for analytical validity analyses); reasons for non-evaluability will be described. For the evaluable subjects, the number (%) with a successful baseline CTC-ETI calculation, with exact binomial 95% confidence intervals (CI), will be determined. We will further summarize the success rates of the components of CTC enumeration and CTC Bio-Score assessment.

There are two independent, sequential analyses of analytical validity planned, the first after approximately 35-40 subjects have been enrolled and the second after approximately 70-80 subjects have been enrolled. Both analyses will be based on the binary endpoint of successfully calculating a CTC-ETI in the baseline sample. For each analysis, we desire ≥80% success, whereas ≤60% success is considered too low. If the number of successes is ≥24/32 then the null hypothesis is rejected with $\alpha$=0.057 (target $\alpha$=0.06); if the number of successes is ≤23/32 then the alternative hypothesis is rejected with $\beta$=0.175 (target $\beta$=0.20).

- Samples will initially all be sent to the University of Michigan Breast Oncology Laboratory, and we will evaluate analytical validity associated with assessing baseline CTC-ETI in blood collected at multiple clinical sites and processed in one study laboratory. Once the baseline CTC-ETI is (or is not) successfully calculated in the first 32 evaluable subjects (approximately the first 35-40 enrolled subjects) in the single study laboratory, the first analysis will take place.
- Subsequently samples will be sent to multiple study laboratories, and we will evaluate analytical validity associated with assessing baseline CTC-ETI collected at multiple clinical sites and assayed in multiple study laboratories. Once the baseline CTC-ETI is (or is not) successfully calculated in the next 32 evaluable subjects (approximately the next 35 enrolled subjects, or a total of approximately 70-80 enrolled subjects) at multiple study laboratories, the second analysis will take place in the subsequent 32 evaluable subjects, in the same manner as the first.

## 12.3 2ND PRIMARY OBJECTIVE

For the second primary objective, which is to determine if the current CTC-ETI algorithm is associated with rapid progression, defined as progression according to RECIST v1.1 criteria or death due to MBC within 3 months of starting a new ET, in subjects with ER positive MBC starting a new ET (clinical validity), the association of the CTC-ETI with rapid progression will be evaluated. All subjects in whom baseline CTC-ETI is successfully calculated and progression status within 3 months of starting ET can be ascertained will be

PRODUCTS ARE FOR RESEARCH USE ONLY AND THEIR PERFORMANCE CHARACTERISTICS ON CLINICAL SAMPLES HAVE NOT BEEN DETERMINED

Janssen Diagnostics, LLC
700 Route 202 South
Raritan, NJ 08869

considered evaluable and analyzed (i.e., evaluable for clinical validity analyses). The target sample size for assessing clinical validity is 120 of 200 enrolled subjects. The conservative assumptions for the sample size and power considerations are:

- Approximately 15% of enrolled subjects will not have a baseline sample evaluated because of non-draw or shipping/handling issues (i.e. short draw, blood clotted, etc.);
- CTC-ETI will be sucessfully performed on baseline blood draw in 80% of subjects enrolled;
- Approximately 10% of subjects with an evaluable baseline CTC-ETI will be non-evaluable due to other reasons (i.e. protocol violation, unable to determine rapid progression status, etc.);
- CTC-ETI Scores will be distributed such that 30%, 40% and 30% of subjects have low, intermediate, and high CTC-ETI Scores, respectively;
- Approximately 20%, 34%, and 75% of subjects having low, intermediate, and high CTC-ETI Scores (42% of subjects overall) will have evidence of rapid progression, respectively.

The calculations are based upon simulations that further assume uniform enrollment of ~11 subjects per month (200 subjects over 1.5 years), maximum follow-up of 12 months and administrative censoring at 3 months after the last subject is enrolled, and an exponential distribution of progression times, so that an estimated 51 rapid progression events (42%) are observed among 120 subjects. In this scenario, there is >95% power for the primary test of association of CTC-ETI categories with rapid progression (Fisher's exact test for 3x2 table with 2-sided $\alpha=0.05$); and power remains around 95% if only 65% (rather than 75%) of subjects with high CTC-ETI have rapid progression (39% of subjects overall). The sample size further provides sufficient power for assessing sensitivity and specificity of high vs. low/intermediate CTC-ETI. If we aim to have 90% specificity (10% false-positive fraction) and 80% sensitivity, and would like to show that CTC-ETI has at least 75% specificity and 60% sensitivity, then there is 89% power based on a 90% rectangular confidence region using 1-sided exact confidence limits [37].

The CTC-ETI distribution will be summarized descriptively, and CTC-ETI categories (low/intermediate/high) tabulated with two-sided 95% exact binomial CIs. The proportion of subjects with rapid progression, with two-sided 95% exact binomial CIs, will be tabulated according to CTC-ETI categories (low/intermediate/high) and Fisher's exact test, as extended for a 3x2 table, will assess the global association of CTC-ETI with rapid progression. An exact two-sided p-value will be reported for $\alpha=0.05$ level test. The odds ratios for intermediate versus low and for high versus low CTC-ETI will be estimated. In addition, the receiver operating characteristic (ROC) curve for the continuous CTC-ETI Scores (range 0-16) will be estimated, with focus on the characteristics of the intermediate and high cutpoints summarized including sensitivity, specificity and area under the ROC curve. Other measures of classification (discrimination) accuracy (e.g., net re-classification index) may also be estimated.

PRODUCTS ARE FOR RESEARCH USE ONLY AND THEIR PERFORMANCE CHARACTERISTICS ON CLINICAL SAMPLES HAVE NOT BEEN DETERMINED

Janssen Diagnostics, LLC
700 Route 202 South
Raritan, NJ 08869

## 12.4 3RD PRIMARY OBJECTIVE

For the third primary objective, which is to refine the CTC-ETI algorithm (which is currently based on crude assumptions) using clinical outcomes data to identify subjects with rapid progression, we will further assess the performance of the CTC-ETI categories and the development of its scoring. All subjects in whom baseline CTC-ETI is successfully calculated and progression status within 3 months of starting ET can be ascertained will be considered evaluable and will be analyzed (i.e., evaluable for clinical validity analyses as in Section 12.3). The objective is to refine the use of the 5 characteristics of CTCs (count, % CTC positive for each of the 4 markers) to classify subjects with rapid progression versus others. In addition to logistic regression with ROC-based methods for classification, recursive partitioning and logic regression will be used to refine the algorithm for calculating CTC-ETI. Resampling methods will be used to assess robustness of specific aspects of the algorithm and to internally validate the refined CTC-ETI Score.

As described for the 2nd Primary Objective, the target is at least 51 (42%) rapid progression events among an estimated sample size of 120 subjects evaluable for clinical validity. For power to evaluate the continuous refined CTC-ETI Score, we consider a fixed acceptable sensitivity of 70% and estimate the corresponding false-positive fraction and test whether it is below an acceptable null value based on the false-positive fraction CI at the fixed value sensitivity. That is, we desire 95% specificity but must be at least 80% specific at the threshold corresponding to 70% sensitivity, and there is 89% power for a 1-sided test based on the upper bound of the 90% confidence limit [37]. In 3 scenarios of wherein 80% sensitivity were deemed acceptable, or the proportion of subjects with rapid progression is ±10% of our 42% estimate, power for specificity remains >85% for each scenario.

## 12.5 SECONDARY OBJECTIVES

The study will assess if a CTC-ETI can be accurately determined at serial time points during treatment. As in the 1st Primary Objective concerning analytical validity, separately according to timepoint, reasons for non-evaluability of any sample will be described and for the evaluable subjects, the number (%) with a successful CTC-ETI calculation, with exact binomial 95% CI, will be determined.

The study will assess the association of CTC-ETI with PFS (both for the current CTC-ETI and the refined CTC-ETI). The analysis will follow as in the 2nd Primary Objective, using the logrank test for the primary hypothesis test; Kaplan-Meier estimates of the PFS distribution will also be estimated and hazard ratios for high vs. low and intermediate vs. low CTC-ETI will be estimated from Cox proportional hazards model. Methods for classification appropriate for time-to-event variables will be implemented. At the time of the primary analysis for clinical validity, it is anticipated that about 85 to 90 PFS events will have been observed, and by the same simulations, there is >95% power for a logrank test of global association (2df; 2-sided $\alpha$=0.05) of CTC-ETI with PFS.

We will further explore changes in CTC-ETI during treatment and their association with PFS and rapid progression. Serial CTC-ETI over time will be descriptively and graphically summarized (e.g., with spider plots), both as observed scores and changes. The analysis of

PRODUCTS ARE FOR RESEARCH USE ONLY AND THEIR PERFORMANCE CHARACTERISTICS ON CLINICAL SAMPLES HAVE NOT BEEN DETERMINED

Janssen Diagnostics, LLC
700 Route 202 South
Raritan, NJ 08869

rapid progression will follow similarly to that described above. When looking at association of levels and changes in CTC-ETI with subsequent PFS, a landmark analysis approach will be used, e.g., for all subjects alive and free of progression at 1 month with CTC-ETI successfully measured, the association of 1m CTC-ETI (or change from baseline to 1m CTC-ETI) with PFS landmarked from the 1 month time point.

The assessment of correlation of baseline CTC markers with corresponding staining in the tissue samples will use descriptive and graphical summaries such as cross-tabulations or scatterplots, and will attempt to take into account the proximity of the CTC assessment to the tissue sample and number of prior lines of ET.

### 12.6 Definitions of Evaluable

Analytical validity (1st Primary Objective): All subjects for whom the baseline blood sample is collected and shipped to the study laboratory without pre-analytical errors (see section 12.8) will be considered evaluable for the purposes of this evaluation.

Clinical validity (2nd and 3rd Primary Objectives): Subjects will be considered evaluable for clinical validity if they have successful calculation of a baseline CTC-ETI and progression status within 3 months of starting ET can be ascertained.

### 12.7 Additional Analyses

The data collected from the BioMarQ software will be collected and evaluated separately, comparing the intensity results to the visual scores for each category of staining (0, 1+, 2+, or 3+) to see if there are distinct thresholds for positive vs. negative marker staining that can be established for each marker and utilized for future studies.

### 12.8 Guidelines for Failure/Futility and Monitoring

Once the study is initiated, periodic monitoring will be performed to determine if there are ongoing issues that would lead to futility of continuing the trial. These include technical problems with the CTC-ETI assays and clinical problems with subject enrollment and/or compliance, or occurrence of endpoints. We will monitor for the following areas of futility that require immediate reparation/revision of the study or closure:

1. Pre-Analytical Errors: Failure to receive necessary amount of blood, clots in the samples, cracks in the tubes, problems regarding shipping procedures, or any other problems before the arrival of the samples in the lab are considered pre-analytical errors. If these errors occurred at the baseline sample, the subject will be considered non-evaluable and these errors will not affect the analytical validity analyses. We will monitor this issue in real time and take corrective action with each clinical investigational site as we recognize a problem.

2. Analytical futility will be evaluated after the first approximately 35-40 subjects have been enrolled, again after approximately 70–80 subjects have been enrolled, and upon completion of the enrollment (unless the trial is stopped earlier). IF >20% OF THE SAMPLES PROCESSED HAVE UNRESOLVED TECHNICAL FAILURES,

ACCRUAL WILL BE SUSPENDED AND THE TECHNICAL ASPECTS OF THE ASSAY WILL BE RE-EVALUATED. Unresolved Technical Failures will be defined as follows (see **Appendix E** for a more detailed description of Unresolved Technical Failures):

- Reagent and Instrument failures: Sample aborts on AutoPrep, sample not fully transferred to the cartridge, control failures, no CTC are identified and ferrofluid is left in the Autoprep tube, etc.
- Unsatisfactory sample quality and results: Sample unable to be scanned, unsatisfactory results due to interfering substances, inability to interpret marker results, etc.
- Analytical failures: Irresolvable discordance in CTC count in one or more of the four aliquots, making the CTC-ETI calculation unsuccessful, as described in "Considerations for Calculation of CTC-ETI" under **Appendix E**.

If a technical error occurs but does not affect the analysis of the sample, this is not considered a technical failure (for example if a fluid detection error is experienced, but does not result in a sample abortion, this should be noted but will not be considered a technical failure since the sample was evaluable).

3. Laboratory Errors: Errors made by the laboratory (such as failure to process a sample within 96Hr after collection if received within 96Hr after collection, failure to scan a cartridge within 24Hr after processing, use of expired reagents, etc.) will not be counted as an unresolved technical error, but they will be monitored on a monthly basis. Retraining of the laboratory personnel will be done as appropriate. See **Appendix E** for a more detailed description of Laboratory Errors.

4. Poor enrollment. We will monitor enrollment on a monthly basis and make pragmatic decisions regarding ongoing accrual and/or revisions to the protocol, as deemed appropriate.

5. Poor adherence to protocol stipulation (follow-up tests, adherence to treatment, etc). We will monitor this issue in real time and take corrective action with each clinical investigational site as we recognize a problem.

6. Discordance with initial assumptions will be evaluated after the first approximately 35-40 subjects have been enrolled, again after approximately 70–80 subjects have been enrolled, and upon completion of the enrollment (unless the trial is stopped earlier). The following criteria will be used to suspend accrual for re-evaluation of the initial assumptions:
   - Based on proportion of subjects with elevated CTC/7.5mL WB:
     - If ≤25% of the baseline samples evaluable for CTC-ETI have elevated CTC levels (i.e. ≥5 CTC/7.5mL).
     - If ≤15% of the baseline samples evaluable for CTC-ETI have high CTC-ETI Scores.
   - Based on proportion of subjects who comply with first follow-up imaging schedule:

Janssen Diagnostics, LLC
700 Route 202 South
Raritan, NJ 08869

- If ≥25% of enrolled subjects fail to have follow-up imaging studies at 3 months (i.e., progression status within 3 months of starting ET cannot be ascertained).

# 13.0 REGULATORY/ETHICAL REQUIREMENTS

## 13.1 INVESTIGATOR RESPONSIBILITIES:

The investigator is responsible for ensuring that the study is performed in accordance with the protocol, current International Conference on Harmonization (ICH) guidelines on Good Clinical Practice (GCP), and applicable regulatory and country-specific requirements.

Good Clinical Practice is an international ethical and scientific quality standard for designing, conducting, recording, and reporting studies that involve the participation of human subjects. Compliance with this standard provides public assurance that the rights, safety, and well-being of study subjects are protected, consistent with the principles that originated in the Declaration of Helsinki, and that the study data are credible.

- Each site's Principal Investigator must obtain IRB approval for the Protocol and Consent Form prior to enrolling subjects in this study, and must obtain IRB approval for any amendments to the protocol as necessary.
- Each site's Principal Investigator must report any unanticipated adverse device effects to the sponsor and the IRB within 10 days of its occurrence.
- Each site's Principal Investigator must obtain written Informed Consent for all subjects participating in the study.
- Each site's Principal Investigator must allow FDA investigators to inspect the records of the study.
- Each site's Principal Investigator must ensure that subjects are enrolled according to the Inclusion/Exclusion criteria listed in Section 6, and that all subject information on Informed Consent Forms, Screening/Enrollment Logs, and Case Report Forms is complete and accurate.
- It is the responsibility of each site's Investigators and Study Coordinators to ensure that, to the best of their knowledge, all subject information is complete and accurate. Therefore, CRFs may be signed by the site's Principal Investigator or by the Study Coordinator to verify that all forms are accurate and complete.

## 13.2 INDEPENDENT ETHICS COMMITTEE OR INSTITUTIONAL REVIEW BOARD

Before the start of the study, the Principal Investigator (or Sponsor where required) will provide the IEC/IRB with current and complete copies of the following documents:

- Final protocol and, if applicable, amendments
- Sponsor-approved informed consent form (and any other written materials to be provided to the subjects)

Janssen Diagnostics, LLC
700 Route 202 South
Raritan, NJ 08869

- Sponsor-approved subject recruiting materials (if applicable)
- Information on compensation for study-related injuries or payment to subjects for participation in the study (if applicable)
- Investigator's curriculum vitae or equivalent information (unless not required, as documented by the IEC/IRB)
- Information regarding funding, name of the sponsor, institutional affiliations, other potential conflicts of interest, and incentives for subjects
- Any other documents that the IEC/IRB requests to fulfill its obligation

This study will be undertaken only after the IEC/IRB has given full approval of the final protocol, amendments (if any), the informed consent form, applicable recruiting materials, and subject compensation programs, and the Sponsor has received a copy of this approval. This approval letter must be dated and must clearly identify the IEC/IRB and the documents being approved.

During the study the Principal Investigator (or sponsor where required) will send the following documents and updates to the IEC/IRB for their review and approval, where appropriate:

- Protocol amendments
- Revision(s) to informed consent form and any other written materials to be provided to subjects
- If applicable, new or revised subject recruiting materials approved by the Sponsor
- Revisions to compensation for study-related injuries or payment to subjects for participation in the study, if applicable
- Summaries of the status of the study at intervals stipulated in guidelines of the IEC/IRB (at least annually)
- Reports of adverse events that are serious, unlisted/unexpected, and associated with the collection of the study related blood samples
- New information that may adversely affect the safety of the subjects or the conduct of the study
- Deviations from or changes to the protocol to eliminate immediate hazards to the subjects
- Report of deaths of subjects under the investigator's care
- Notification if a new investigator is responsible for the study at the site
- Any other requirements of the IEC/IRB

For all protocol amendments (excluding the ones that are purely administrative, with no consequences for subjects, data or study conduct), the amendment and applicable informed

Janssen Diagnostics, LLC
700 Route 202 South
Raritan, NJ 08869

consent form revisions must be submitted promptly to the IEC/IRB for review and approval before implementation of the change(s).

At least once a year, the IEC/IRB will be asked to review and reapprove this study. The re-approval should be documented in writing (excluding the ones that are purely administrative, with no consequences for subjects, data, or study conduct).

At the end of the study, the Principal Investigator (or sponsor where required) will notify the IEC/IRB about the study completion.

### 13.3 Informed Consent

Each subject must give written consent according to local requirements after the nature of the study has been fully explained. The consent form must be signed before performance of any study-related activity. The consent form that is used must be approved by both the Sponsor and by the reviewing IEC/IRB and be in a language that the subject can read and understand. The informed consent should be in accordance with principles that originated in the Declaration of Helsinki, current ICH and GCP guidelines, applicable regulatory requirements, and Sponsor policy.

Before enrollment in the study, the investigator or an authorized member of the study-site personnel must explain to potential subjects the aims, methods, reasonably anticipated benefits, and potential hazards of the study, and any discomfort participation in the study may entail. Subjects will be informed that their participation is voluntary and that they may withdraw consent to participate at any time. They will be informed that choosing not to participate will not affect the care the subject will receive for the treatment of his or her disease. They must be informed of their rights as subjects, know that their participation is voluntary, and that the results of the tests will not be made available to them or their physician for treatment purposes. The consent form must specify whom to contact if there are injuries as a result of the study, and whom to contact with general questions about the trial. Subjects must also be notified that various components isolated from their blood samples (i.e. CTC positive cartridges) may be fixed, frozen and stored for future research purposes, but that if they choose to withdraw from the study at any time, the stored samples will be destroyed if requested in writing. Finally, they will be told that the investigator will maintain a subject identification register for the purposes of long-term follow up if needed and that their records may be accessed by health authorities and authorized sponsor personnel without violating the confidentiality of the subject, to the extent permitted by the applicable law(s) or regulations. By signing the informed consent form the subject is authorizing such access, including permission to obtain information about his or her survival status.

The subject will be given sufficient time to read the informed consent form and the opportunity to ask questions. After this explanation and before entry into the study, consent should be appropriately recorded by means of the subject's personally dated signature. After having obtained the consent, a copy of the informed consent form must be given to the subject.

Janssen Diagnostics, LLC
700 Route 202 South
Raritan, NJ 08869

A sample Informed Consent is provided in **Appendix A** of this Protocol, and may be used as is or modified by the IEC/IRB as deemed necessary. The Sponsor must approve any changes made to the sample Informed Consent Form.

### 13.4 LONG-TERM STORAGE OF SAMPLES FOR FUTURE RESEARCH

Cartridges containing ≥5 CTC, which have been fixed and dried (Section 8.9) will be labeled with the Sample ID and a unique Cartridge ID, and these cartridges will be stored frozen (-20°C) for up to 10 years. A listing of the stored cartridges will be maintained and will include the Sample ID, the Cartridge ID, the sample collection date, the date the sample was stored frozen, and the storage location information (i.e. freezer, shelf, drawer, box). Likewise, some of the cartridges containing 1-4 CTC or cartridges containing ≥5 CTC will be labeled with the Sample ID and a unique Cartridge ID, and will be stored at (4°C) for up to 10 years. A listing of the stored cartridges will be maintained and will include the Sample ID, the Cartridge ID, the sample collection date, the date the sample was stored, and the storage location information (i.e. refrigerator, shelf, drawer, box). The listing will also include information about the status of the cartridge (i.e. tested and destroyed, withdrawn and destroyed, stored, etc.).

Tissue samples (blocks and/or slides) will be stored at room temperature for up to 10 years. A listing of the stored tissue samples will be maintained (**Appendix B**) and will include the Sample ID, the sample type (block or slides), the number of blocks/slides, and the storage location information (i.e. shelf, drawer, box, position(s)). The listing will also include information about the status of the tissue sample (i.e. number of blocks/slides tested and destroyed, withdrawn and destroyed, stored, etc.).

## 14.0 ADMINISTRATIVE REQUIREMENTS

### 14.1 MODIFICATION OF THE PROTOCOL (AMENDMENTS)

Neither the Principal Investigator nor the Sponsor will modify this protocol without a formal amendment by the Sponsor. All protocol amendments must be issued by the Sponsor, and signed and dated by the Principal Investigator. Protocol amendments must not be implemented without prior IEC/IRB approval, or when the relevant competent authority has raised any grounds for non-acceptance, except when necessary to eliminate immediate hazards to the subjects, in which case the amendment must be promptly submitted to the IEC/IRB and relevant competent authority. Documentation of amendment approval by the investigator and IEC/IRB must be provided to the Sponsor or its designee. When the change(s) involves only logistic or administrative aspects of the study, the IRB (and IEC where required) only needs to be notified.

During the course of the study, in situations where a departure from the protocol is unavoidable, the Principal Investigator or other physician in attendance will contact the appropriate Sponsor representative (see Contact Information page(s) provided separately). Except in emergency situations, this contact should be made before implementing any departure from the protocol. In all cases, contact with the Sponsor must be made as soon as possible to discuss the situation and agree on an appropriate course of action. The data

Janssen Diagnostics, LLC
700 Route 202 South
Raritan, NJ 08869

recorded in the CRF and source documents will reflect any departure from the protocol, and the source documents will describe this departure and the circumstances requiring it.

### 14.2 Required Pre-Study Documentation

The following administrative information must be placed in the site Regulatory Binder and copies provided to the Sponsor prior to study initiation:

- Protocol and amendment(s), if any, signed and dated by the Principal Investigator
- A copy of the dated and signed, written IEC/IRB approval of the protocol, amendments, informed consent form, any recruiting materials, and if applicable, subject compensation programs. This approval must clearly identify the specific protocol by title and number and must be signed by the IEC/IRB chairman or authorized designee.
- Name and address of the IEC/IRB, including a current list of the IEC/IRB members and their function, with a statement that it is organized and operates according to GCP and the applicable laws and regulations. If accompanied by a letter of explanation, or equivalent, from the IEC/IRB, a general statement may be substituted for the IEC/IRB membership list. If an investigator or a member of the study-site personnel is a member of the IEC/IRB, documentation must be obtained to state that this person did not participate in the deliberations or in the vote/opinion of the study.
- Signed and dated statement of investigator (eg, Form FDA 1572), if applicable.
- Documentation of Principal Investigator and all sub-investigator qualifications (eg, curriculum vitae and copy of medical license).
- Completed financial disclosure form from the Principal Investigator and all sub-investigators.
- Signed and dated clinical trial agreement, which includes the financial agreement.
- Signature Log.
- Any other documentation required by local regulations.

In addition to the Regulatory Binder, each site will receive Case Report Forms prior to study start-up. Copies of the Regulatory Binder are to be maintained at the study sites and by the Sponsor.

### 14.3 Subject Identification, Enrollment, and Screening Logs

The Principal Investigator agrees to complete a screening / enrollment log (**Appendix B**) to permit easy identification of each subject during and after the study. This document will be reviewed by the Sponsor's study site contact for completeness.

The subject screening / enrollment log will be treated as confidential and will be filed by the Principal Investigator in the study file. To ensure subject confidentiality, no copy of the subject screening / enrollment log will be made. All reports and communications relating to the study will identify subjects by site assigned Subject ID numbers from the enrollment log.

Janssen Diagnostics, LLC
700 Route 202 South
Raritan, NJ 08869

The investigator will include all subjects who were seen to determine eligibility for inclusion in the study on the screening / enrollment log, including those not screened who were not eligible for enrollment.

### 14.4 Source Documentation

At a minimum, source documentation must be available for the following to confirm data collected in the CRF: subject identification, eligibility, and study identification; study discussion and date of informed consent; dates of visits; results of safety and efficacy parameters as required by the protocol; record of all adverse events and follow-up of adverse events; concomitant medication; study drug administration information; and date of study completion and reason for early discontinuation of study drug or withdrawal from the study, if applicable. In addition, the author of an entry in the source documents should be identifiable.

At a minimum, the type and level of detail of source data available for a study subject should be consistent with that commonly recorded at the study site as a basis for standard medical care. Specific details required as source data for the study will be reviewed with the investigator before the study and will be described in the monitoring guidelines (or other equivalent document).

### 14.5 Record Retention

In compliance with the ICH/GCP guidelines, the investigator/institution will maintain all CRFs and all source documents that support the data collected from each subject, as well as all study documents as specified in ICH/GCP Section 8, Essential Documents for the Conduct of a Clinical Trial, and all study documents as specified by the applicable regulatory requirement(s). The investigator/institution will take measures to prevent accidental or premature destruction of these documents.

Essential documents must be retained until at least 2 years after the last approval of a marketing application in an ICH region and until there are no pending or contemplated marketing applications in an ICH region or until at least 2 years have elapsed since the formal discontinuation of clinical development of the investigational product. These documents will be retained for a longer period if required by the applicable regulatory requirements or by an agreement with the Sponsor. It is the responsibility of the Sponsor to inform the investigator/institution as to when these documents no longer need to be retained.

If the responsible investigator retires, relocates, or for other reasons withdraws from the responsibility of keeping the study records, custody must be transferred to a person who will accept the responsibility. The Sponsor must be notified in writing of the name and address of the new custodian. Under no circumstance shall the investigator relocate or dispose of any study documents before having obtained written approval from the Sponsor.

For CRFs completed on pressure-sensitive paper, a copy is to be retained in the archives of the Sponsor. A second copy must be archived by the investigator.

If it becomes necessary for the Sponsor or the appropriate regulatory authority to review any documentation relating to this study, the investigator must permit access to such reports.

### 14.6 MONITORING

The Sponsor may perform on-site monitoring visits as frequently as necessary. The sites will be given ample notification of the visit and will occur at the site's convenience. The monitor will record dates of the visits in a study site visit log that will be kept at the study site. At these visits, the monitor will compare the data entered into the CRFs with the hospital or clinic records (source documents). The nature and location of all source documents will be identified to ensure that all sources of original data required to complete the CRF are known to the Sponsor and study site personnel and are accessible for verification by the Sponsor's study site contact. If electronic records are maintained at the study site, the method of verification must be discussed with the study site personnel.

Direct access to source documentation (medical records) must be allowed for the purpose of verifying that the data recorded in the CRF are consistent with the original source data. Findings from this review of CRFs and source documents will be discussed with the study site personnel. The Sponsor expects that, during monitoring visits, the relevant study site personnel will be available, the source documentation will be accessible, and a suitable environment will be provided for review of study-related documents. If a monitoring visit is conducted, feedback will be provided back to the site via a monitoring report, which must be filed in the study binder.

Since this is not a treatment directed study, nor is it randomized, there will be no independent Data Safety Monitoring Committee. Decisions to close the study on futility measures (Sections 12.7) will be made by the Sponsor, Statistician, and Principal Investigator.

## 15.0 STUDY COMPLETION AND TERMINATION

### 15.1 STUDY COMPLETION

The study is considered completed with the last visit for the last subject participating in the study. The final data from the study site will be sent to the Sponsor (or designee) after completion of the final subject visit at that study site within one month of the last visit for the last subject.

### 15.2 STUDY TERMINATION

The Sponsor reserves the right to close the study site or terminate the study at any time for any reason at the sole discretion of the Sponsor. All study sites will be closed upon study completion. A study site is considered closed when all required documents and study supplies have been collected and a study site closure visit has been performed.

The Principal Investigator may initiate study site closure at any time, provided there is reasonable cause and sufficient notice is given in advance of the intended termination.

Reasons for the early closure of a study site by the Sponsor or investigator may include but are not limited to:

Janssen Diagnostics, LLC
700 Route 202 South
Raritan, NJ 08869

- Failure of the investigator to comply with the protocol, the requirements of the IEC/IRB or local health authorities, the Sponsor's procedures, or GCP guidelines
- Inadequate recruitment of subjects by the investigator
- Discontinuation of study for futility reasons

Janssen Diagnostics, LLC
700 Route 202 South
Raritan, NJ 08869

## 16.0 INVESTIGATOR STATEMENT AND AGREEMENT

I have read this protocol and agree that it contains all necessary details for carrying out this study. I will conduct the study as outlined herein and will complete the study within the time designated.

I will provide copies of the protocol and all pertinent information to all individuals responsible to me who assist in the conduct of this study. I will discuss this material with them to ensure that they are fully informed regarding the study device, the conduct of the study, and the obligations of confidentiality.

I will conduct the study in compliance with the protocol, applicable regulations, and institutional policy, keep the protocol and all study related materials confidential, and discontinue the study at the Sponsor's request.

**Site Representative (where required):**

Name (typed or printed): ______________________________

Institution and Address: ______________________________

______________________________

______________________________

______________________________

Signature: ______________________________ Date: ______________
(Day Month Year)

**Principal (Site) Investigator:**

Name (typed or printed): ______________________________

Institution and Address: ______________________________

______________________________

______________________________

______________________________

Telephone Number: ______________________________

Signature: ______________________________ Date: ______________
(Day Month Year)

Janssen Diagnostics, LLC
700 Route 202 South
Raritan, NJ 08869

# 17.0 REFERENCES


1. Ellis M, Hayes DF, M.E. L. Treatment of Metastatic Breast Cancer. In: Harris J, Lippman M, Morrow M, Osborne CK, eds Diseases of the Breast 3rd ed. Philadelphia: Lippcott Williams & Wilkins; 2004:1101-1162.
2. Early Breast Cancer Trialists' Collaborative Group (EBCTCG). Effects of chemotherapy and hormonal therapy for early breast cancer on recurrence and 15-year survival: an overview of the randomised trials. Lancet 2005;365:1687-1717.
3. Howell A, Robertson JF, Abram P, et al. Comparison of fulvestrant versus tamoxifen for the treatment of advanced breast cancer in postmenopausal women previously untreated with endocrine therapy: a multinational, double-blind, randomized trial. J Clin Oncol 2004;22:1605-1613.
4. Osborne CK, Pippen J, Jones SE, et al. Double-blind, randomized trial comparing the efficacy and tolerability of fulvestrant versus anastrozole in postmenopausal women with advanced breast cancer progressing on prior endocrine therapy: results of a north american trial. J Clin Oncol 2002;20:3386-3395.
5. Klijn JG, Beex LV, Mauriac L, et al. Combined treatment with buserelin and tamoxifen in premenopausal metastatic breast cancer: a randomized study. J Natl Cancer Inst 2000;92:903-911.
6. Baselga J, Campone M, Piccart M, et al. Everolimus in Postmenopausal Hormone-Receptor-Positive Advanced Breast Cancer. N Engl J Med 2012;366:520-529.
7. Hayes DF, Smerage J. Is there a role for circulating tumor cells in the management of breast cancer? Clin Cancer Res 2008;14:3646-3650.
8. Cristofanilli M, Budd GT, Ellis MJ, et al. Circulating tumor cells, disease progression, and survival in metastatic breast cancer. N Engl J Med 2004;351:781-791.
9. Cohen SJ, Punt CJ, Iannotti N, et al. Relationship of circulating tumor cells to tumor response, progression-free survival, and overall survival in patients with metastatic colorectal cancer. J Clin Oncol 2008;26:3213-3221.
10. de Bono JS, Scher HI, Montgomery RB, et al. Circulating tumor cells predict survival benefit from treatment in metastatic castration-resistant prostate cancer. Clin Cancer Res 2008;14:6302-6309.
11. Paoletti C, Connelly MC, Chianese D, et al. Multi-parameter molecular characterization of circulating tumor cells (CTC): Development of a CTC-Endocrine Therapy Index (CTC-ETI). Proc Am Assoc Cancer Res 2011.
12. Paoletti C, Connelly M, Chianese D, et al. Development of Circulating Tumor Cell-Endocrine Therapy Index in Metastaic Breast Cancer Patients. Cancer Research 2011;71 (24 Suppl.):451 s; abs. P4-07-16.
13. Jensen EV. Hormone dependency of breast cancer. Cancer 1981;47:2319-2326.


PRODUCTS ARE FOR RESEARCH USE ONLY AND THEIR PERFORMANCE CHARACTERISTICS ON CLINICAL SAMPLES HAVE NOT BEEN DETERMINED

Janssen Diagnostics, LLC
700 Route 202 South
Raritan, NJ 08869

14. Elledge RM, Green S, Pugh R, et al. Estrogen receptor (ER) and progesterone receptor (PgR), by ligand-binding assay compared with ER, PgR and pS2, by immuno-histochemistry in predicting response to tamoxifen in metastatic breast cancer: a Southwest Oncology Group Study. Int J Cancer 2000;89:111-117.

15. Johnston SR, MacLennan KA, Sacks NP, et al. Modulation of Bcl-2 and Ki-67 expression in oestrogen receptor-positive human breast cancer by tamoxifen. Eur J Cancer 1994;30A:1663-1669.

16. Elledge RM, Green S, Howes L, et al. bcl-2, p53, and response to tamoxifen in estrogen receptor-positive metastatic breast cancer: a Southwest Oncology Group study. J Clin Oncol 1997;15:1916-1922.

17. Paik S, Shak S, Tang G, et al. A multigene assay to predict recurrence of tamoxifen-treated, node-negative breast cancer. N Engl J Med 2004;351:2817-2826.

18. Yamauchi H, Stearns V, Hayes DF. When is a tumor marker ready for prime time? A case study of c-erbB-2 as a predictive factor in breast cancer. J Clin Oncol 2001;19:2334-2356.

19. Shou J, Massarweh S, Osborne CK, et al. Mechanisms of tamoxifen resistance: increased estrogen receptor-HER2/neu cross-talk in ER/HER2-positive breast cancer. J Natl Cancer Inst 2004;96:926-935.

20. Wright C, Nicholson S, Angus B, et al. Relationship between c-erbB-2 protein product expression and response to endocrine therapy in advanced breast cancer. Br J Cancer 1992;65:118-121.

21. Dowsett M, Smith IE, Ebbs SR, et al. Short-term changes in Ki-67 during neoadjuvant treatment of primary breast cancer with anastrozole or tamoxifen alone or combined correlate with recurrence-free survival. Clin Cancer Res 2005;11:951s-958s.

22. Colozza M, Azambuja E, Cardoso F, et al. Proliferative markers as prognostic and predictive tools in early breast cancer: where are we now? Ann Oncol 2005;16:1723-1739.

23. Yerushalmi R, Woods R, Ravdin PM, et al. Ki67 in breast cancer: prognostic and predictive potential. Lancet Oncol 2010;11:174-183.

24. Cheang MC, Chia SK, Voduc D, et al. Ki67 index, HER2 status, and prognosis of patients with luminal B breast cancer. J Natl Cancer Inst 2009;101:736-750.

25. Albain KS, Barlow WE, Shak S, et al. Prognostic and predictive value of the 21-gene recurrence score assay in postmenopausal women with node-positive, oestrogen-receptor-positive breast cancer on chemotherapy: a retrospective analysis of a randomised trial. Lancet Oncol 2010;11:55-65.

26. Paik S, Tang G, Shak S, et al. Gene expression and benefit of chemotherapy in women with node-negative, estrogen receptor-positive breast cancer. J Clin Oncol 2006;24:3726-3734.

27. Hayes DF, Bast RC, Desch CE, et al. Tumor marker utility grading system: a framework to evaluate clinical utility of tumor markers. J Natl Cancer Inst 1996;88:1456-66.

PRODUCTS ARE FOR RESEARCH USE ONLY AND THEIR PERFORMANCE CHARACTERISTICS ON CLINICAL SAMPLES HAVE NOT BEEN DETERMINED

PROPERTY OF JANSSEN, LLC ALL INFORMATION IS CONFIDENTIAL

28. Recommendations from the EGAPP Working Group: can tumor gene expression profiling improve outcomes in patients with breast cancer? Evaluation of Genomic Applications in Practice and Prevention (EGAPP) Working Group. Genetics in Medicine 2009;11:66-73.

29. Eisenhauer EA, Therasse P, Bogaerts J, et al. New response evaluation criteria in solid tumors: Revised RECIST guideline (version 1.1). Eur J Cancer 2009;45:228-247.

30. Wolff AC, Hammond ME, Schwartz JN, et al. American Society of Clinical Oncology/College of American Pathologists guideline recommendations for human epidermal growth factor receptor 2 testing in breast cancer. J Clin Oncol 2007;25:118-45.

31. Oken, M.M., Creech, R.H., Tormey, D.C., et al. Toxicity and response criteria of the Eastern Cooperative Oncology Group. Am J Clin Oncol 1982;5:649-655.

32. Parker JS, Mullins M, Cheang MC, et al. Supervised risk predictor of breast cancer based on intrinsic subtypes. J Clin Oncol 2009;27:1160-1167.

33. Paik S, Shak S, Tang G, et al. A multigene assay to predict recurrence of tamoxifen-treated, node-negative breast cancer. N Engl J Med 2004;351:2817-2826.

34. Albain KS, Barlow WE, Shak S, et al. Prognostic and predictive value of the 21-gene recurrence score assay in postmenopausal women with node-positive, oestrogen-receptor-positive breast cancer on chemotherapy: a retrospective analysis of a randomised trial. Lancet Oncol 2010;11:55-65.

35. Harvey JM, Clark GM, Osborne CK, Allred DC. Estrogen receptor status by immunohistochemistry is superior to the ligand-binding assay for predicting response to adjuvant endocrine therapy in breast cancer. J Clin Oncol 1999;17:1474-1481.

36. Lucas DR, Shukla A, Thomas DG, et al. Dedifferentiated liposarcoma with inflammatory myofibroblastic tumor-like features. Am J Surg Pathol 2010;34:844-851.

37. Pepe MS, Feng Z, Janes H, et al. Pivotal evaluation of the accuracy of a biomarker used for classification or prediction: standards for study design. J Natl Cancer Inst 2008;100:1432-1438.

Janssen Diagnostics, LLC
700 Route 202 South
Raritan, NJ 08869

# APPENDIX A

## Example Informed Consent Form

# Research Subject Information and Consent Form

**Study Sponsor:** **Janssen Diagnostics, LLC**

*(Janssen Diagnostics, LLC is part a company of Johnson & Johnson)*

---

**Protocol Number:** COMETI-P2-2012.0

**Protocol Title:** COMETI Phase 2: Characterization of Circulating Tumor Cells from Subjects with Metastatic Breast Cancer Using the CTC-Endocrine Therapy Index

**Simplified Title:** COMETI Phase 2

**Study Doctor:** [Insert Principal Investigator name]

**Study Staff:** [Insert Sub-Investigator name(s)]

**Study Site(s):** [Insert Site Name]
[Insert Address (Line 1)]
[Insert Address (Line 2)]
[Insert Address (Line 3), if applicable]
[Insert City, State Zip Code]

**24 HR Telephone #:** [Insert phone number]

---

**Please read this document carefully**

You are being invited to take part in a clinical trial, a type of research study. Taking part in this study is voluntary. Before you decide, you should know why the research is being done and what it involves. Please read this consent form carefully, as it may contain words that you do not understand. Ask the study doctor (or his or her staff) to explain any words or information that you do not clearly understand as well as any other questions you may have. You may take an unsigned copy of this form home with you to read again. Take your time to think and talk about it with your family and friends before making your decision.

## WHY IS THIS STUDY BEING DONE?

Your doctor has determined that you have metastatic breast cancer, which means that it has spread or recurred outside of your breast (such as in your bones or liver or lungs or elsewhere). Your cancer makes a biomarker called the estrogen receptor (ER), which means your cancer is reasonably likely to respond to anti-estrogen, also called “endocrine”, therapies. However, not everyone with ER positive breast cancer responds to endocrine therapies. Such patients might be better treated with chemotherapy even though their cancer is ER positive. Although chemotherapy might have more side effects, if endocrine therapy is unlikely to work, chemotherapy is more likely to make the patient feel better and perhaps live longer than endocrine therapy.

The existence of circulating tumor cells (CTC) in the blood of people with cancer has been known for many years and there has been much interest in their presence in blood and finding out what role they play in the disease process. The purpose of this experimental study is to determine the feasibility of investigating CTC and the expression of several biomarkers on/in the tumor cells that can be detected in blood of patients with metastatic breast cancer. We speculate that the combination of CTC numbers and expression levels of biomarkers (including

ER together with other markers called Bcl-2, HER2, and Ki67) on/in the CTC will tell us if a patient who has ER positive breast cancer is unlikely to benefit from endocrine therapy and would be better treated with chemotherapy.

Using an assay called CELLSEARCH®, we will determine if you have CTC in your blood before you start a new endocrine therapy and at various time intervals during your treatment. For patients who do have elevated CTC in their blood, we will also determine if the CTC are making ER, Bcl-2, HER2, and Ki67, and if the combination of having CTC and what they are making predicts which patients did not benefit from endocrine therapy.

In the case we find cancer cells in your blood, we will look at the molecular make-up of tumor cells in your blood. This make-up includes what kind of genetic differences the cancer cells might have when compared to normal cells, or if the cancer cells have genetic changes that we know are important in the way cancer behaves. We will also study what molecules the cancer might be making. For example, we know that cancer cells make things called RNA and protein that are different from normal cells and "drive" the cancer cell to grow or spread to other places.

We believe that advances in the treatment and prevention of cancer will be based on better understanding of specific molecular and genetic alterations typical of individual tumors. We hope to identify traits that will distinguish which tumors can be treated most successfully by different treatment options.

In order to study the genetics of these cancers, we will study the cells that we are going to collect from your blood and store with your consent. We plan to purify the DNA from these cells and determine if there are any changes in DNA (called mutation, deletion, amplification, or translocation) compared to your normal DNA. In the future, knowing such information might help doctors better choose the right therapy for patients with cancer. By targeting treatment approaches in this way, we hope to improve outcomes while minimizing harmful effects of treatment.

In addition, researchers have found that DNA from a patient's cancer can be found in the patient's blood. This is called circulating cell free DNA (cfDNA). cfDNA from your blood will also be analyzed because both CTC-DNA and cfDNA might be useful to understand your cancer status. In particular, we want to extract the cfDNA from the blood taken from 2 different types of tubes and compare the results.

If you choose to take part in this study, the study doctor for the main study would like to collect a sample of tissue from your previous biopsy. The researchers also ask your permission to store and use your samples and health information for medical research. The research that may be done is unknown at this time. Storing samples for future studies is called "biobanking".

This study will be performed in many centers around the United States and Canada. We do not know if our theory is correct, so we will not use any results from the research performed in this study to direct your care. Rather, your doctor will choose the endocrine therapy most appropriate for you, and you will be followed in a standard manner to determine if it is working or if you should change therapies. However, in the future, our new assay could be important to help doctors decide which treatments are best for individual patients who have ER positive metastatic breast cancer.

An independent ethics committee or institutional review board has approved this research.

## HOW MANY SUBJECTS WILL TAKE PART IN THIS STUDY?

Approximately 200 women with ER positive, HER2 negative breast cancer that has spread to other parts of her body who is going to start a new endocrine therapy will take part in this study over a 2–3 year period.

## WHAT WILL BE DONE IF I VOLUNTEER FOR THIS STUDY?

You are being asked for your permission for the study doctor (or his or her staff) to collect up to 6 tubes of blood (which contain approximately 8mL of blood each when filled, which equals approximately 3-4 tablespoons or 50mL of your blood) at up to five different time points so that it may be used to help develop better diagnostic tools for the management of metastatic breast cancer. This additional blood for research can be taken at the same time as blood is taken for your routine medical evaluations, so you will likely not need to have an extra needle stick or schedule a separate visit to provide these samples for research.

Before you begin the study…
You will need to have one or more of the following exams, tests or procedures to find out if you can be in the study. These exams, tests or procedures are part of regular cancer care and may be done even if you do not join the study. If you have had some of them recently, they may not need to be repeated. This decision will be up to your study doctor.

- Medical History and Assessment of Clinical Status
- Disease Assessment including chest and/or abdomen body images (CT scans or MRI scans) which may also include staging scintigraphy (bone scan or PET scans) or a PET-CT scan that encompasses both.

If you are eligible and agree to participate, you will have blood drawn and sent to a central laboratory for measurement and characterization of circulating tumor cells (CTC) and cfDNA analysis (optional). During the study, you will have blood drawn for CTC assessment and cfDNA analysis (optional) prior to starting your new endocrine therapy (baseline) and at several subsequent time points (1, 2, 3, and 12 months after the initiation of therapy), or at the time your cancer progresses or your doctor stops your therapy, if that is sooner than any of these time points.

Additionally, as routine follow-up for management of your disease and at the discretion of your treating physician, you will have the following evaluations performed at several subsequent time points (anticipated to be every 3 months following the initiation of therapy), or at the time your cancer progresses or your doctor stops your therapy, if that is sooner than any of the subsequent time points:

  – Assessment of Clinical Status (1, 2, 3, 6, 9 and 12 months, or at the time of progression or discontinuation of treatment, whichever occurs first)
  – Disease Assessment, including chest and/or abdomen body imaging (CT scans or MRI scans) which may also include staging scintigraphy (bone scan or PET scans) or a PET-CT scan. [3 and 12 months, or at the time of progression or discontinuation of treatment, whichever occurs first]

- NOTE: CTC can be measured by a standard FDA approved assay. Your physician may wish to use that assay to follow you, at his/her discretion, as a routine part of your care. However, this would be done using the commercially available assay. If you or your doctor feels that measuring CTC is appropriate, the CELLSEARCH® test is commercially available, separate from your participation in this study.

The imaging studies and physician assessments outlined in the study schema below are considered part of your routine clinical care while the medical record review / history, the blood draws for CTC-ETI testing and cell-free DNA (optional), and the optional provision of archival tissue specimens are being done specifically for the purposes of this research study.

***Study Schema***

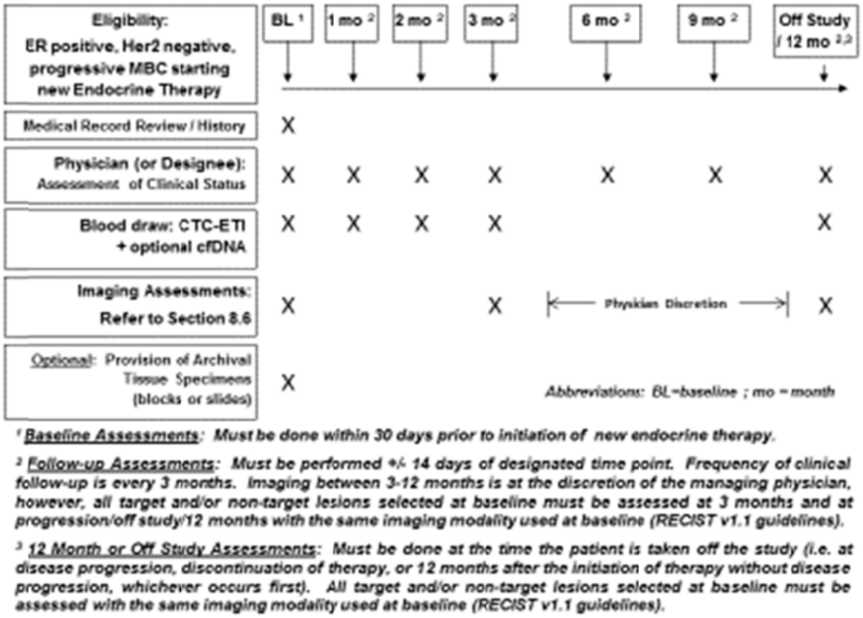

| Eligibility: ER positive, Her2 negative, progressive MBC starting new Endocrine Therapy | BL [1] | 1 mo [2] | 2 mo [2] | 3 mo [2] | 6 mo [2] | 9 mo [2] | Off Study / 12 mo [2,3] |
|---|---|---|---|---|---|---|---|
| Medical Record Review / History | X | | | | | | |
| Physician (or Designee): Assessment of Clinical Status | X | X | X | X | X | X | X |
| Blood draw: CTC-ETI + optional cfDNA | X | X | X | X | | | X |
| Imaging Assessments: Refer to Section 8.6 | X | | | X | ← Physician Discretion | → | X |
| Optional: Provision of Archival Tissue Specimens (blocks or slides) | X | | | | | | |

*Abbreviations: BL=baseline ; mo = month*

[1] Baseline Assessments: *Must be done within 30 days prior to initiation of new endocrine therapy.*

[2] Follow-up Assessments: *Must be performed +/- 14 days of designated time point. Frequency of clinical follow-up is every 3 months. Imaging between 3-12 months is at the discretion of the managing physician, however, all target and/or non-target lesions selected at baseline must be assessed at 3 months and at progression/off study/12 months with the same imaging modality used at baseline (RECIST v1.1 guidelines).*

[3] 12 Month or Off Study Assessments: *Must be done at the time the patient is taken off the study (i.e. at disease progression, discontinuation of therapy, or 12 months after the initiation of therapy without disease progression, whichever occurs first). All target and/or non-target lesions selected at baseline must be assessed with the same imaging modality used at baseline (RECIST v1.1 guidelines).*

After any CTC isolated from the blood collected for the purposes of this research have been analyzed, they may be stored for up to 10 years for future investigational studies as new technology and assays are developed. You will also be asked for your permission to collect part of your previously acquired tissue specimens (from either a prior biopsy and/or surgery) to investigate the same biomarkers in your cancer tissue that we will determine on your CTC. You will not need to have an extra biopsy procedure or surgery done to give a tissue sample for this research.

You have the right to ask us to destroy any sample cartridges that are left over after the study data has been collected. You can do this at any time by contacting the study doctor whose contact information is provided at the end of this form (Principal Investigator). If you ask to do this, the study doctor will destroy any remaining sample cartridges. However, any previously collected data will not be destroyed. The remaining samples will be kept for future analyses. These specimens might be shared at other institutions in the future. However, none of your personal information will be shared. Your test results will be kept completely confidential. The results will be coded, meaning that it will not be possible for anyone else to link your name to your test results, unless you have given your written consent.

We will also attempt to perform a thorough genetic analysis of CTC, including DNA and RNA. Specifically, we might compare the DNA of CTC and your normal cells. We will perform very detailed genetic testing to identify changes that may guide treatment in the future.

Samples may also be stored and used for future studies of genes associated with cancer. Specimens may be distributed to outside investigators without any identifying information that would link a specimen to you as individual person. Some studies will use the biological specimens in genetic analysis looking for molecular markers, genes, and/or proteins that may be used to predict response of specific tumors to specific treatment options. However, these are entirely investigational (experimental), and therefore neither you nor your doctor will be told the results of any testing on your specimens, since we do not know if any of these results are clinically important. Clinical data regarding your disease, treatment status, and response to therapy will be collected from your medical record on an ongoing basis so that biological information that is learned from your specimens can be linked to your response to therapy as well as your treatment outcomes. All of this information will be kept and protected in a locked cabinet or storage unit. Clinical data includes but are not limited to previous cancer history, stage of your tumor, treatment administered (i.e. surgery, chemotherapy, radiation), pathology results and tumor response to these therapies (information about tumor growth, reduction size, response, and so forth). As with the specimens, these data will only be distributed to members of our research team or to outside investigators who have secured IRB (ethics board) approval or exemption.

An optional tube of blood is being collected to perform genetic testing related to breast cancer. Therefore, an additional tube of blood will be drawn into a Streck Cell-Free DNA tube at the same time as the other 5 tubes are drawn for CTC assessment. That optional tube of blood is being collected for future circulating DNA analysis and WBC for germ line DNA analysis. Your rights related to genetic testing are described further down in this document.

Your direct participation in the study will be completed no later than 12 months after enrollment into the study, or sooner if your cancer worsens before 12 months, and we will no longer require that you be seen routinely or have blood specimens collected for the purposes of this research study. Because this is a new test, there may be some unavoidable problems that occur during the testing of your blood samples (i.e. reagent and instrument failures, etc.) which may cause the testing to be unsuccessful. If testing of the blood sample taken prior to the start of your new endocrine therapy is unsuccessful, then your doctor will be notified and your direct participation in the study will be completed immediately (i.e. no additional blood samples or information will be collected from you). Of course, further follow-up and treatment decisions will be made between you and your doctor for the continued management of your disease.

## WHAT ARE THE POSSIBLE RISKS OF THE SAMPLE COLLECTIONS?

This study is expected to have minimal risks, since it involves only blood draws. The blood for this research can be taken at the same time as blood is taken for your routine medical evaluations, so you will not likely need to have an extra needle stick to provide these samples for research, thus there is no additional risk of taking an extra amount of blood for the purposes of this research.

As with any research study, though, there may be additional risks of participating that are unforeseeable or hard to predict. There is minimal risk to you in drawing blood from a vein. During the collection of blood samples, you may experience pain and/or bruising at the needle site. Although rare, localized clot formation and infections may occur. Lightheadedness and/or fainting may also occur during or shortly after the blood draw.

The endocrine treatment you receive will be determined by your doctor, not by the study. The follow-up examinations are important to the study, but are routine follow-up evaluations that are standard of care, and you would most likely have them done anyway. To find out more about the risks of your own treatment ask your doctor.

If information becomes available that might change your decision to participate in the study, or if the way that the research is being conducted changes, this information will be made available to you both verbally and in writing in a timely manner.

## WHAT ARE THE BENEFITS OF TAKING PART IN THE STUDY?

There is no direct or medical benefit to you as a result of your participation in this research study. By taking part however, you may contribute to science and medicine and help define the future clinical usefulness of this laboratory test.

## WHAT IF SOMETHING GOES WRONG?

Your participation in this study requires only the drawing of samples of blood. In the unlikely event of injury or illness resulting from participation in this study, immediate medical treatment will be available to you at no cost to you. The sponsor of the study agrees to reimburse the reasonable and necessary medical expenses not routinely covered by insurance for tests and treatments required if you need medical care because of something that happened to you as a result of the blood collection for this research study. The sponsor will not pay the costs to test or treat a condition or injury that is not related to the collection of blood for this research, or for expenses related to the normal progression of a pre-existing medical condition or an underlying disease. In no event will the sponsor pay for treatment for injury or illness that is not a result of the collection of blood for this research. Further financial compensation will not be available.

By signing this Informed Consent Form you **DO NOT** waive or limit any of your legal rights.

## WHO PAYS FOR THE RESEARCH?

Specimens will be taken at no cost to you or your insurance company. This study has been designed so that no additional study doctor visits should be required to participate. However, please note that additional doctor visits to follow your disease status, as required by a doctor, are a part of your regular treatment and the associated costs will be your responsibility.

*(MANDATORY TEXT– to be completed by Investigator) [Include possible Conflicts of Interest either financial (e.g., Johnson & Johnson stock) or Institutional Affiliation(s).)*

## WILL I BE PAID?

You will not be paid for your participation in this research study. You will not be paid for any use of your samples, results, or inventions made from them. If you take part, you are providing your samples for use by the sponsor. The sponsor (and research partners, where applicable) plan(s) to own the use of the results from the conduct of this research study. It will not cost you any money to take part in this research.

## WHO COULD PROFIT OR FINANCIALLY BENEFIT FROM THE STUDY RESULTS?

The collection of your blood and/or the results of the research may lead to discoveries that could economically benefit the sponsor, study physician and/or research center(s) engaged in these activities. There are no plans to compensate you for any products developed from this research. By signing this consent form, you authorize the use of your samples for the purposes of this research study only.

## WILL I GET MY TEST RESULTS FROM THIS RESEARCH?

The CTC assays performed as part of this study are investigational and for research use only, and we are unsure of the meaning of the results. These results will not be used to make any decisions regarding your treatment. Neither you nor your caregiver will be given the results of the CTC assays performed as part of this research study nor will they be included in your medical records. However, if the testing of the blood sample taken prior to the start of your new endocrine therapy is unsuccessful, then your study doctor will be notified and your direct participation in the study will be completed immediately.

The results from genetic tests that will be performed, if you consent to participate in this optional blood draw and/or tissue archival, are for research purposes only and will not be shared with you or your caregiver.

## CAN I CHANGE MY MIND?

Your participation is voluntary. You can agree to be in the study now and change your mind later. If you leave the study before it is finished, there will be no penalty to you, you will not lose any benefits to which you may otherwise be entitled, and it will not affect your access to the care, medicine, and equipment you would otherwise be getting. If you withdraw your consent for this research, you would need to tell your study doctor. Your study doctor will then tell the sponsor to destroy your stored samples (i.e. CTC and tissue).

When you withdraw your permission, no new health information which might identify you will be gathered after that date. If you do decide to withdraw your consent, you agree not to limit the sponsor's use of your research data that has already been collected.

The study doctor may stop you from taking part in this study at any time if he/she believes it is in your best interest, if you do not follow the study rules, if you become ineligible to participate, if your condition changes and you need treatment that is not allowed while you are taking part in the study, or if the study is suspended or canceled for any reason.

If you withdraw your consent after the study is over, it is possible that your study doctor may have already discarded the medical records that link your name to your study number. This is because your study doctor will only keep your personal medical records and a list that links each patient's name to his or her study number for up to ten (10) years. In this case, your samples would no longer be linked to you. It would not be possible to find your samples to destroy them.

## WHAT ARE MY ALTERNATIVES?

This is not a treatment study. Your alternative is not to participate in this study. Your other choices may include:

- Getting treatment or care for your cancer without being in a study
- Taking part in another study

Talk to your doctor about your choices before you decide if you will take part in this study.

## WHAT HAPPENS TO THE INFORMATION COLLECTED ABOUT ME?

Federal regulations give you certain rights related to your health information. These include the right to know who will be able to get the information and why they may be able to get it. The study doctor must get your authorization (permission) to use or give out any health information that might identify you. Study records that identify you will be kept as required by law. Federal Privacy Regulations provide safeguards for privacy, security and authorized access. Except when required by law, you will not be identified by name, social security number, address, telephone number, or any other direct personal identifier in study records disclosed outside of the study doctor. Your authorization for the use and disclosing of your health information collected during the conduct of this research study does not have an expiration date.

If you choose to be in this study, the study doctor will obtain personal information about you. This may include information that might identify you. The study doctor may also obtain information about your health including:

- Medical and research records
- Records about your study visits
- Records of physical exams
- Treatment records
- Laboratory, imaging, and other test results

Your treating institutional policies require that private information about you be protected. This is especially true of your personal health information. The investigator of the study will maintain all records confidentially in a protected, locked cabinet or storage unit and (in the case of the data) in coded, password protected computerized database. Therefore, there is a low risk of loss or breach of confidentiality; however, the investigators and the sponsor of the study will take measures to protect your information including limiting access to your identifiable information only to those individuals necessary to fulfill the requirements of the study.

Information about you and your health that might identify you may be given to others by the study doctor and study staff to carry out the research study. Your study doctor will keep your personal medical records and a list that links this to your code number for up to 10 years. Regulatory authorities, members of the ethics committee/institutional review board, employees at the study site and representatives of the sponsor will have access to this list and be able to compare and check the study information collected about you with information in your medical records. By signing this form, you are allowing direct access to your medical records by these listed groups. As far as the law allows, your medical records will not be made public.

By signing this consent form, you are giving permission to use and give out the health information for the purposes of this research. If you refuse to give permission, you will not be able to be in this research.

Your information will be given to the sponsor of this research. “Sponsor” includes any persons or companies that are working for or with the sponsor, or are owned by the sponsor. The sponsor will use the information collected about you in the study for the purpose of conducting the research. The sponsor will analyze and evaluate the results of the study for medical, statistical, and regulatory purposes related to the research.

Information about you and your health which might identify you may also be given to:

- The U.S. Food and Drug Administration (FDA);
- Department of Health and Human Services (DHHS) agencies;
- Governmental agencies in other countries; and
- The Investigational Review Board (IRB)

Absolute confidentiality cannot be guaranteed because of the need to give information to these parties. Note that once your information has been shared with others, it may no longer be protected by the privacy regulations of the federal Health Insurance Portability and Accountability Act of 1996 (HIPAA).

The information may be given to the FDA, DHHS agencies and governmental agencies in other countries so the sponsor can receive marketing approval for new products resulting from this research. The information may also be used to meet the reporting requirements of these governmental agencies.

The information may be reviewed by the IRB. The IRB is a group of people who perform independent review of research as required by regulations.

Your information may also be shared with research partners for scientific research purposes. Before sharing with research partners, your information will be labeled with a code number that is different from your study code number. Your information will not contain any personal identifiers. Your information will not be sold, loaned or given to any other independent groups for their own use. Research partners working with the sponsor are not allowed to share your information with anyone who is not authorized by the sponsor. The sponsor will control what is done with your information.

The results of this research may be published in scientific journals or presented at medical meetings, but your identity will not be disclosed. A description of this clinical trial will be available on www.ClinicalTrials.gov, as required by US law. This website will not include information that can identify you. At most, the website will include a summary of the results. You can search this website any time.

You have the right to review and copy your health information. You can arrange with your study doctor to see the information collected about you, and you can ask for any mistakes to be corrected. The sponsor may postpone your access to your information if it would interfere with the study itself. If you decide to leave the study at any time, the sponsor may still use your information collected up to that point, if the law allows.

## WHAT HAPPENS TO THE SAMPLES COLLECTED FROM ME?

Your blood and tissue samples collected under this study will only be used for the research purposes and stored as described in this form.

To protect your privacy, your samples will be labeled with a coded Sample ID number. The scientists doing the research will not know your identity.

Your samples may be sent to other members of the Johnson & Johnson group of companies, to contractors or other outside institution working for them and to regulatory authorities.

Your samples may also be shared with research partners for scientific research purposes. Before sharing with research partners, your samples will be labeled with a code number that is different from your study code number. Your samples will not contain any personal identifiers. Your samples will not be sold, loaned or given to any other independent groups for their own use. Research partners working with the sponsor are not allowed to share samples with anyone who is not authorized by the sponsor. The sponsor will control what is done with your samples.

Some or all of your research study samples may also be kept and used for up to 10 years. This will allow for the scientific research described above to be done in the future as new discoveries are made. The sponsor will ensure that your samples are stored securely. Your samples will

be destroyed no later than 10 years after the completion of the original study. You will not be informed when they are destroyed.

## WHY IS THIS OPTIONAL PART BEING DONE?

Researchers are trying to learn more about cancer. Much of this research is done using samples from your tissue, blood, urine, or other fluids. Through these studies, researchers hope to find new ways to prevent, detect, treat, or cure health problems. Some of these studies may be about genes. Genes carry information about features that are found in you and in people who are related to you. Researchers are interested in the way that genes affect how your body responds to treatment.

## WHAT ARE THE BENEFITS OF TAKING PART IN THIS OPTIONAL RESEARCH?

There is no direct or medical benefit to you as a result of this optional blood draw. By taking part however, you may contribute to science and medicine and help define the future clinical usefulness of this laboratory test.

## WHAT ARE THE RISKS OF GENETIC RESEARCH?

The physical risks of most genetic tests are small.

However, these concerns pertain to studies of DNA/genes in which the information would be returned to you or your doctor. In this case, the studies done in this set of investigations are entirely investigational, which means we do not know what they mean or even if the results we get are reliable. Therefore, we will not release the results to you or your doctor, or to anyone else, since we are not sure of their meaning. If you wish to have genetic testing to see if you are at risk for some disease, you should speak with your doctor or caregiver.

**Genetic Information Nondiscrimination Act (GINA)** –

The federal Genetic Information Nondiscrimination Act (GINA) generally makes it illegal for health insurance companies, group health plans, and most employers to discriminate against you based on your genetic information. This law does not protect you against genetic discrimination by companies that sell life insurance, disability insurance, or long-term care insurance. Under this law:

- Health insurance companies and group health plans may not request your genetic information that we obtain from this research
- Health insurance companies and group health plans may not use your genetic information when making decisions regarding your eligibility or premiums
- Employers with 15 or more employees may not use your genetic information that we obtain from this research when making a decision to hire, promote, or fire you or when setting the terms of your employment

GINA does not apply to the following groups, however these groups have policies in place that provide similar protections against discrimination:

- Members of the US Military receiving care through Tricare
- Veterans receiving care through the Veteran's Administration (VA)
- The Indian Health Service
- Federal employees receiving care through the Federal Employees Health Benefits Plans

## WHO DO I CONTACT FOR INFORMATION?

**If you have any questions or problems during this study, or if you think that you may have experienced a research-related injury, please contact:**

**[Insert appropriate study site personnel name, phone number, and title]**

**If you have any questions about your rights as a research patient, please contact the Institutional Review Board listed below:**

**[Insert IRB or IEC name and phone number]**

Do not sign this consent form unless you have had a chance to ask questions and have received satisfactory answers to all of your questions.

If you agree to participate in this study, you will be given a signed and dated copy of this consent form.

## SUMMARY STATEMENT

You have read and understood the information which has been stated above and have received satisfactory answers to all of the questions which you have asked and you willingly sign this consent form. It is understood that your decision to participate in this study or to withdraw from this study will not influence the availability of future medical care and will involve no penalty or loss of benefits to which you are otherwise entitled. You are free to withdraw from this study at any time, and if you decide to do so, you agree to inform the study doctor immediately. You also understand that the physician in charge of the study can remove you from this study without your consent for any reason.

***If you consent, please read and then sign below.***

This consent form contains important information. It will help you decide if you want to take part in the optional research. If you still have questions, please ask the study doctor or one of the study staff, before signing this form.

**Agreement to take part in the research study:**

- I have read in a language that I understand well, the above information.
- The content and meaning of this information has been explained to me.
- All my questions about the research study and possible risks have been answered to my satisfaction. I understand that if I have more questions or concerns about the study or my participation as a research subject at a later date, I may contact my study doctor or the IRB as indicated above.
- Based on this information, I hereby voluntarily consent and offer to take part in this study and authorize the use and disclosure of my medical information.

| | | |
|---|---|---|
| Date (MM/DD/YY) | Print Subject Name | Subject Signature |
| Date (MM/DD/YY) | Name of Person conducting the Informed Consent discussion | Signature of Person conducting the Informed Consent discussion |

I understand and I agree to give my permission to collect part of my primary and/or metastatic cancer tissue specimens as part of the study :

☐ YES ☐ NO Subject Initials: __________

I understand and I agree to give my permission to collect the optional blood draw for cfDNA analysis:

☐ YES ☐ NO Subject Initials: __________

I understand and I agree to give my permission to store the CTCs found in my blood for future testing.

☐ YES ☐ NO Subject Initials: __________

**Principal Investigator (or Designee):**

I have given this research subject (or his/her legally authorized representative, if applicable) information about this study that I believe is accurate and complete. The subject has indicated that he or she understands the nature of the study and the risks and benefits of participating.

___________ ______________________________ ________________________________
Date (MM/DD/YY) Print Investigator Name Investigator Signature

Copy of consent form given to subject on (date) ______________ by (initials) ______________

# Research Subject Information and Consent Form
# Addendum- Additional Testing

You have previously participated in the COMETI study in which you gave consent for investigators to collect blood from you so that they could study cancer cells that might be in your blood. These investigators would now like to take these cells that they have stored, with your permission, and study if they can perform genetic analysis using novel technology. This form gives you important information about this addition to the study. It describes the purpose of this addition to the study, and the risks and possible benefits of participating in this part of the study.

**Study Sponsor:** **Janssen Diagnostics, LLC**

*(Janssen Diagnostics, LLC is part a company of Johnson & Johnson)*

---

**Protocol Number:** COMETI-P2-2012.0

**Protocol Title:** COMETI Phase 2: Characterization of Circulating Tumor Cells from Subjects with Metastatic Breast Cancer Using the CTC-Endocrine Therapy Index

**Simplified Title:** COMETI Phase 2

**Study Doctor:** [Insert Principal Investigator name]

**Study Staff:** [Insert Sub-Investigator name(s)]

**Study Site(s):** [Insert Site Name]
[Insert Address (Line 1)]
[Insert Address (Line 2)]
[Insert Address (Line 3), if applicable]
[Insert City, State Zip Code]

**24 HR Telephone #:** [Insert phone number]

---

**Please read this document carefully**

You are being invited to take part in a clinical trial, a type of research study. Taking part in this study is voluntary. Before you decide, you should know why the research is being done and what it involves. Please read this consent form carefully, as it may contain words that you do not understand. Ask the study doctor (or his or her staff) to explain any words or information that you do not clearly understand as well as any other questions you may have. You may take an unsigned copy of this form home with you to read again. Take your time to think and talk about it with your family and friends before making your decision.

## STUDY PURPOSE

The purpose of the experimental study that you already have participated in was to determine the feasibility of investigating CTC and the expression of several biomarkers on/in the tumor cells that can be detected in blood of patients with metastatic breast cancer. We speculate that the

combination of CTC numbers and expression levels of biomarkers (including ER together with other markers called Bcl-2, HER2, and Ki67) on/in the CTC will tell us if a patient who has ER positive breast cancer is unlikely to benefit from endocrine therapy and would be better treated with chemotherapy.

We now wish to look at the molecular make-up of tumor cells in your blood. This make-up includes genetics, proteomics and other biomarkers. We believe that advances in the treatment and prevention of cancer will be based on better understanding of specific molecular and genetic alterations typical of individual tumors.

The consent form you signed previously did not give us permission to do very detailed analysis of the genes of your cancer, or to compare them with your normal genes. Technology has advanced very quickly, and now we would like to ask your permission to use the cancer cells we have already collected from your blood to study the cancer genes and compare them to your normal genes.
To be clear, we are not asking for more blood from you. Rather, we are simply asking for permission to study the already collected cells from your blood, which with your permission we had already stored in the laboratory.

We hope to identify traits that will distinguish which tumors can be treated most successfully by different treatment options. In order to study the genetics of these cancers, we will study the cells that we collected from your blood and stored with your consent when you agreed to participate in this study. We plan to purify the DNA from these cells and determine if there are any changes in DNA (called mutation, deletion, amplification, or translocation) compared to your normal DNA.

In addition, researchers have found that DNA from a patient's cancer can be found in the patient's blood. This is called circulating cell free DNA (cfDNA). CfDNA from your blood will also be analyzed because both CTC-DNA and cfDNA might be useful to understand your cancer status. From now on, we will also study what molecules the cancer might be making. In particular, we want to extract the cfDNA from the blood taken from 2 different types of tubes and compare the results.

In the future, knowing such information might help doctors better choose the right therapy for patients with cancer. By targeting treatment approaches in this way, we hope to improve outcomes while minimizing harmful effects of treatment.

## WHAT WILL HAPPEN TO ME IF I CHOOSE TO TAKE PART ?

You have previously given consent for investigators to collect blood from you so that they could study cancer cells that might be in your blood. After the research studies were performed, the sample cartridges containing cancer cells from your blood were stored, with your permission, for 10 years for potential future research such as development of new markers on the cancer cells. We would now like to take these cells that have been stored, with your permission, and study if they can perform genetic analysis using novel technology.

We will attempt to perform a thorough genetic analysis of CTC, including DNA and RNA. We will compare the DNA of CTC and your normal cells, including DNA and RNA. We will perform very

detailed genetic testing on them that may guide treatment in the future. Samples may also be stored and used for future studies of gene associated with cancer. Specimens are distributed only to members of our research team or to other investigators who have secured IRB (ethics board) approval. Specimens are distributed to outside investigators without any identifying information that would link a specimen to an individual person. For cfDNA analysis, we are planning to draw an additional tube of blood at each time point. If you are still actively participating in the study, we will now draw an additional tube of blood for this analysis.

Some studies will use the biological specimens in genetic analysis looking for molecular markers, genes, and/or proteins that may be used to predict response of specific tumors to specific treatment options. However, these are entirely investigational (experimental), and therefore neither you nor your doctor will be told the results of any testing on your specimens, since we do not know if any of these results are clinically important.

## WHAT ARE THE RISKS OF GENETIC RESEARCH?

In this study, blood samples drawn from you have been already examined for the existence of CTC. We are now asking for permission to perform genetic analysis on these previously collected CTC if possible.

The physical risks of most genetic tests are small.

However, these concerns pertain to studies of DNA/genes in which the information would be returned to you or your doctor. In this case, the studies done in this set of investigations are entirely investigational, which means we do not know what they mean or even if the results we get are reliable. Therefore, we will not release the results to you or your doctor, or to anyone else, since we are not sure of their meaning. If you wish to have genetic testing to see if you are at risk for some disease, you should speak with your doctor or caregiver.

For more information about any risks, ask your study doctor.

Genetic Information Nondiscrimination Act (GINA) – If the research involves genetic analysis of biological samples, insert the following two paragraphs:

The federal Genetic Information Nondiscrimination Act (GINA) generally makes it illegal for health insurance companies, group health plans, and most employers to discriminate against you based on your genetic information. This law does not protect you against genetic discrimination by companies that sell life insurance, disability insurance, or long-term care insurance. Under this law:

- Health insurance companies and group health plans may not request your genetic information that we obtain from this research
- Health insurance companies and group health plans may not use your genetic information when making decisions regarding your eligibility or premiums

- Employers with 15 or more employees may not use your genetic information that we obtain from this research when making a decision to hire, promote, or fire you or when setting the terms of your employment

GINA does not apply to the following groups, however these groups have policies in place that provide similar protections against discrimination:

- Members of the US Military receiving care through Tricare
- Veterans receiving care through the Veteran's Administration (VA)
- The Indian Health Service
- Federal employees receiving care through the Federal Employees Health Benefits Plans

These markers can then be used to develop tailored treatment regimens that improve outcome while minimizing side effects of treatment. Concerning CTC, it is unknown if genetic information of CTC reflects the characteristics of your cancer. Therefore these data will not be told to you.

## WHO DO I CONTACT FOR INFORMATION?

**If you have any questions or problems during this study, or if you think that you may have experienced a research-related injury, please contact:**

**[Insert appropriate study site personnel name, phone number, and title]**

**If you have any questions about your rights as a research patient, please contact the Institutional Review Board listed below:**

**[Insert IRB or IEC name and phone number]**

Do not sign this consent form unless you have had a chance to ask questions and have received satisfactory answers to all of your questions.

If you agree to participate in this study, you will be given a signed and dated copy of this consent form.

## SUMMARY STATEMENT

You have read and understood the information which has been stated above and have received satisfactory answers to all of the questions which you have asked and you willingly sign this consent form. It is understood that your decision to participate in this study or to withdraw from this study will not influence the availability of future medical care and will involve no penalty or loss of benefits to which you are otherwise entitled. You are free to withdraw from this study at any time, and if you decide to do so, you agree to inform the study doctor immediately. You also understand that the physician in charge of the study can remove you from this study without your consent for any reason.

***If you consent, please read and then sign below.***

This consent form contains important information. It will help you decide if you want to take part in the optional research. If you still have questions, please ask the study doctor or one of the study staff, before signing this form.

I understand and I agree to give my permission to perform this additional research on the stored specimens as part of the study:

☐ YES ☐ NO Subject Initials: __________

| ____________ | ______________________________ | ______________________________ |
|---|---|---|
| Date (MM/DD/YY) | Print Subject Name | Subject Signature |
| Date (MM/DD/YY) | Name of Person conducting the Informed Consent discussion | Signature of Person conducting the Informed Consent discussion |

**Principal Investigator (or Designee):**

I have given this research subject (or his/her legally authorized representative, if applicable) information about this study that I believe is accurate and complete. The subject has indicated that he or she understands the nature of the study and the risks and benefits of participating.

| ____________ | ______________________________ | ______________________________ |
|---|---|---|
| Date (MM/DD/YY) | Print Investigator Name | Investigator Signature |

Copy of consent form given to subject on (date) ______________ by (initials) _____________

# APPENDIX B

## Example CRFs & Study Logs

# COMETI-P2-2012.0

**COMETI Phase 2: Characterization of Circulating Tumor Cells from Subjects with Metastatic Breast Cancer Using the CTC-Endocrine Therapy Index**

## Case Report Forms for:

**Site #**    **Subject #**

☐☐ – ☐☐☐

Ver. 21-SEP-2012 CRF Cover Page CP01
DISTRIBUTION: White – Veridex, LLC; Yellow – Investigator

*PRODUCTS ARE FOR RESEARCH USE ONLY AND THEIR PERFORMANCE CHARACTERISTICS ON CLINICAL SAMPLES HAVE NOT BEEN DETERMINED*

# PROTOCOL COMETI-P2-2012.0

**"Characterization of CTC in Metastatic Breast Cancer Subjects using CTC-Endocrine Therapy Index"**

**Site #:** ☐☐ **Subject #:** ☐☐☐

## ELIGIBILITY FORM

| INCLUSION CHECKLIST | YES | NO |
|---|---|---|
| 1. Female ≥ 18 Years Old | ☐ | ☐ |
| 2. Metastatic Breast Cancer (non-local metastases required that is either measurable or non-measurable according to RECIST v1.1 criteria) | ☐ | ☐ |
| 3. At least one non-irradiated distant site of metastasis | ☐ | ☐ |
| 4. ECOG performance status 0 – 2 | ☐ | ☐ |
| 5. ER positive / HER2 negative breast cancer | ☐ | ☐ |
| 6. Currently progressive metastatic disease according to RECIST v1.1 criteria | ☐ | ☐ |
| 7. Progressed on at least one previous line of ET for their metastatic disease OR have evidence of progression during or within 12 months of completing adjuvant ET | ☐ | ☐ |
| 8. About to start a new line of ET for their metastatic disease | ☐ | ☐ |
| 9. Disease evaluation (consistent whole body anatomic and/or bone scintigraphic imaging) must be anticipated prior to initiation of therapy and subsequently every 3 months | ☐ | ☐ |
| 10. Subject willing/able to provide ~40mL of blood at baseline (prior to initiation of new ET) and subsequently 1, 2, 3, and 12 months after the initiation of therapy, or at the time of disease progression, whichever occurs first | ☐ | ☐ |
| 11. Subject consents and approves of material (i.e. blood) collection & transfer | ☐ | ☐ |
| **Inclusion Criteria (any "NO" response excludes patient from study)** | | |

| EXCLUSION CHECKLIST | YES | NO |
|---|---|---|
| 1. Male Subject | ☐ | ☐ |
| 2. Subject concurrently participating in a therapeutic clinical trial addressing ET plus or versus an additional investigational treatment | ☐ | ☐ |
| 3. Subject has local regional recurrence only OR brain only metastasis (subjects with CNS metastases are eligible if they also have progression of metastatic disease elsewhere and the CNS metastases are proven to be stable for 3 months or more after completion of CNS therapy (surgery or radiation)) | ☐ | ☐ |
| 4. Subject progressing on current fulvestrant therapy | ☐ | ☐ |
| 5. Subject will be taking any unapproved (i.e. not cleared/approved by the FDA) anti-neoplastic therapy, other than everolimus, concurrently | ☐ | ☐ |
| 6. Subject has concomitant or previous other malignancies within the last 5 years, with the exception of adequately treated basal or squamous cell carcinoma of the skin or carcinoma in situ of the cervix | ☐ | ☐ |
| 7. Unable to provide informed consent or high risk that subject may not comply with protocol requirements (i.e. due to health and/or participation in other research studies) | ☐ | ☐ |
| **Exclusion Criteria (any "YES" response excludes patient from study)** | | |

**Subject meets all eligibility criteria:** ☐Yes ☐No* (*Protocol Deviation Form and Sponsor Approval required)

**Date of signed informed consent:** ___ ___ - ___ ___ ___ - ___ ___ ___ ___
DD MMM YYYY

**Ver. 21-SEP-2012** **Eligibility Form** **E01**
**DISTRIBUTION: White – Veridex, LLC; Yellow – Investigator**

*PRODUCTS ARE FOR RESEARCH USE ONLY AND THEIR PERFORMANCE CHARACTERISTICS ON CLINICAL SAMPLES HAVE NOT BEEN DETERMINED*

# PROTOCOL COMETI-P2-2012.0

"Characterization of CTC in Metastatic Breast Cancer Subjects using CTC-Endocrine Therapy Index"

**Site #:** ☐☐ **Subject #:** ☐☐☐

## BASELINE FORM

### GENERAL PATIENT INFORMATION

**Date of Birth:** _____ - _______ - _______ (DD MMM YYYY)

**Height:** _______ ' _______ " (Feet Inches)

**Weight:** ___________ (Pounds)

**Race / Ethnicity:**
☐ White ☐ Black or African American ☐ Asian
☐ Hispanic or Latino ☐ American Indian or Alaska Native
☐ Native Hawaiian or Other Pacific Islander ☐ Unknown
☐ Other: ________________________________________

**Menopausal Status:** ☐ PRE ☐ PERI ☐ POST **ECOG Performance Status:** ☐ 0 ☐ 1 ☐ 2

**Family History of Breast Cancer:** ☐ Unknown ☐ No ☐ Yes Relationship: ____________________

### BREAST CANCER INFORMATION

**Date of Primary Diagnosis:** _____ - _______ - _______ (DD MMM YYYY)

**Stage of Disease at Primary Diagnosis** (based on AJCC Staging)
Primary Tumor (T) T ______
Regional Lymph Nodes (N) N ______
Distant Metastasis (M) M______

**Grade (at surgery or biopsy):**
☐ GX (Grade not assessed)
☐ G1 (Well-differentiated)
☐ G2 (Moderately-differentiated)
☐ G3 (Poorly-differentiated)

**Primary Tumor Histology (check all that apply):** ☐ Invasive Carcinoma ☐ Inflammatory
☐ Ductal Carcinoma *in-situ* (DCIS) ☐ Lobular Carcinoma *in-situ* (LCIS) ☐ NOS (Not Otherwise Specified)
☐ Other (Specify): ________________________________________

**Oncotype DX™ Result:** Recurrence Score = _________ ☐ Unknown/Not Done

**MammaPrint® Result:** ☐ Low Risk ☐ High Risk ☐ Unknown/Not Done

**Primary Tumor Phenotype:**

| | *Unknown* | *Scoring Method* | *% Cells +* | *Average Intensity of Staining* | *Interpretation* |
|---|---|---|---|---|---|
| ER: | ☐ | __________ | ______ | ☐ None ☐ Weak ☐ Intermediate ☐ Strong | ☐ - ☐ + |
| PR: | ☐ | __________ | ______ | ☐ None ☐ Weak ☐ Intermediate ☐ Strong | ☐ - ☐ + |
| Ki67: | ☐ | __________ | ______ | ☐ None ☐ Weak ☐ Intermediate ☐ Strong | ☐ - ☐ + |
| Bcl2: | ☐ | __________ | ______ | ☐ None ☐ Weak ☐ Intermediate ☐ Strong | ☐ - ☐ + |

Her2/neu: IHC: ☐ Negative (0) ☐ 1+ ☐ 2+ ☐ 3+ ☐ Unknown / Not Done
FISH / SISH / CISH: ☐ Positive ☐ Negative ☐ Equivocal Average Ratio: ________ ☐ Unknown / Not Done

**Metastatic Tumor Phenotype:**

| | *Unknown* | *Scoring Method* | *% Cells +* | *Average Intensity of Staining* | *Interpretation* |
|---|---|---|---|---|---|
| ER: | ☐ | __________ | ______ | ☐ None ☐ Weak ☐ Intermediate ☐ Strong | ☐ - ☐ + |
| PR: | ☐ | __________ | ______ | ☐ None ☐ Weak ☐ Intermediate ☐ Strong | ☐ - ☐ + |
| Ki67: | ☐ | __________ | ______ | ☐ None ☐ Weak ☐ Intermediate ☐ Strong | ☐ - ☐ + |
| Bcl2: | ☐ | __________ | ______ | ☐ None ☐ Weak ☐ Intermediate ☐ Strong | ☐ - ☐ + |

Her2/neu: IHC: ☐ Negative (0) ☐ 1+ ☐ 2+ ☐ 3+ ☐ Unknown / Not Done
FISH / SISH / CISH: ☐ Positive ☐ Negative ☐ Equivocal Average Ratio: ________ ☐ Unknown / Not Done

**Date of Metastatic Diagnosis:** _____ - _______ - _______ (DD MMM YYYY)

**Current Sites of Metastasis (Check all that apply):**
☐ Abdomen ☐ Adrenal ☐ Ascites ☐ Bone ☐ Brain
☐ Chest Wall ☐ Kidney ☐ Liver ☐ Lung ☐ Lymph Node(s)
☐ Other: ________________________________________

Ver. 21-SEP-2012 Baseline Form BL01
DISTRIBUTION: White – Veridex, LLC; Yellow – Investigator

# PROTOCOL COMETI-P2-2012.0

"Characterization of CTC in Metastatic Breast Cancer Patients using CTC-Endocrine Therapy Index"

**Site #:** ☐☐ **Subject #:** ☐☐☐

## PRIOR BREAST CANCER THERAPY

Instructions: List all treatments for breast cancer (neoadjuvant, adjuvant and metastatic) since the date of the primary diagnosis. Use additional pages if necessary, using A, B, C, etc. after page number to indicate multiple pages.

### PRIOR SURGICAL INTERVENTION FOR PRIMARY BREAST CANCER ☐ NONE

| Type of Surgery / Site (i.e. Wedge resection, left breast; mastectomy, right breast) | Date (DD-MMM-YYYY) |
|---|---|
| | |
| | |

### PRIOR RADIATION THERAPY ☐ NONE

| Site(s) | Reason? | Start Date (DD-MMM-YYYY) | Stop Date (DD-MMM-YYYY) |
|---|---|---|---|
| ☐ Breast ☐ Lymph Node(s) ☐ Local ☐ Distant Mets | ☐ Palliative ☐ Curative | | |
| ☐ Breast ☐ Lymph Node(s) ☐ Local ☐ Distant Mets | ☐ Palliative ☐ Curative | | |

### ENDOCRINE (HORMONE) THERAPY

| Drug Name / Dosage / Schedule | Dosing / Schedule | Start Date (DD-MMM-YYYY) | Stop Date (DD-MMM-YYYY) | Check if Ongoing |
|---|---|---|---|---|
| ☐ Oophorectomy ☐ Tamoxifen ☐ Toremifene ☐ Letrozole ☐ Anastrozole ☐ Exemestane ☐ Fulvestrant ☐ Megace ☐ LHRH Analog ☐ Other (Specify): ____________ | | | | ☐ |
| ☐ Oophorectomy ☐ Tamoxifen ☐ Toremifene ☐ Letrozole ☐ Anastrozole ☐ Exemestane ☐ Fulvestrant ☐ Megace ☐ LHRH Analog ☐ Other (Specify): ____________ | | | | ☐ |
| ☐ Oophorectomy ☐ Tamoxifen ☐ Toremifene ☐ Letrozole ☐ Anastrozole ☐ Exemestane ☐ Fulvestrant ☐ Megace ☐ LHRH Analog ☐ Other (Specify): ____________ | | | | ☐ |

### PRIOR CHEMOTHERAPY AND/OR HER-2/neu TARGETED THERAPY ☐ NONE

| Drug Name | Start Date (DD-MMM-YYYY) | Stop Date (DD-MMM-YYYY) | Check if Ongoing |
|---|---|---|---|
| | | | ☐ |
| | | | ☐ |

### PRIOR OTHER THERAPIES (i.e. IMMUNOTHERAPY, EXPERIMENTAL, BISPOHOSPHONATES, ETC.) ☐ NONE

| Drug Name | Start Date (DD-MMM-YYYY) | Stop Date (DD-MMM-YYYY) | Check if Ongoing |
|---|---|---|---|
| | | | ☐ |
| | | | ☐ |
| | | | ☐ |

Ver. 21-SEP-2012 Prior Therapy Form PT01 ____

DISTRIBUTION: White – Veridex, LLC; Yellow – Investigator

*PRODUCTS ARE FOR RESEARCH USE ONLY AND THEIR PERFORMANCE CHARACTERISTICS ON CLINICAL SAMPLES HAVE NOT BEEN DETERMINED*

# PROTOCOL COMETI-P2-2012.0

"Characterization of CTC in Metastatic Breast Cancer Patients using CTC-Endocrine Therapy Index"

**Site #:** ☐☐ **Subject #:** ☐☐☐

## ON-STUDY THERAPY

Instructions: List all treatments for metastatic breast cancer administered during the subject's participation in the study. Use additional pages if necessary, using A, B, C, etc. after page number to indicate multiple pages.

### ENDOCRINE (HORMONE) THERAPY

| Drug Name / Dosage / Schedule | Dosing / Schedule | Start Date (DD-MMM-YYYY) | Stop Date (DD-MMM-YYYY) | Check if Ongoing from Previous |
|---|---|---|---|---|
| ☐ Oophorectomy ☐ Tamoxifen ☐ Toremifene<br>☐ Letrozole ☐ Anastrozole ☐ Exemestane<br>☐ Fulvestrant ☐ Megace ☐ LHRH Analog<br>☐ Other (Specify): ____________ | | | | ☐ |
| ☐ Oophorectomy ☐ Tamoxifen ☐ Toremifene<br>☐ Letrozole ☐ Anastrozole ☐ Exemestane<br>☐ Fulvestrant ☐ Megace ☐ LHRH Analog<br>☐ Other (Specify): ____________ | | | | ☐ |
| ☐ Oophorectomy ☐ Tamoxifen ☐ Toremifene<br>☐ Letrozole ☐ Anastrozole ☐ Exemestane<br>☐ Fulvestrant ☐ Megace ☐ LHRH Analog<br>☐ Other (Specify): ____________ | | | | ☐ |

### PALLIATIVE RADIATION THERAPY FOR PRE-EXISTING SYMPTOMS/CONDITIONS ☐ NONE

| Site(s) | Start Date (DD-MMM-YYYY) | Stop Date (DD-MMM-YYYY) |
|---|---|---|
| ☐ Breast ☐ Lymph Node(s) ☐ Local ☐ Bone/Spine ☐ Other Distant Mets | | |
| ☐ Breast ☐ Lymph Node(s) ☐ Local ☐ Bone/Spine ☐ Other Distant Mets | | |

### OTHER SUPPORTIVE CARE THERAPIES (I.E. BISPHOSPHONATES, RANKL INHIBITORS, ETC.) ☐ NONE

| Therapy Name | Start Date (DD-MMM-YYYY) | Stop Date (DD-MMM-YYYY) | Check if Ongoing from Previous |
|---|---|---|---|
| | | | ☐ |
| | | | ☐ |
| | | | ☐ |
| | | | ☐ |

### COMMENTS ☐ NONE

Ver. 21-SEP-2012 On-Study Therapy Form TX01 ____
DISTRIBUTION: White – Veridex, LLC; Yellow – Investigator

*PRODUCTS ARE FOR RESEARCH USE ONLY AND THEIR PERFORMANCE CHARACTERISTICS ON CLINICAL SAMPLES HAVE NOT BEEN DETERMINED*

# PROTOCOL COMETI-P2-2012.0

"Characterization of CTC in Metastatic Breast Cancer Subjects using CTC-Endocrine Therapy Index"

**Site #:** ☐☐ **Subject #:** ☐☐☐

## PHYSICIAN NOTES

Instructions: Please record overall physician impression of clinical status of the subject at each visit during their participation in the study (i.e. cancer related signs and symptoms and changes in performance status, if captured).

| Visit | Date of Visit (DD-MMM-YYYY) | Notes / Remarks | ECOG Status |
|---|---|---|---|
| Baseline | | | ☐* |
| Month 1 | | | ☐* |
| Month 2 | | | ☐* |
| Month 3 | | | ☐* |
| Month 6 | | | ☐* |
| Month 9 | | | ☐* |
| Month 12 or Off Study | | | ☐* |

*Unknown

## SERUM TUMOR MARKERS

Instructions: Please record results of serum tumor markers measured at each protocol required visit during the subject's participation in the study.

| Visit | Date of Visit (DD-MMM-YYYY) | CEA (ng/mL) | CA15-3 (U/mL) | CA27.29 (U/mL) |
|---|---|---|---|---|
| Baseline | | ☐** | ☐** | ☐** |
| Month 1 | | ☐** | ☐** | ☐** |
| Month 2 | | ☐** | ☐** | ☐** |
| Month 3 | | ☐** | ☐** | ☐** |
| Month 6 | | ☐** | ☐** | ☐** |
| Month 9 | | ☐** | ☐** | ☐** |
| Month 12 or Off Study | | ☐** | ☐** | ☐** |

**Not Evaluated

**Comments:** ☐ None

Ver. 21-SEP-2012 | Physician Notes & Serum Tumor Markers | PNST01

DISTRIBUTION: White – Veridex, LLC; Yellow – Investigator

# PROTOCOL COMETI-P2-2012.0

"Characterization of CTC in Metastatic Breast Cancer Subjects using CTC-Endocrine Therapy Index"

**Site #:** ☐☐ **Subject #:** ☐☐☐

## BASELINE TUMOR ASSESSMENT (RECIST v1.1)

**Instructions: Baseline imaging should be performed as close as possible to the start of treatment and never more than 3 weeks (21 days) prior to the start of treatment.** Target lesions should be selected on the basis of their size (lesions with longest diameter) and be representative of all involved organs, as well as their suitability for accurate repetitive measurements. Please refer to Section 9 of the protocol for definitions. Identify up to five measurable lesions total (maximum of two lesions per organ) to follow as target lesions. All other lesions (or sites of disease) not identified as target lesions, including pathological lymph nodes and all non-measurable lesions, should be recorded and followed as non-target lesions (up to a maximum of 10). Multiple non-target lesions within the same organ may be recorded as a single site (i.e. "multiple liver metastases").

### TARGET LESIONS ☐ NONE

| Lesion # | Description of Target Lesion(s) | Lesion Location [1] | Assessment Date (DD-MMM-YYYY) | Method of Assessment [2] | Longest Diameter (mm)[3] |
|---|---|---|---|---|---|
| T1 | | | | | |
| T2 | | | | | |
| T3 | | | | | |
| T4 | | | | | |
| T5 | | | | | |
| | | | | Sum of Longest Diameter – Target Lesions: | |

### NON-TARGET LESIONS ☐ NONE

| Lesion # | Description of Non-Target Lesion(s) | Lesion Location [1] | Assessment Date (DD-MMM-YYYY) | Method of Assessment [2] |
|---|---|---|---|---|
| NT1 | | | | |
| NT2 | | | | |
| NT3 | | | | |
| NT4 | | | | |
| NT5 | | | | |
| NT6 | | | | |
| NT7 | | | | |
| NT8 | | | | |
| NT9 | | | | |
| NT10 | | | | |

**Comments:** ☐ NONE

[1] **Lesion Locations:**
L1 – Bone
L2 – Brain
L3 – Breast
L4 – Liver
L5 – Lung
L6 – Lymph Node
L7 – Skin
L8 – Effusions / Ascites
L9 – GI (specify): ____________
L10 – GU (specify): ____________
L11 – Other (specify): ____________

[2] **Method of Assessment:**
A1 – Clinical Exam
A2 – Chest / Abdomen X-Ray
A3 – CT scan (non-spiral)
A4 – Spiral CT Scan
A5 – MRI scan
A6 – Bone scan
A7 – PET scan
A8 – Ultrasound
A9 – Other (specify): ____________

[3] **Lesion Diameter:** Record the longest diameter for each lesion. For lymph nodes, record the short axis measurement.

Ver. 21-SEP-2012 Baseline Tumor Assessment Form TM01

DISTRIBUTION: White – Veridex, LLC; Yellow – Investigator

*PRODUCTS ARE FOR RESEARCH USE ONLY AND THEIR PERFORMANCE CHARACTERISTICS ON CLINICAL SAMPLES HAVE NOT BEEN DETERMINED*

# PROTOCOL COMETI-P2-2012.0

"Characterization of CTC in Metastatic Breast Cancer Subjects using CTC-Endocrine Therapy Index"

Site #: ☐☐ Subject #: ☐☐☐

## FOLLOW-UP TUMOR ASSESSMENT (RECIST v1.1)

**Instructions:** The same assessment methods used at baseline must be used for all required follow-up disease assessments. Target lesions and non-target lesions followed must be the same ones selected at baseline. Record lesions in same order as listed on Baseline Tumor Assessment form (TM01). If a particular lesion (target and/or non-target) was not evaluated, indicate "NE" for the Assessment Date, Method of Assessment, and Longest Diameter (if T1-T5) or last column (if NT1-NT10). Objective tumor response must be determined from imaging time point to imaging time point using the RECIST v1.1 criteria.

**Imaging Period:** ☐ Month 3 ☐ Month 6 ☐ Month 9 ☐ Month 12 ☐ Off Study

### TARGET LESIONS ☐ NONE

| Lesion # | Description of Target Lesion(s) | Lesion Location [1] | Assessment Date (DD-MMM-YYYY) | Method of Assessment [2] | Longest Diameter (mm)[3] |
|---|---|---|---|---|---|
| T1 | | | | | |
| T2 | | | | | |
| T3 | | | | | |
| T4 | | | | | |
| T5 | | | | | |
| | | | | Sum of Longest Diameter – Target Lesions: | |

### EVALUATION OF TARGET LESIONS

☐ Complete Response (CR) ☐ Partial Response (PR) ☐ Stable Disease (SD) ☐ Progressive Disease (PD)
☐ Not All Lesions Evaluated Reason: ____________________

### NON-TARGET LESIONS ☐ NONE

| Lesion # | Description of Non-Target Lesion(s) | Lesion Location [1] | Assessment Date (DD-MMM-YYYY) | Method of Assessment [2] | Present (P), Absent (A) Unequivocal PD (PD) Not Evaluated (NE) |
|---|---|---|---|---|---|
| NT1 | | | | | |
| NT2 | | | | | |
| NT3 | | | | | |
| NT4 | | | | | |
| NT5 | | | | | |
| NT6 | | | | | |
| NT7 | | | | | |
| NT8 | | | | | |
| NT9 | | | | | |
| NT10 | | | | | |

### EVALUATION OF NON-TARGET LESIONS

☐ Complete Response (CR) ☐ Non-CR / Non-PD ☐ Progressive Disease (PD)
☐ Not All Lesions Evaluated Reason: ____________________

[1] **Lesion Locations:**
L1 – Bone
L2 – Brain
L3 – Breast
L4 – Liver
L5 – Lung
L6 – Lymph Node
L7 – Skin
L8 – Effusions / Ascites
L9 – GI (specify): ____________________
L10 – GU (specify): ____________________
L11 – Other (specify): ____________________

[2] **Method of Assessment:**
A1 – Clinical Exam
A2 – Chest / Abdomen X-Ray
A3 – CT scan (non-spiral)
A4 – Spiral CT Scan
A5 – MRI scan
A6 – Bone scan
A7 – PET scan
A8 – Ultrasound
A9 – Other (specify): ____________________

[3] **Lesion Diameter:** Record the longest diameter for each lesion. For lymph nodes, record the short axis measurement.

Ver. 21-SEP-2012 Follow-Up Tumor Assessment Form (FT01) Page 1 of 2
DISTRIBUTION: White – Veridex, LLC; Yellow – Investigator

*PRODUCTS ARE FOR RESEARCH USE ONLY AND THEIR PERFORMANCE CHARACTERISTICS ON CLINICAL SAMPLES HAVE NOT BEEN DETERMINED*

# PROTOCOL COMETI-P2-2012.0

"Characterization of CTC in Metastatic Breast Cancer Subjects using CTC-Endocrine Therapy Index"

**Site #:** ☐☐ **Subiect #:** ☐☐☐

## FOLLOW-UP TUMOR ASSESSMENT (RECIST v1.1)

**Instructions:** The same assessment methods used at baseline must be used for all required follow-up disease assessments. Target lesions and non-target lesions followed must be the same ones selected at baseline. Objective tumor response will be determined from imaging time point to imaging time point using the RECIST v1.1 criteria.

**Imaging Period:** ☐ Month 3 ☐ Month 6 ☐ Month 9 ☐ Month 12 ☐ Off Study

### NEW LESIONS ☐ NONE

| Lesion # | Description of New Lesion | Lesion Location [1] | Assessment Date (DD-MMM-YYYY) | Method of Assessment [2] |
|---|---|---|---|---|
| P1 | | | | |
| P2 | | | | |
| P3 | | | | |
| P4 | | | | |
| P5 | | | | |

### OVERALL RESPONSE ASSESSMENT

☐ Complete Response (CR) ☐ Partial Response (PR) ☐ Stable Disease (SD)

☐ Progressive Disease (PD) due to (check all that apply)

- ☐ >20% Increase in sum of diameters of target lesions
- ☐ Progression of existing non-target lesions
- ☐ New lesion
- ☐ Symptomatic deterioration

☐ Not All Lesions Evaluated

Reason: ____________________________________________

### COMMENTS ON TUMOR ASSESSMENT ☐ NONE

[1] **Lesion Locations:**

L1 = Bone
L2 = Brain
L3 = Breast
L4 = Liver
L5 = Lung
L6 = Lymph Node
L7 = Skin
L8 = Effusions / Ascites
L9 = GI (specify): ____________________
L10 = GU (specify): ____________________
L11 = Other (specify): ____________________

[2] **Method of Assessment:**

A1 = Clinical Exam
A2 = Chest / Abdomen X-Ray
A3 = CT scan (non-spiral)
A4 = Spiral CT Scan
A5 = MRI scan
A6 = Bone scan
A7 = PET scan
A8 = Ultrasound
A9 = Other (specify): ____________________

[3] **Lesion Diameter:** Record the longest diameter for each lesion. For lymph nodes, record the short axis measurement.

Ver. 21-SEP-2012 Follow-Up Tumor Assessment Form (FT01) Page 2 of 2

DISTRIBUTION: White – Veridex, LLC; Yellow – Investigator

*PRODUCTS ARE FOR RESEARCH USE ONLY AND THEIR PERFORMANCE CHARACTERISTICS ON CLINICAL SAMPLES HAVE NOT BEEN DETERMINED*

# PROTOCOL COMETI-P2-2012.0

"Characterization of CTC in Metastatic Breast Cancer Subjects using CTC-Endocrine Therapy Index"

**Site #:** ☐☐ **Subject #:** ☐☐☐

## CTC-ETI Blood Sample Requisition Form

### Section A: To Be Completed By Site

Date of Collection: _____ - _______ - _______ (DD MMM YYYY) Collection Time: ___ ___ : ___ ___ (24Hr)

Write the Subject ID and Date of Collection on the tube label and place onto the CellSave Tube!

Visit Designation: ☐ Baseline ☐ Month 1 ☐ Month 2 ☐ Month 3

☐ Off Study (Month 12 or Disease Progression, whichever occurs first)

# of CellSave Tubes Drawn: ☐ 1 ☐ 2 ☐ 3 ☐ 4 ☐ 5

(CTC-ETI Testing requires minimum blood volume of 30mL)

CellSave Tube Lot #:______________________ Expiration Date:______________________

Site Comments: ☐ NONE ☐ Sample Not Drawn ☐ Sample Drawn but Not Shipped

*IMPORTANT: Place original (white copy) in the insulated shipper with the sample and retain yellow copy for your records.*

Sample Handling: Blood samples may be drawn and shipped Monday through Thursday ONLY. Do not draw samples on a Friday or the day before a holiday.

**DO NOT REFRIGERATE OR FREEZE! PROTECT FROM EXTREME TEMPERATURES!**

**Samples must be shipped ambient via overnight courier on the day of collection directly to the Testing Laboratory using the pre-printed FedEx airway bills provided by the Sponsor.**

Ship Overnight To: (via FedEx) Attention:

Phone: Fax:
E-Mail:

### Section B: To Be Completed by Testing Laboratory

| | |
|---|---|
| Testing Laboratory: | CP2L__ (________________________________) |
| Date & Time Received (DD-MMM-YYYY & 24HR): | _____ - ________ - ________ _______ : ______ |
| Laboratory Assigned Sample ID: | CP2L__- |
| Laboratory Comments: ☐ NONE | |

Ver. 21-SEP-2012 CTC-ETI Sample Requisition Form LAB01
DISTRIBUTION: White – Study Laboratory, LLC; Yellow – Investigator

*PRODUCTS ARE FOR RESEARCH USE ONLY AND THEIR PERFORMANCE CHARACTERISTICS ON CLINICAL SAMPLES HAVE NOT BEEN DETERMINED*

# PROTOCOL COMETI-P2-2012.0

"Characterization of CTC in Metastatic Breast Cancer Subjects using CTC-Endocrine Therapy Index"

**LABORATORY ASSIGNED SAMPLE ID:** C P 2 L ☐ - ☐☐☐☐

## CTC-ETI RESULTS FORM

**INSTRUCTIONS:** If an entire sample cannot be tested, indicate reason below. If a marker aliquot fails, specify reason(s) for failure in Laboratory Comments section and use blank row(s) to record results from back-up aliquot(s). Refer to the CRF Completion Guidelines for details on how to determine Assigned CTC Points and Assigned Biological Points, and how to classify the aliquot failures under the Laboratory Comments section.

☐ **Sample could not be tested because of Pre-Analytical error(s) (Specify):**

☐ Sample Received >96Hr After Collection ☐ Insufficient volume of blood ☐ Tubes Cracked/Broken
☐ Blood Clotted ☐ Grossly Hemolyzed ☐ Tubes arrived cold on frozen gel packs ☐ CellSave Tubes Expired
☐ Other (Specify): ____________________

**# 7.5mL Aliquots Made:** ______

| Marker Aliquot | Aliquot Failed | Date Processed (DD-MMM-YYYY) | Time Processed (24Hr) | Date Scanned (DD-MMM-YYYY) | Total # CTC (per 7.5mL) | #(%) CTC Marker + (NA if <5 CTC) # CTC | % CTC | Assigned Biological Points |
|---|---|---|---|---|---|---|---|---|
| HER2 | ☐ | | : | | | | | |
| ER | ☐ | | : | | | | | |
| Bcl-2 | ☐ | | : | | | | | |
| Ki67 | ☐ | | : | | | | | |
| | ☐ | | : | | | | | |
| | ☐ | | : | | | | | |

**Average # CTC:** ______

**Assigned CTC Points:** ______

**CTC-ETI Score (Assigned CTC Points + Assigned Biological Points):** ______

**CTC-ETI Category** (based on CTC-ETI Score): ☐ Unable to be determined (failure of one or more aliquots)
☐ Low (0 – 3) ☐ Intermediate (4 – 6) ☐ High (7 – 16)

| Aliquot | Laboratory Comments |
|---|---|
| HER2 | ☐ None *Provide details below for these selections:* ☐ Reagent / Instrument Failure ☐ Analytical Failures ☐ Unsatisfactory Sample Quality / Results ☐ Laboratory Failure ☐ Other<br>Details: ________ |
| ER | ☐ None *Provide details below for these selections:* ☐ Reagent / Instrument Failure ☐ Analytical Failures ☐ Unsatisfactory Sample Quality / Results ☐ Laboratory Failure ☐ Other<br>Details: ________ |
| Bcl-2 | ☐ None *Provide details below for these selections:* ☐ Reagent / Instrument Failure ☐ Analytical Failures ☐ Unsatisfactory Sample Quality / Results ☐ Laboratory Failure ☐ Other<br>Details: ________ |
| Ki67 | ☐ None *Provide details below for these selections:* ☐ Reagent / Instrument Failure ☐ Analytical Failures ☐ Unsatisfactory Sample Quality / Results ☐ Laboratory Failure ☐ Other<br>Details: ________ |
| | ☐ None *Provide details below for these selections:* ☐ Reagent / Instrument Failure ☐ Analytical Failures ☐ Unsatisfactory Sample Quality / Results ☐ Laboratory Failure ☐ Other<br>Details: ________ |
| | ☐ None *Provide details below for these selections:* ☐ Reagent / Instrument Failure ☐ Analytical Failures ☐ Unsatisfactory Sample Quality / Results ☐ Laboratory Failure ☐ Other<br>Details: ________ |

**Ver. 21-SEP-2012** **CTC-ETI Results Form** **LAB02**

**DISTRIBUTION: White – Veridex, LLC; Yellow – Investigator**

*PRODUCTS ARE FOR RESEARCH USE ONLY AND THEIR PERFORMANCE CHARACTERISTICS ON CLINICAL SAMPLES HAVE NOT BEEN DETERMINED*

PROPERTY OF VERIDEX, LLC ALL INFORMATION IS CONFIDENTIAL

VERIDEX LLC
a Johnson & Johnson company

# PROTOCOL COMETI-P2-2012.0

**"Characterization of CTC in Metastatic Breast Cancer Subjects using CTC-Endocrine Therapy Index"**

**Site #:** ☐☐ **Subject #:** ☐☐☐

## STRECK Blood Sample Requisition Form

### Section A: To Be Completed By Site

**Date of Collection:** ____ - ______ - ______ (DD MMM YYYY) **Collection Time:** ___ ___ : ___ ___ (24Hr)

Write the Subject ID and Date of Collection on the tube label and place onto the STRECK Tube!

**Visit Designation:** ☐ Baseline ☐ Month 1 ☐ Month 2 ☐ Month 3
☐ Off Study (Month 12 or Disease Progression, whichever occurs first)

**# of Tubes Drawn:** ☐ 0 ☐ 1

**STRECK Tube Lot #:**______________________ **Expiration Date:**______________________

**Site Comments:** ☐ NONE ☐ Sample Not Drawn ☐ Sample Drawn but Not Shipped

***IMPORTANT:** Place original (white copy) in the insulated shipper with the sample and retain yellow copy for your records.*

**Sample Handling:** Blood samples may be drawn and shipped Monday through Thursday ONLY. Do not draw samples on a Friday or the day before a holiday.

**DO NOT REFRIGERATE OR FREEZE! PROTECT FROM EXTREME TEMPERATURES!**

**Samples must be shipped ambient via overnight courier on the day of collection directly to the Testing Laboratory using the pre-printed FedEx airway bills provided by the Sponsor.**

**Ship Overnight To:** (via FedEx) Attention:

Phone: Fax:
**E-Mail:**

### Section B: To Be Completed by Testing Laboratory

| | |
|---|---|
| Testing Laboratory: | CP2L__ (______________________________) |
| Date & Time Received (DD-MMM-YYYY & 24HR): | _____ - ________ - ________ ______ : ______ |
| Laboratory Assigned Sample ID: | CP2L__- |
| Laboratory Comments: ☐ NONE | |

**Ver. 10-DEC-2014** **STRECK Sample Requisition Form** **LAB03**
**DISTRIBUTION: White – Study Laboratory, LLC; Yellow – Investigator**

# PROTOCOL COMETI-P2-2012.0

"Characterization of CTC in Metastatic Breast Cancer Subjects using CTC-Endocrine Therapy Index"

**Site #:** ☐☐ **Subject #:** ☐☐☐

## Tissue Sample Requisition Form

### Section A: To Be Completed By Site

☐ No Tissue Provided

**Primary Tissue:** ☐ Tissue Block(s) [# of blocks: ______ ]

☐ *None* ☐ Unstained Slides [# of slides: ______ ]

Description of Tissue: ______________________________________________

Date of Collection: ______ - ________ - _________
DD MMM YYYY

**Metastatic Tissue:** ☐ Tissue Block(s) [# of blocks: ______ ]

☐ *None* ☐ Unstained Slides [# of slides: ______ ]

Description of Tissue: ______________________________________________

Date of Collection: ______ - ________ - _________
DD MMM YYYY

**Site Comments:** ☐ NONE

***IMPORTANT:** Place original (white copy) in the shipper with the tissue sample(s) and retain yellow copy for your records.*

**Ship Overnight To:**
(via FedEx)

Attention: Marty Brown
Breast Oncology Tissue Bank
University of Michigan
1500 E. Medical Center Drive
Room 7130 CCC
Ann Arbor, MI 48109
Phone: 734-615-5224 / Fax: 734-647-9480
E-Mail: Breast-Oncology-Tissue-Bank@med.umich.edu

### Section B: To Be Completed by Univ. Michigan Laboratory

| | |
|---|---|
| Date Sample Received (DD-MMM-YYYY): | |
| Storage Location: | |

**Laboratory Comments:** ☐ NONE

Ver. 21-SEP-2012 Tissue Sample Requisition Form TIS01
DISTRIBUTION: White – Veridex, LLC / Study Laboratory; Yellow – Investigator

# PROTOCOL COMETI-P2-2012.0

"Characterization of CTC in Metastatic Breast Cancer Subjects using CTC-Endocrine Therapy Index"

**Site #:** ☐☐ **Subject #:** ☐☐☐

## OFF STUDY EVALUATION FORM

**Date Off Study:** ______ - _______ - _________
DD MMM YYYY

**Reason(s) for Study Discontinuation (Check all that apply)**

☐ **Unsuccessful Baseline CTC-ETI Calculation**

☐ **Completed Study (12 Months Participation)**

☐ **Disease Progression according to RECIST v1.1 criteria** Date: ______ - _________ - _________
DD MMM YYYY

☐ **Death** (specify) Date: ______ - _________ - _________
DD MMM YYYY

☐ Cancer Related ☐ Other ______________________________

☐ **Protocol Deviation/Violation not approved by Sponsor** (Protocol Deviation Form must be completed)

☐ **Subject Withdrew Consent**

☐ **Investigator Decision**

- ☐ Subject unable to comply with / complete protocol specified imaging studies
- ☐ Subject unable to comply with / complete protocol specified blood draws
- ☐ Rising serum tumor markers
- ☐ Physical signs and symptoms
- ☐ Other ______________________________

☐ **Lost to Follow-Up** Date of last contact with subject: ______ - _________ - _________
DD MMM YYYY

☐ **Other** (specify) ______________________________

______________________________

## INVESTIGATOR SIGNATURE

**I have reviewed all the data recorded on these Case Report Form pages and certify that the data is accurate and complete to the best of my knowledge:**

Principal Investigator / Sub-Investigator (Print Name): ______________________________

Signature: ______________________________ Date (DD-MMM-YYYY): _____ - ________ - ________

**Ver. 21-SEP-2012** **Off Study Form** **OFF01**

**DISTRIBUTION: White – Veridex, LLC; Yellow – Investigator**

## PROTOCOL COMETI-P2-2012.0

**"Characterization of CTC in Metastatic Breast Cancer Subjects using CTC-Endocrine Therapy Index"**

**Site #:** ☐☐ **Subject #:** ☐☐☐

### PROTOCOL DEVIATION FORM

Use this form to document any deviations from the study protocol. Use only one form per deviation. Explain deviation in detail using the Comments section.

**1. Date of Deviation:** ______ - ________ - ________
DD MMM YYYY

**2. Specify Deviation:**

☐ Did not meet Inclusion/Exclusion criteria (specify checklist # from Eligibility Form E01):
Inclusion # ______ Exclusion # ______
Explain: ____________________________________________

☐ Imaging method changed (specify imaging and timepoint): ______________________________

☐ Imaging not performed (specify imaging and timepoint): ______________________________

☐ Protocol required blood samples / labs not collected (specify samples / labs and timepoint):
____________________________________________

☐ Protocol required blood samples / labs not collected within protocol-specific window (specify samples / labs and timepoint): ______________________________

☐ Other (specify): ____________________________________________
____________________________________________
____________________________________________

**3. Did the sponsor approve the deviation?** ☐ Yes ☐ No

**4. Subject status following deviation:**

☐ Still in trial
☐ Dropped/withdrawn
☐ Currently unknown

**5. Comments:** ____________________________________________
____________________________________________
____________________________________________
____________________________________________
____________________________________________

**Ver. 21-SEP-2012** **Protocol Deviation Form** **DEV01**
**DISTRIBUTION: White – Veridex, LLC; Yellow – Investigator**

## PROTOCOL COMETI-P2-2012.0

**"Characterization of CTC in Metastatic Breast Cancer Subjects using CTC-Endocrine Therapy Index"**

**Site #:** ☐☐ **Subject #:** ☐☐☐

### UNANTICIPATED ADVERSE DEVICE EFFECTS

Instructions: Unanticipated Adverse Device Effects are unlikely in this study as this is a minimal risk, non-intervention, non-treatment protocol; however, any serious, unexpected adverse events related to the blood collection, such as syncope (fainting) or uncontrolled bleeding, could occur and must be reported. Timely, accurate, and complete reporting of this safety information is crucial for the protection of subjects. An investigator shall submit to the study Sponsor and to the reviewing IRB a report of any unanticipated adverse device effects related to the blood collection occurring during the investigation as soon as possible, but in no event later than 10 working days after investigator first learns of the effect.

☐ **No Unanticipated Adverse Events Related to Blood Collection Observed**

| | Date of Event (DD-MMM-YYYY) | Description of Event | Record any treatments or procedures performed as a result of the event | Date Sponsor Notified (DD-MMM-YYYY) | Date IRB Notified (DD-MMM-YYYY) | Date of Resolution (DD-MMM-YYYY) |
|---|---|---|---|---|---|---|
| 1 | | | | | | |
| 2 | | | | | | |
| 3 | | | | | | |

**Ver. 21-SEP-2012** **Unanticipated Adverse Device Effects** **AE01**
**DISTRIBUTION: White – Veridex, LLC; Yellow – Investigator**

*PRODUCTS ARE FOR RESEARCH USE ONLY AND THEIR PERFORMANCE CHARACTERISTICS ON CLINICAL SAMPLES HAVE NOT BEEN DETERMINED*

## PROTOCOL COMETI-P2-2012.0

*"Characterization of CTC in Metastatic Breast Cancer Subjects using CTC-Endocrine Therapy Index"*

# Enrollment/Screening Log (All Screened Subjects)

**Site #: ______ Principal Investigator: ______________________________**

| Site - Subject Number * | Subject Initials | Date Screened (DD-MMM-YYYY) | Date Consented (DD-MMM-YYYY) | Date Completed / Collected / Initiated (DD-MMM-YYYY) | | | Date D/C (DD-MMM-YYYY) | Screen Failure* or Reason for Discontinuation (D/C) from Study (reason selected must match Off Study Form OFF01) |
|---|---|---|---|---|---|---|---|---|
| | | | | Baseline Imaging | Baseline Blood Draw | Initiation of Endocrine Tx | | |
| | ___ ___ ___ | | | | | | | ☐ Ineligible for Enrollment ☐ Withdrew Consent<br>☐ Unsuccessful Baseline CTC-ETI Calculation<br>☐ Disease progression per RECIST v1.1<br>☐ Completed Study (12 Months) ☐ Death<br>☐ Unapproved Protocol Deviation / Violation<br>☐ Investigator Decision ☐ Lost to Follow-Up<br>☐ Other: ____________ |
| | ___ ___ ___ | | | | | | | ☐ Ineligible for Enrollment ☐ Withdrew Consent<br>☐ Unsuccessful Baseline CTC-ETI Calculation<br>☐ Disease progression per RECIST v1.1<br>☐ Completed Study (12 Months) ☐ Death<br>☐ Unapproved Protocol Deviation / Violation<br>☐ Investigator Decision ☐ Lost to Follow-Up<br>☐ Other: ____________ |
| | ___ ___ ___ | | | | | | | ☐ Ineligible for Enrollment ☐ Withdrew Consent<br>☐ Unsuccessful Baseline CTC-ETI Calculation<br>☐ Disease progression per RECIST v1.1<br>☐ Completed Study (12 Months) ☐ Death<br>☐ Unapproved Protocol Deviation / Violation<br>☐ Investigator Decision ☐ Lost to Follow-Up<br>☐ Other: ____________ |
| | ___ ___ ___ | | | | | | | ☐ Ineligible for Enrollment ☐ Withdrew Consent<br>☐ Unsuccessful Baseline CTC-ETI Calculation<br>☐ Disease progression per RECIST v1.1<br>☐ Completed Study (12 Months) ☐ Death<br>☐ Unapproved Protocol Deviation / Violation<br>☐ Investigator Decision ☐ Lost to Follow-Up<br>☐ Other: ____________ |

* For screening failures, enter SF and a sequential number starting with 001 (i.e. SF-001, SF-002, SF-003, etc.), and select "Ineligible for Enrollment" in last column. For enrolled subjects, enter the site number followed by a sequential number (i.e. 01-001, 01-002, 01-003, etc.). DO NOT make any copies of this log and DO NOT send to Sponsor.

**Ver. 21-SEP-2012** **Page ____ of ____**

*PRODUCTS ARE FOR RESEARCH USE ONLY AND THEIR PERFORMANCE CHARACTERISTICS ON CLINICAL SAMPLES HAVE NOT BEEN DETERMINED*

VERIDEX LLC

# PROTOCOL COMETI-P2-2012.0

*"Characterization of CTC in Metastatic Breast Cancer Subjects using CTC-Endocrine Therapy Index"*

## PROTOCOL DEVIATION LOG: Site #____ (______________________________)

| Date of Deviation (DD-MMM-YYYY) | Subject Initials | Site - Subject Number | Deviation Description | | Medical Monitor Notified? | Additional Comments |
|---|---|---|---|---|---|---|
| | | | Deviation Code | Describe and specify reason for deviation | | |
| | ___ ___ ___ | | | | ☐ Yes ☐ No | |
| | ___ ___ ___ | | | | ☐ Yes ☐ No | |
| | ___ ___ ___ | | | | ☐ Yes ☐ No | |
| | ___ ___ ___ | | | | ☐ Yes ☐ No | |
| | ___ ___ ___ | | | | ☐ Yes ☐ No | |
| | ___ ___ ___ | | | | ☐ Yes ☐ No | |

**INVESTIGATOR SIGNATURE – I certify that the above information is accurate.**

**Principal Investigator Signature:** ______________________________ **Date (DD-MMM-YYYY)** ______-________-________

**Protocol Deviation Codes:**

01 - Informed Consent procedures
02 - Inclusion/Exclusion criteria
03 - Concomitant Medication/Therapy
04 - Laboratory Assessments/Procedures
05 - Study Procedures
06 - Serious Adverse Event Reporting/ Unanticipated Adverse Effect
07 - Randomization Procedures/Study Drug Dosing
08 - Visit Schedule/Interval
09 - Efficacy Ratings
10 - Other – use only as a last resort

**Ver. 19-FEB-2013** **Page ____ of ____**

*PRODUCTS ARE FOR RESEARCH USE ONLY AND THEIR PERFORMANCE CHARACTERISTICS ON CLINICAL SAMPLES HAVE NOT BEEN DETERMINED*

## PROTOCOL COMETI-P2-2012.0

*"Characterization of CTC in Metastatic Breast Cancer Subjects using CTC-Endocrine Therapy Index"*

# SITE ___ (________________): STUDY PERSONNEL AND DUTIES SIGNATURE LOG

(Include all personnel listed on Statement of Investigator, investigators, coordinators and other personnel responsible for study procedures)

| Date (DD-MMM-YYYY) | Print Name and Title (i.e. PI, Sub-PI, Study Coordinator, Data Manager, etc.) | SIGNATURE | Initials | Significant Trial-Related Duties * | Period of Participation From (DD-MMM-YYYY) | To (DD-MMM-YYYY) | CV Collected | FD Collected |
|---|---|---|---|---|---|---|---|---|
| | | | | | | | ☐ Yes ☐ No | ☐ Yes ☐ No |
| | | | | | | | ☐ Yes ☐ No | ☐ Yes ☐ No |
| | | | | | | | ☐ Yes ☐ No | ☐ Yes ☐ No |
| | | | | | | | ☐ Yes ☐ No | ☐ Yes ☐ No |
| | | | | | | | ☐ Yes ☐ No | ☐ Yes ☐ No |
| | | | | | | | ☐ Yes ☐ No | ☐ Yes ☐ No |
| | | | | | | | ☐ Yes ☐ No | ☐ Yes ☐ No |
| | | | | | | | ☐ Yes ☐ No | ☐ Yes ☐ No |

***SIGNIFICANT TRIAL-RELATED DUTY CODES:**

1. Informed Consent
2. Medical History
3. Physical Exam
4. Vital Signs
5. Inclusion/Exclusion Criteria
6. CRF Completion
7. Source Documentation
8. Blood Collection
9. Queries
10. Recruiting/Telephone Screening
11. Screening
12. Regulatory Coordination
13. IRB Correspondence
14. Other (Specify): ____________________

**I have delegated the responsibilities listed above:**

**Signature of Principal Investigator** ______________________________**Date**______________

**Ver. 19-FEB-2013** **Page ____ of ____**

*PRODUCTS ARE FOR RESEARCH USE ONLY AND THEIR PERFORMANCE CHARACTERISTICS ON CLINICAL SAMPLES HAVE NOT BEEN DETERMINED*

## PROTOCOL COMETI-P2-2012.0

*"Characterization of CTC in Metastatic Breast Cancer Subjects using CTC-Endocrine Therapy Index"*

## SITE STUDY VISIT / MONITORING LOG

| Date of Visit (DD-MMM-YYYY) | Purpose of Visit (i.e. Study Initiation, Monitoring, Close-out, etc.) | Printed Name of Visiting Sponsor Representative | Signature of Visiting Sponsor Representative | Signature of Study Site Representative |
|---|---|---|---|---|
| | | | | |
| | | | | |
| | | | | |
| | | | | |
| | | | | |
| | | | | |
| | | | | |
| | | | | |
| | | | | |
| | | | | |
| | | | | |
| | | | | |
| | | | | |

**Ver. 21-SEP-2012** **Page ____ of ____**

*PRODUCTS ARE FOR RESEARCH USE ONLY AND THEIR PERFORMANCE CHARACTERISTICS ON CLINICAL SAMPLES HAVE NOT BEEN DETERMINED*

## PROTOCOL COMETI-P2-2012.0

*"Characterization of CTC in Metastatic Breast Cancer Subjects using CTC-Endocrine Therapy Index"*

## SITE ____ (______________________________): SITE PERSONNEL TRAINING LOG

| Date of Training (DD-MMM-YYYY) | Type of Training (I.e. Study Initiation, Protocol, GCP/SAE, etc.) | Training Material Used (Reference location and file at site. Reference version, date,…) | Printed Name of Trainer | Signature of Trainer | Printed Name of Site Personnel Trained | Signature of Site Personnel Trained |
|---|---|---|---|---|---|---|
| | | | | | | |
| | | | | | | |
| | | | | | | |
| | | | | | | |
| | | | | | | |
| | | | | | | |
| | | | | | | |
| | | | | | | |
| | | | | | | |
| | | | | | | |

**Ver. 19-FEB-2013** **Page ____ of ____**

*PRODUCTS ARE FOR RESEARCH USE ONLY AND THEIR PERFORMANCE CHARACTERISTICS ON CLINICAL SAMPLES HAVE NOT BEEN DETERMINED*

## PROTOCOL COMETI-P2-2012.0

**"Characterization of CTC in Metastatic Breast Cancer Subjects using CTC-Endocrine Therapy Index"**

# CTC-ETI SAMPLE ACCESSIONING LOG

**Laboratory:** ____________________

| Sample ID | Subject ID (Site # – Subject #) | Timepoint (i.e. Baseline, Month #, Off-Study) | Collection Date (DD-MMM-YYYY) | Date Received (DD-MMM-YYYY) | # Tubes Received | Comments |
|---|---|---|---|---|---|---|
| CP2L__ - _____ | _____ - _______ | | | | | |
| Tube Labels | | | | | | |
| CP2L__ - _____ | _____ - _______ | | | | | |
| Tube Labels | | | | | | |
| CP2L__ - _____ | _____ - _______ | | | | | |
| Tube Labels | | | | | | |
| CP2L__ - _____ | _____ - _______ | | | | | |
| Tube Labels | | | | | | |
| CP2L__ - _____ | _____ - _______ | | | | | |
| Tube Labels | | | | | | |

**Ver. 21-SEP-2012** **Page ___ of ___**

*PRODUCTS ARE FOR RESEARCH USE ONLY AND THEIR PERFORMANCE CHARACTERISTICS ON CLINICAL SAMPLES HAVE NOT BEEN DETERMINED*

## PROTOCOL COMETI-P2-2012.0

**"Characterization of CTC in Metastatic Breast Cancer Subjects using CTC-Endocrine Therapy Index"**

# CTC POSITIVE (≥5 CTC) CARTRIDGE FREEZER LOG

**Laboratory:** ______________________________________________

| Sample ID | Aliquot (i.e. ER, HER2, Bcl-2, Ki67) | Collection Date (DD-MMM-YYYY) | Cartridge ID | Date Fixed & Frozen (DD-MMM-YYYY) | Storage Location (i.e. Freezer, Shelf, Drawer, Box) | Final Status of Cartridge (i.e. Tested, Withdrawn, Destroyed [Date]) NOTE: All cartridges must be destroyed within 10 years after collection. |
|---|---|---|---|---|---|---|
| CP2L___ - _________ | | | | | | ☐ Tested ☐ Withdrawn & Destroyed<br>☐ Destroyed on (DD-MMM-YY) _____ - _______ - _____ |
| CP2L___ - _________ | | | | | | ☐ Tested ☐ Withdrawn & Destroyed<br>☐ Destroyed on (DD-MMM-YY) _____ - _______ - _____ |
| CP2L___ - _________ | | | | | | ☐ Tested ☐ Withdrawn & Destroyed<br>☐ Destroyed on (DD-MMM-YY) _____ - _______ - _____ |
| CP2L___ - _________ | | | | | | ☐ Tested ☐ Withdrawn & Destroyed<br>☐ Destroyed on (DD-MMM-YY) _____ - _______ - _____ |
| CP2L___ - _________ | | | | | | ☐ Tested ☐ Withdrawn & Destroyed<br>☐ Destroyed on (DD-MMM-YY) _____ - _______ - _____ |
| CP2L___ - _________ | | | | | | ☐ Tested ☐ Withdrawn & Destroyed<br>☐ Destroyed on (DD-MMM-YY) _____ - _______ - _____ |
| CP2L___ - _________ | | | | | | ☐ Tested ☐ Withdrawn & Destroyed<br>☐ Destroyed on (DD-MMM-YY) _____ - _______ - _____ |
| CP2L___ - _________ | | | | | | ☐ Tested ☐ Withdrawn & Destroyed<br>☐ Destroyed on (DD-MMM-YY) _____ - _______ - _____ |
| CP2L___ - _________ | | | | | | ☐ Tested ☐ Withdrawn & Destroyed<br>☐ Destroyed on (DD-MMM-YY) _____ - _______ - _____ |
| CP2L___ - _________ | | | | | | ☐ Tested ☐ Withdrawn & Destroyed<br>☐ Destroyed on (DD-MMM-YY) _____ - _______ - _____ |
| CP2L___ - _________ | | | | | | ☐ Tested ☐ Withdrawn & Destroyed<br>☐ Destroyed on (DD-MMM-YY) _____ - _______ - _____ |
| CP2L___ - _________ | | | | | | ☐ Tested ☐ Withdrawn & Destroyed<br>☐ Destroyed on (DD-MMM-YY) _____ - _______ - _____ |
| CP2L___ - _________ | | | | | | ☐ Tested ☐ Withdrawn & Destroyed<br>☐ Destroyed on (DD-MMM-YY) _____ - _______ - _____ |
| CP2L___ - _________ | | | | | | ☐ Tested ☐ Withdrawn & Destroyed<br>☐ Destroyed on (DD-MMM-YY) _____ - _______ - _____ |

**Ver. 21-SEP-2012** **Page ___ of ___**

## PROTOCOL COMETI-P2-2012.0

**"Characterization of CTC in Metastatic Breast Cancer Subjects using CTC-Endocrine Therapy Index"**

# U. MICHIGAN TISSUE SAMPLE STORAGE LOG

| Subject ID (Site # – Subject #) | Sample Type (I.e. Blocks, Slides, Both) | Collection Date (DD-MMM-YYYY) | # of Blocks and/or Slides Stored | Storage Location (I.e. Shelf, Drawer, Box Positions) | Final Status of Tissue (I.e. # Slides/Blocks Tested & Destroyed [Date], Withdrawn & All Destroyed [Date], Destroyed [Date], # Slides/Blocks Returned [Date], etc.) |
|---|---|---|---|---|---|
| _______ - _________ | | | | | |
| _______ - _________ | | | | | |
| _______ - _________ | | | | | |
| _______ - _________ | | | | | |
| _______ - _________ | | | | | |
| _______ - _________ | | | | | |
| _______ - _________ | | | | | |
| _______ - _________ | | | | | |
| _______ - _________ | | | | | |
| _______ - _________ | | | | | |
| _______ - _________ | | | | | |

**Ver. 21-SEP-2012**

**Page ___ of ___**

*PRODUCTS ARE FOR RESEARCH USE ONLY AND THEIR PERFORMANCE CHARACTERISTICS ON CLINICAL SAMPLES HAVE NOT BEEN DETERMINED*

# APPENDIX C

## Blood Sample Collection & Handling Instructions

- **ONLY COLLECT AND SHIP SAMPLES TO THE DESIGNATED STUDY LABORATORY MONDAY THROUGH THURSDAY. DO NOT DRAW SAMPLES ON A FRIDAY OR THE DAY BEFORE A HOLIDAY.**
- At each blood draw ~40mL of blood will be obtained in **5 different 10mL CellSave Preservative Tubes**. All tubes must be labeled with the Subject ID number, a visit designation (i.e. baseline, month 1, month 2, etc.) and the collection date and time.
- Inspect the tube prior to use for cracks, debris or discolored fluid inside. Do not use the CellSave Preservative Tube if it is expired, broken or appears to be contaminated.
- Perform venipuncture and, without changing the needle, draw the blood into each tube (actual volume will be ~8mL of blood per tube, and a minimum of 30mL of blood is required to complete all required assays). Fill each CellSave Preservative Tube until blood flow stops. If blood is collected through an intravenous line, ensure that line has been cleared of IV solution before beginning to fill CellSave tubes.
- Immediately after collection, gently invert each of the 5 CellSave tubes a minimum of 8 times to ensure mixture of the blood with the anticoagulant and cellular preservative. Inadequate or delayed mixing may result in clotting and inaccurate test results.
- Record the date and time of sample collection on the CTC-ETI Requisition Form. Indicate the visit and the number of tubes drawn, and record the CellSave Lot # and Expiration Date.
- **DO NOT REFRIGERATE OR FREEZE. PROTECT FROM EXTREME TEMPERATURES**. Store and transport samples at temperatures of 15-30˚C (59-86˚F). Refrigerating or freezing samples prior to processing could adversely affect sample integrity.
- Prepare the samples for shipment on the same day as collected using the shipping containers and pre-printed FedEx airway bills provided by the Sponsor:

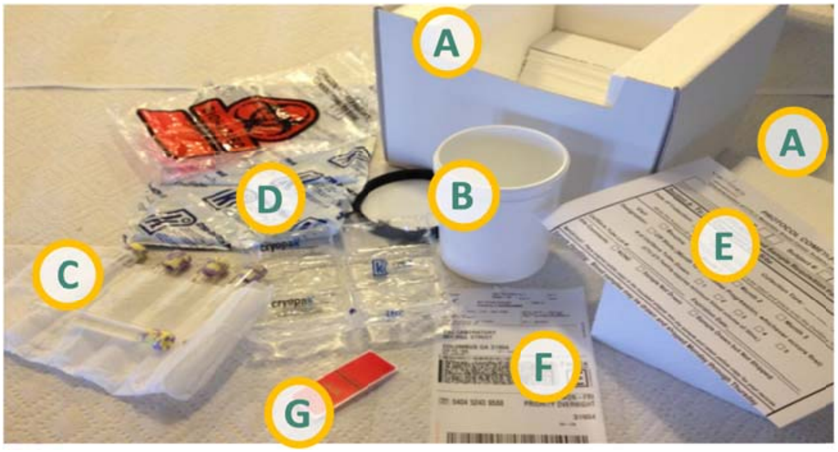


A. shipper box & Styrofoam cover
B. shipping cylinder
C. absorbent pouch & CellSave Preservative Tubes
D. Gel & water packs and biohazard bag
E. Completed requisition form (LAB01)
F. Pre-printed FedEx shipping label
G. Box sealing tape

*PRODUCTS ARE FOR RESEARCH USE ONLY AND THEIR PERFORMANCE CHARACTERISTICS ON CLINICAL SAMPLES HAVE NOT BEEN DETERMINED*

- Insert the CellSave Preservative Tubes into the separate pockets of the absorbent pouch.

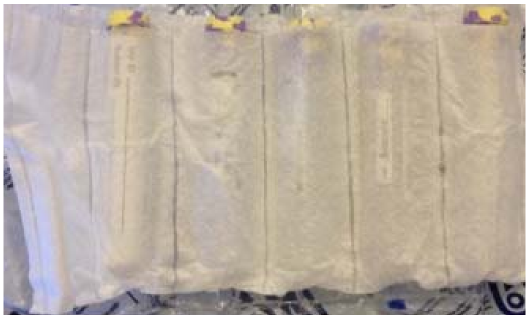

- Place the pouch inside of the biohazard bag, remove all air from inside the biohazard bag and seal at the top.

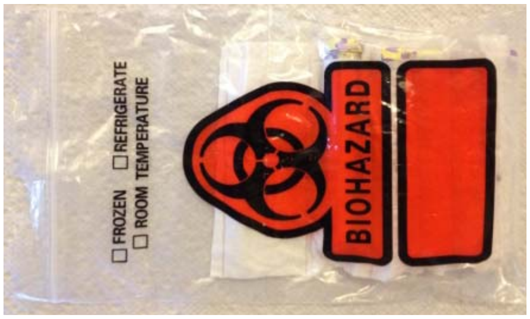


- Place the water pack on top of the gel pack, and then place the biohazard bag (with the tubes inside the absorbent pouch) on top of the water pack.

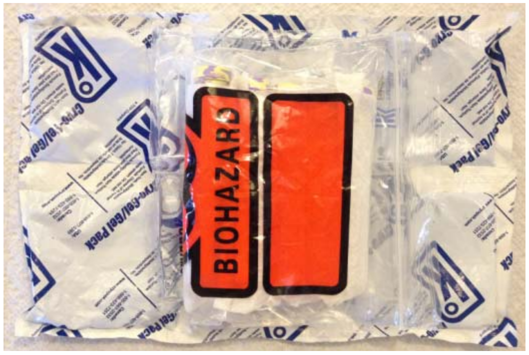


- Roll the biohazard bag containing the filled blood tubes inside the gel and water packs and place the packaged tubes inside the shipping cylinder.

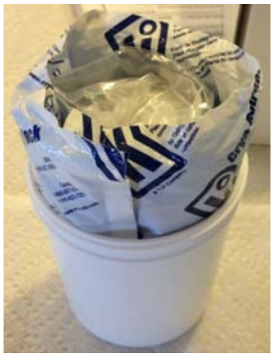

- Screw cap onto the shipping cylinder and place the capped shipping cylinder inside the Styrofoam shipping box.

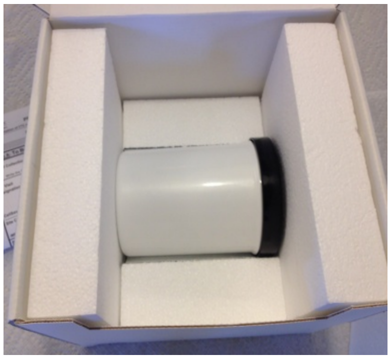

- Place Styrofoam cover on top of shipping cylinder and place original copy of completed requisition form (LAB01, **Appendix B**) on top of Styrofoam cover.

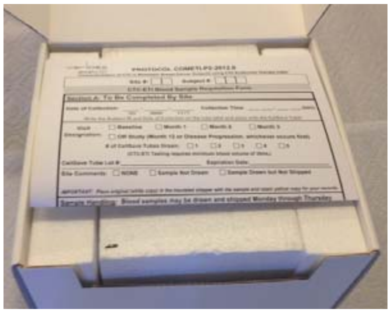

- Close and seal cardboard box using sealing tape and place FedEx shipping label on outside of cardboard box.

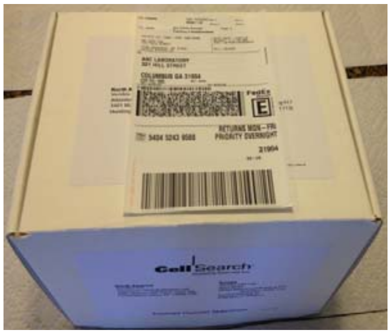


- Arrange for pickup by calling 1-800-463-3339 (1-800-GoFedEx). Notify the designated study laboratory by email with your site number, your contact information, and the shipment tracking number. See separate Contact Information Sheet for study laboratory information.

# APPENDIX D

## Tissue Sample Collection & Handling Instructions

After a subject has been enrolled into the study, the research nurse or the study coordinator will call the Pathology Department at their own Institution.

The pathologist is responsible for:

- Obtaining pathology reports of primary and metastatic lesions.
- Pulling the tissue blocks and/or the slides from the primary and any metastatic lesions.
- Reviewing the tissue block and slides and choosing **a non–necrotic representative area of the tumor**.

A) Send tissues per patient for BOTH primary and metastatic tumors. If it is not possible to send both, send one or the other.

B) If sending a tissue block, include the following in shipment:

1. One (1) representative paraffin embedded block containing sufficient tumor tissue.
2. One (1) standard H&E (Hematoxylin and Eosin) stained slide from the representative paraffin embedded block.
3. A pathology report (indicating the morphological diagnosis).

C) If sending unstained slides, include the following in shipment:

1. Ten (10) unstained positively charged slides consisting of 5µm tissue sections from the representative paraffin embedded tissue block containing sufficient tumor tissue.
2. One (1) standard H&E (Hematoxylin and Eosin) stained slide from the representative paraffin embedded block.
3. A pathology report (indicating the morphological diagnosis).

Tissue specimens must be shipped to the University of Michigan laboratory at the following address:

Attention: Marty Brown
Breast Oncology Tissue Bank
University of Michigan
1500 E. Medical Center Drive
Room 7130 CCC
Ann Arbor, MI 48109
Phone: 734-615-5224
Fax: 734-647-9480
E-mail: Breast-Oncology-Tissue-Bank@med.umich.edu

Each specimen should be accompanied by a tissue specimen form that has been accurately filled out (**Appendix B**).

The tissue blocks will be returned to the submitting Pathology Department of each Institution after samples have been obtained for the study.

# APPENDIX E: Additional rules for CTC-ETI calculation

**PRE-ANALYTICAL ERRORS:**

- Insufficient volume of blood for processing (i.e. <30mL of pooled whole blood)
- Blood clotted or grossly hemolyzed
- Sample received >96 hours (4 days) after the collection of the blood from the subject
- Tubes arrive cold on frozen gel paks
- Tubes broken during shipment or received cracked
- Blood collected in expired CellSave Preservative Tubes

**An aliquot can be considered as a failed aliquot for one or more of the following reasons:**

**UNRESOLVED TECHNICAL FAILURES:**

- ***Reagent and Instrument Failures:***
  - Sample is aborted on the CELLTRACKS® AUTOPREP® instrument and processing is not completed.
  - Sample is not fully transferred to the cartridge (i.e. fluid left in the CELLTRACKS® AUTOPREP® tube after transferring of the processed sample into the cartridge).
  - Marker control failures (such as no positive staining, widespread non-specific staining, etc.).
  - No CTC are identified and ferrofluid is left in the CELLTRACKS® AUTOPREP® tube after transferring of the processed sample into the cartridge.
  - CXC control sample for the batch/instrument fails, and subsequent controls run within 24 hours also fail.

- ***Unsatisfactory Sample Quality and Results:***
  - Sample unable to be scanned on CELLTRACKS ANALYZER II® due to inability to focus because of interfering substances or obscuring nuclear material visible in the cartridge.
  - Sample unable to be scanned on CELLTRACKS ANALYZER II® due to acellular specimen (unable to focus).
  - No CTC are identified and there are interfering substances and/or obscuring nuclear material visible in CellSelect.
  - No CTC are identified and there is scant cellularity visible in the cartridge and the cartridge fluid is clear.
  - Severe ferrofluid aggregation is visible in the cartridge, making sample non-reviewable.

- ***Analytical Failures:***
  - Irresolvable discordance as described below in "Considerations for Calculation of CTC-ETI" in CTC count in one or more of the four aliquots, making the CTC-ETI calculation unsuccessful.

## LABORATORY FAILURES:

- Failure to process sample within 96Hr (4 Days) after collection if received within 96Hr after collection.
- Failure to scan cartridge within 24Hr (1 Day) of processing.
- Failure to repeat a failed control within 24Hr of initial run.
- Use of expired reagents

## <u>Considerations for calculation of CTC-ETI</u>

- *Three or more aliquots fail* (i.e. instrument failure, unsatisfactory results, etc.):
  - The sample will be considered unsuccessful for CTC-ETI calculation.
- *Two or more aliquots fail* (i.e. instrument failure, unsatisfactory results, etc.):
  - If 2 backup aliquots are not available for re-testing, the sample will be considered unsuccessful for CTC-ETI calculation.
  - If the 2 backup aliquots are available, discard the original 2 failed results, run the backups with the appropriate marker reagents and proceed to determine the CTC Assigned Points and the CTC Biologic Points using the results from the two backup aliquots.
- *One aliquot fails but the other three aliquots provide CTC enumeration and Biologic Points results:*
  - If no backup aliquot is available for re-testing, the sample will be considered unsuccessful for CTC-ETI calculation.
  - If a backup aliquot is available, run the backup with the appropriate marker reagent and proceed as described in the detailed examples below to determine the CTC Assigned Points and Biologic Points.
- *All four aliquots provide evaluable CTC results:*
  - If all four CTC counts are $\geq$5 CTC/7.5mL, round up the average CTC count (see **Figure E1** below) to determine the corresponding CTC Assigned Points and calculate the proportion of CTC positive for each marker to determine the CTC Biologic Points. Combine the CTC Assigned Points and CTC Biologic Points to determine the CTC-ETI Score and final Category.
  - If all four CTC counts are <5 CTC/7.5mL: Average CTC Count = CTC 0-4, CTC Assigned Points = 0, CTC-ETI Score = 0, and CTC-ETI Category = Low.
  - If average CTC count is <5 and two CTC counts are <5 CTC/7.5mL and two CTC counts are ≥5 CTC/7.5mL, the average enumeration will be rounded down (see figure below) Average CTC Count = CTC 0-4, CTC Assigned Points = 0, CTC-ETI score = 0, and CTC-ETI Category = Low. For example, a subject whose average CTC count is calculated as 4.75 {3, 3, 6, 7} or is calculated as 4.25 {2, 5, 6, 4}.

**Figure E1**

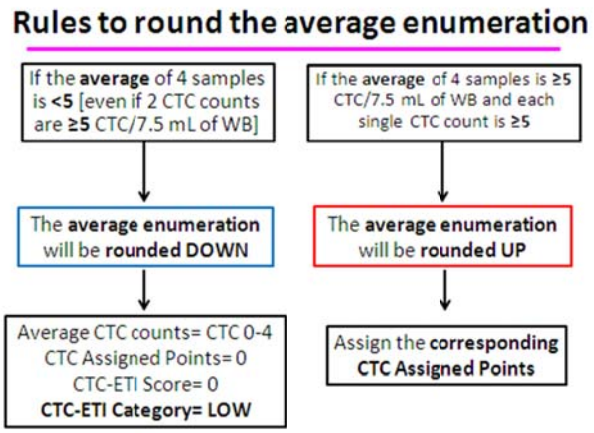


- If the average CTC count is ≥5 CTC/7.5mL and two CTC counts are <5 CTC and two CTC counts are ≥5 CTC/7.5mL (see **Figure E2**):
  - If the 2 backup aliquots are available, they will be used to replace the 2 low outliers (<5 CTC). They will be stained for the same markers as the original low outliers.
    - ➢ If both backup samples' CTC counts are ≥5 CTC/7.5mL, these data will be used for both average enumeration and CTC Bio-Score for the respective markers.
    - ➢ If only one of the backup sample's CTC count is ≥5 CTC/7.5mL, and the second backup sample's CTC count is <5 CTC/7.5mL:
      - ✓ For the average enumeration: CTC counts of the original AND backup outliers (i.e.<5 CTC) will be discarded and not used for calculating average enumeration. Use the CTC counts from the 2 originals with ≥5 CTC and the backup aliquots with ≥5 CTC backup to determine average CTC count.
      - ✓ For the staining:
        - For the backup sample with CTC count ≥5 CTC/7.5mL, these data will be used for CTC Bio-Score for that respective marker.
        - For the second backup with CTC count <5 CTC/7.5mL:
          a. If the combination of CTC count of both the original and the backup is ≥5 CTC/7.5 mL of WB for that respective marker, then the original sample and the backup sample staining will be combined and used as a single set of staining data for Bio-Score analysis.
          b. If the combination of CTC count of both the original and the backup is <5, then the data cannot be used for staining. Therefore, the sample will be considered unsuccessful for CTC-ETI calculation.
    - ➢ If both backup samples' CTC counts are <5 CTC/7.5mL: the 2 original aliquots with ≥5 CTC/7.5mL are now considered outliers and CTC-ETI score = 0, and CTC-ETI Category = Low
  - If only one backup aliquot is available for re-testing, don't run it. The sample will be considered unsuccessful for CTC-ETI calculation.

**Figure E2**

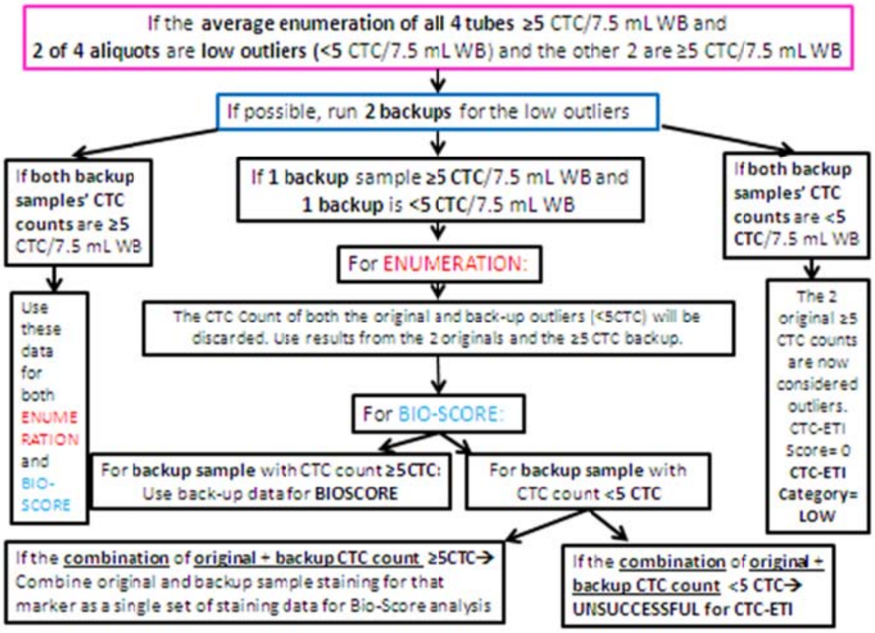


- If the average CTC count is ≥5 CTC/7.5mL and three CTC counts are ≥5 CTC/7.5mL and one is a low outlier (see **Figure E3**):
  - If a backup aliquot is not available, the sample will be considered unsuccessful for CTC-ETI calculation.
  - If a backup aliquot is available, run the backup with the appropriate marker reagent.
    - ➢ If the backup aliquot fails (i.e. instrument failure, unsatisfactory results, etc.), the sample will be considered unsuccessful for CTC-ETI calculation.
    - ➢ If the CTC count for the backup aliquot is ≥5 CTC, the enumeration and marker results from the backup aliquot will be used to determine the CTC Assigned Points and Biologic Points and the results from the original aliquot with <5 CTC will be discarded.
    - ➢ If the CTC count for the backup aliquot is <5 CTC:
      - ✓ If the combination of the CTC count from the original aliquot and the backup aliquot is <5 CTC, then the sample will be considered unsuccessful for CTC-ETI calculation.
      - ✓ If the combination of the CTC count from the original aliquot and the backup aliquot is ≥5 CTC, then:
        - For CTC Assigned Points: Determine the average CTC count using the CTC counts from the three original aliquots with ≥5 CTC (round up), and;
        - For the CTC Biologic Points: Combine the results from the original and backup aliquots (marker results) to determine the CTC Biologic points for that marker.

**Figure E3**

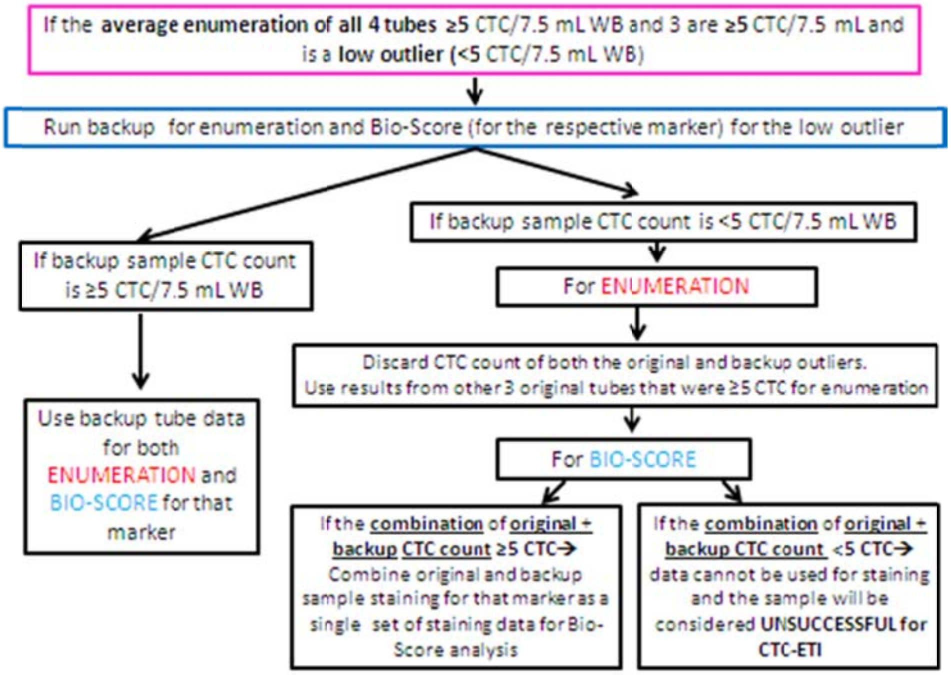


- If the average CTC count is <5 CTC/7.5mL and one of 4 aliquots is a high outlier (≥5 CTC/7.5 ml WB) and the other three CTC counts are <5 CTC/7.5mL (see **Figure E4**):
  - If a backup aliquot of blood is available, run the backup with the appropriate marker reagent.
    - ➢ If after using the backup sample the average enumeration of the 3 original CTC counts (<5 CTC/7.5mL) plus the backup CTC count is <5 CTC: CTC Assigned Points=0 and CTC-ETI Category= Low.
    - ➢ If after using the backup sample the average enumeration of the 3 original CTC counts (<5 CTC/7.5mL) plus the backup CTC count is ≥5 CTC, the sample will be considered unsuccessful for CTC-ETI calculation.

**Figure E4**

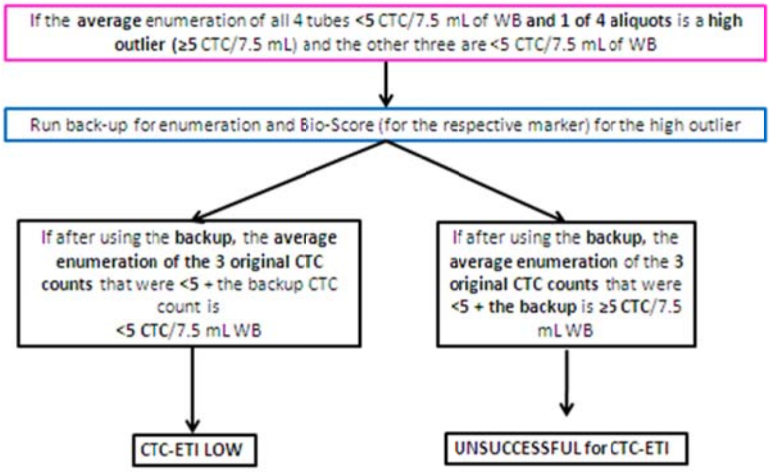

# CONTACT INFORMATION SHEET

**Sponsor Contacts:**

Robert McCormack, Ph.D.
Senior Director, Oncology Biomarkers
Janssen R&D
700 Route 202 South, Room 1055
Raritan, NJ 08869
Phone: 908-927-4824
Mobile: 908-625-2588
E-Mail: rmccorma@its.jnj.com

Scott Adams, MS
Associate Director, Clinical Research
Janssen R&D
700 US Highway 202 S, 1114
Raritan, NJ 08869
Phone: 908-927-4702
E-Mail: sadams17@its.jnj.com

**Principal Investigator Contact:**

Daniel F. Hayes, M.D.
Clinical Director, Breast Oncology Program
Stuart B. Padnos Professor in Breast Cancer
University of Michigan Comprehensive Cancer Center
6312 Cancer Center
1500 E. Medical Center Dr.
Ann Arbor, MI 48109-0942
Phone: 734-615-6725
Fax: 734-647-9271
E-Mail: hayesdf@med.umich.edu

**Co-Principal Investigator Contact:**

Costanza Paoletti, M.D.
Research Fellow
Department of Internal Medicine
Division of Hematology Oncology
University of Michigan Comprehensive Cancer Center (UM CCC)
7130 CCC, SPC 5948
1500 E. Medical Center Drive
Ann Arbor, MI, U.S.A. 48109-5948

Phone: 734-647-7250
Fax: 734-647-9480 or 734-647-9271
E-Mail: pcostanz@med.umich.edu

**Statistical Contact:**

Meredith M. Regan, ScD
Department of Biostatistics and Computational Biology
Dana-Farber Cancer Institute
450 Brookline Ave
CLS11007
Boston, MA 02215
Phone: 617-632-2471
E-Mail: mregan@jimmy.harvard.edu
