# Supplementary material for: Circulating tumor cell number and endocrine therapy index in ER positive metastatic breast cancer patients
Source: NPJ Breast Cancer. 2021 Jun 11;7:77. doi: 10.1038/s41523-021-00281-1 (PMC8196036; doi:10.1038/s41523-021-00281-1)
Supplement: Supplementary file 2 — Reporting Summary [file 41523_2021_281_MOESM2_ESM.pdf]

nature research

Corresponding author(s): Daniel F. hayes

Last updated by author(s): Feb 22, 2021

Reporting Summary

Nature Research wishes to improve the reproducibility of the work that we publish. This form provides structure for consistency and transparency in reporting. For further information on Nature Research policies, see our Editorial Policies and the Editorial Policy Checklist.

Statistics

For all statistical analyses, confirm that the following items are present in the figure legend, table legend, main text, or Methods section.

n/a	Confirmed	
☐	☒	The exact sample size (*n*) for each experimental group/condition, given as a discrete number and unit of measurement
☒	☐	A statement on whether measurements were taken from distinct samples or whether the same sample was measured repeatedly
☐	☒	The statistical test(s) used AND whether they are one- or two-sided<br>*Only common tests should be described solely by name; describe more complex techniques in the Methods section.*
☐	☒	A description of all covariates tested
☐	☒	A description of any assumptions or corrections, such as tests of normality and adjustment for multiple comparisons
☐	☒	A full description of the statistical parameters including central tendency (e.g. means) or other basic estimates (e.g. regression coefficient) AND variation (e.g. standard deviation) or associated estimates of uncertainty (e.g. confidence intervals)
☐	☒	For null hypothesis testing, the test statistic (e.g. *F*, *t*, *r*) with confidence intervals, effect sizes, degrees of freedom and *P* value noted<br>*Give P values as exact values whenever suitable.*
☒	☐	For Bayesian analysis, information on the choice of priors and Markov chain Monte Carlo settings
☒	☐	For hierarchical and complex designs, identification of the appropriate level for tests and full reporting of outcomes
☒	☐	Estimates of effect sizes (e.g. Cohen's *d*, Pearson's *r*), indicating how they were calculated

Our web collection on statistics for biologists contains articles on many of the points above.

Software and code

Policy information about availability of computer code

Data collection	Clinical data were collected on paper CRFs and managed by Veridex/Janssen in Medidata RAVE.
Data analysis	The statistical analysis used SAS v8 and v9 (SAS Institute, Cary, NC).

For manuscripts utilizing custom algorithms or software that are central to the research but not yet described in published literature, software must be made available to editors and reviewers. We strongly encourage code deposition in a community repository (e.g. GitHub). See the Nature Research guidelines for submitting code & software for further information.

Data

Policy information about availability of data

All manuscripts must include a data availability statement. This statement should provide the following information, where applicable:

- Accession codes, unique identifiers, or web links for publicly available datasets
- A list of figures that have associated raw data
- A description of any restrictions on data availability

The datasets generated and analyzed supporting the findings of this study are available from the corresponding author upon reasonable request.

Field-specific reporting

Please select the one below that is the best fit for your research. If you are not sure, read the appropriate sections before making your selection.

☒ Life sciences ☐ Behavioural & social sciences ☐ Ecological, evolutionary & environmental sciences

For a reference copy of the document with all sections, see nature.com/documents/nr-reporting-summary-flat.pdf

Life sciences study design

All studies must disclose on these points even when the disclosure is negative.

Sample size	A sample size of approximately 200 subjects was determined to provide a sufficient number of evaluable subjects and statistical power for evaluation of the primary and secondary objectives. We estimated that a total of 120 subjects evaluable for clinical validity analyses would be required to obtain the minimum of 51 subjects with rapid progression for evaluation of the clinical validity of the CTC-ETI.
Data exclusions	According to protocol, five patients' samples were excluded because of pre-analytical errors (N=4) or patient ineligibility (N=1) determined prior to processing. Those five patients ended study participation.<br><br>An additional nine patients were excluded from the clinical analyses: three because of unsuccessful baseline CTC-ETI and six for protocol deviation/violation.
Replication	N/A for this prospective pilot clinical trial. The prognostic role of circulating tumor cells has been shown in several other manuscripts.
Randomization	This study was not randomized and treatment recommendations were left to the patients' oncologists, making this study more generalizable to clinical practice.
Blinding	CTC-ETI analysis was performed blindly by two independent operators at UM without knowledge of clinical data. Likewise, clinical decisions were made by caregivers without knowledge of CTC-ETI.

Reporting for specific materials, systems and methods

We require information from authors about some types of materials, experimental systems and methods used in many studies. Here, indicate whether each material, system or method listed is relevant to your study. If you are not sure if a list item applies to your research, read the appropriate section before selecting a response.

Materials & experimental systems

n/a	Involved in the study	
☐	☒	Antibodies
☒	☐	Eukaryotic cell lines
☒	☐	Palaeontology and archaeology
☒	☐	Animals and other organisms
☐	☒	Human research participants
☐	☒	Clinical data
☒	☐	Dual use research of concern

Methods

n/a	Involved in the study	
☒	☐	ChIP-seq
☒	☐	Flow cytometry
☒	☐	MRI-based neuroimaging

Antibodies

Antibodies used	ER: monoclonal murine ER-119.3; Menarini Silicon Biosystems, Inc.<br><br>BCL2: monoclonal murine BCL-2/ (100); BD Pharmingen<br><br>HER2: monoclonal murine Her81; Menarini Silicon Biosystems, Inc.<br><br>Ki67: monoclonal murine B56; BD Pharmingen
Validation	Paoletti C, Muniz MC, Thomas DG, Griffith KA, Kidwell KM, Tokudome N, Brown ME, Aung K, Miller MC, Blossom DL, Schott AF, Henry NL, Rae JM, Connelly MC, Chianese DA, Hayes DF. Development of circulating tumor cell-endocrine therapy index in patients with hormone receptor-positive breast cancer. Clin Cancer Res. 2015;21(11):2487-98. doi: 10.1158/1078-0432.CCR-14-1913. PubMed PMID: 25381338.

Human research participants

Policy information about studies involving human research participants

Population characteristics	The one-hundred seven patients included in the analyses of clinical validity (Table 1) had a median age of 63 (range 33-84). All patients had ER positive breast cancer, either in their primary or metastatic tumors. Of note, 1 patient had an ER and PgR negative, and an additional 7 patients had ER and PgR unknown primary cancers. Each of these 8 patients had ER positive metastatic biopsies.<br>Of the 107 eligible patients, 25 (23%) had developed metastases while on or within 12 months of completing adjuvant ET, and 50 (47%), 21 (20%), and 11 (10%) had received 1, 2, or ≥3 lines of ET for MBC, respectively. CTC enumeration did not significantly differ among these groups (Table 1). According to the advanced breast cancer 3 (ABC 3) definition(7), at the time of the study enrollment, 12 (11%) and 95 (89%) of the 107 patients had primary endocrine resistance or secondary, acquired endocrine resistance, respectively. Thirty-two (30%) patients had bone lesions only; and 59 (55%) had a measurable disease according to RECIST criteria. During the trial, 54 (51%), 42 (39%), 12 (11%) and 2 (2%) patients were treated with fulvestrant alone or in combination, aromatase inhibitors alone or in combination, tamoxifen, or another ET, respectively, and 26 (24%) were treated with ET plus either palbociclib (N=12) or everolimus (N=14).
Recruitment	Female subjects 18 years or older with ER positive, HER2 negative, progressive MBC after one or more lines of ET who are initiating a new ET were enrolled into the study. Subjects must have immunohistochemistry (IHC) proven ER positive disease, IHC and/or fluorescence in-situ hybridization (FISH) proven HER2 negative disease, and an ECOG performance status of 0-2. Subjects with brain metastases only or those who are progressing on fulvestrant were not eligible for the study. All subjects must be informed of the investigational nature of this study and be willing to provide written informed consent in accordance with Institutional guidelines and Good Clinical Practice (GCP).
Ethics oversight	The study protocol was approved by the Institutional Review Board of each participating center.

Note that full information on the approval of the study protocol must also be provided in the manuscript.

Clinical data

Policy information about clinical studies

All manuscripts should comply with the ICMJE guidelines for publication of clinical research and a completed CONSORT checklist must be included with all submissions.

Clinical trial registration	NCT01701050
Study protocol	Study protocol is included as supplementary material with this submission.
Data collection	Between April 2013 and November 2015, pre-treatment (baseline) samples were collected from 121 patients enrolled at 19 centers in North America.
Outcomes	Progression-free survival (PFS) was measured as the time from the date of baseline sample until the date of first documentation of progressive disease according to RECIST v1.1 criteria, or death due to any cause. In absence of these events, PFS was censored at the date of the last objective assessment (up to a maximum of 12 months after the initiation of ET). Rapid progression (RP) was defined as the presence or absence of objective radiographic progression according to RECIST v1.1 criteria or death due to MBC within 3 months. Patients without re-imaging at 3 months to determine RP status were omitted from the analysis, even in the situation of symptomatic deterioration or rising serum tumor markers (i.e. CA 15-3/27.29 or CEA).
